# Copy number variations: The potential association genetic cause in severe cardiovascular diseases with unknown aetiology

**DOI:** 10.1111/jcmm.18461

**Published:** 2024-06-22

**Authors:** Niloofar Naderi, MohammadHossein MozafaryBazargany, Majid Maleki, Samira Kalayinia

**Affiliations:** ^1^ Cardiogenetic Research Center, Rajaie Cardiovascular Medical and Research Center Iran University of Medical Sciences Tehran Iran; ^2^ Rajaie Cardiovascular Medical and Research Center, Iran University of Medical Sciences Tehran Iran

**Keywords:** aortic aneurysm, cardiomyopathy, cardiovascular disease, CGH array, channelopathy, congenital heart disease, copy number variation, genetic, hypercholesterolaemia, hypertension

## Abstract

Cardiovascular diseases (CVDs) are the leading cause of mortality worldwide. While both genetic and environmental factors significantly contribute to the pathogenesis of CVDs, recent advancements in genetic technology have further emphasized the significance of genetic factors in CVDs. Growing evidence suggests genetic changes as a primary cause of CVDs and their susceptibility. The major genetic changes include chromosomal aneuploidy, abnormal chromosomal structure, and single gene mutations. Copy number variation (CNV) is a type of newly introduced structural change that influences the copy number of a genome region. CNVs could manifest with more severe phenotypes compared to single‐nucleotide polymorphism (SNP) as they affect a considerably larger segment of the genome. Additionally, for the same reason, CNVs tend to manifest earlier in life compared to SNP. Recent studies have demonstrated the fundamental role of CNVs in the development or susceptibility to cardiovascular disorders. However, it remains to be determined which CVDs should prompt CNV tests in clinical practice. In this review, we discussed the extent to which CNV could take part in CVDs and further hypothesized that testing for CNV might be most beneficial for selective patients with CVDs.

## 1 INTRODUCTION

Cardiovascular diseases (CVDs), recognized as multifactorial disorders, are the primary cause of death worldwide.^1^, ^2^, ^3^ According to the World Health Organization (WHO) report, CVD accounted for 17.9 million deaths globally in 2016.^4^ The prevalence of CVDs in adults over 20 years of age is 48%, with the risk being the equivalent for both males and females.^5^ CVDs include familial hypercholesterolaemia (FH), hereditary cardiomyopathies, sudden cardiac death (SCD) and channelopathies, congenital heart disease (CHD), thoracic aortic aneurysms and dissections, coronary artery disease (CAD), and stroke.^6^, ^7^ CVDs can be triggered by clinical (hypertension, dyslipidaemia, diabetes mellitus and genetic) or behavioural (sedentary lifestyle, smoking and stress) factors.^8^, ^9^, ^10^ Numerous studies have highlighted that genetic changes are a principal significant cause of CVDs.^11^, ^12^, ^13^ The major genetic changes include chromosomal aneuploidy, abnormal chromosomal structure and single gene mutations. The copy number variations (CNVs), as one of the structural changes that influence the dosage of a specific genome region, can lead to severe phenotypes with early onset.^14^ CNVs play a pivotal role in causing many diseases, such as CVDs, HIV/AIDS susceptibility, rheumatoid arthritis, lupus nephritis, osteoporosis, Parkinson's disease, psoriasis, Crohn's disease, autism spectrum disorders, Charcot–Marie–Tooth neuropathy type 1A, acute myeloid leukaemia (AML) and bipolar disorder.^15^ In this review, for the first time, we aimed to survey and discuss recent publications to elucidate the role of CNVs in the aetiology of CVDs and the importance of their examination in severe CVD cases.

## 2 CNVS DEFINITION AND CLASSIFICATION

Genomic structural variants (SVs) are alterations in the chromosome sequence greater than 50 bp and encompass CNVs, deletions, duplications, insertions, inversions and translocations.^16^, ^17^, ^18^ CNVs, which either increase or decrease the copy numbers of specific genomic regions, play a crucial role in the onset of many diseases, such as CVDs.^15^ Studies have indicated that approximately 12%–16% of the human genome sequence contains CNVs,^19^, ^20^, ^21^ which can occur during germ line meiosis or somatic mitosis.^21^ While several genetic risk factors contribute to the development of CVDs, the specific role of CNVs remains to be fully elucidated.^15^

In 2020, a semi‐quantitative, score‐based scoring criterion was introduced by the American College of Medical Genetics and Genomics (ACMG) and Clinical Genome Resources (ClinGen) to enhance consistency in the CNV interpretation.^22^ The classification of CNVs is based on a compound of the ACMG updated guidelines and standards for the description and reporting of postnatal CNVs.^23^ CNVs are classified into the following categories: pathogenic, likely pathogenic, variant of uncertain significance (VUS), benign or likely benign.^22^, ^24^, ^25^ Pathogenic CNVs, with an evidence score of 0.99 or higher, indicate a high probability of being disease‐causing, although the CNV's full clinical effect on a patient's phenotype may not be completely understood; this does not negate the CNV's potential pathogenicity.^26^ Likely pathogenic CNVs with an evidence score between 0.90 and 0.98 have strong evidence suggesting pathogenicity but are not yet sufficient to conclusively determine pathogenicity.^26^ VUS CNVs, with an evidence score between −0.89 and 0.89, represent a broad category where, if there are insufficient reasons to definitively confirm clinical significance, the CNV should be classified as a VUS.^26^ Likely benign CNVs, with an evidence score between −0.90 and −0.98, are unlikely to be associated with Mendelian diseases, yet insufficient evidence exists to definitively confirm this.^26^ Benign CNVs, with an evidence score of −0.99 or lower, are generally classified as benign variants in publications or databases. In addition, a CNV should be classified as a benign polymorphism when its minor allele frequency (MAF) exceeds 1% in the population.^26^

## 3 CNV MECHANISM IN HUMAN GENOME

**FIGURE 1:**
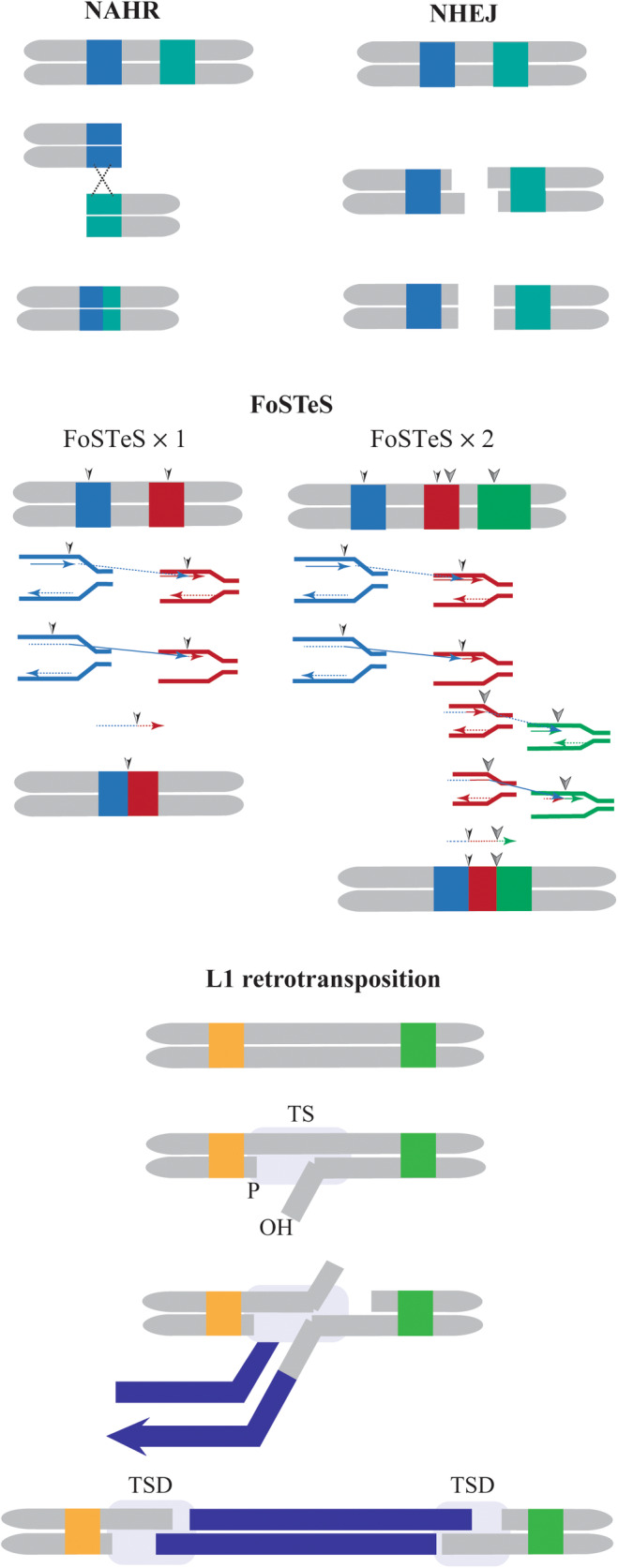
Investigation and comparison of four main mechanisms in the field of human genomic rearrangement and copy number variation (CNV) formation in genomic deletion/duplication. (A) NAHR: intrachromatid non‐allelic homologous recombination (NAHR) models between repetitive sequences (LCRs/SDs, Alu or L1 elements). Coloured rectangles are two direct‐directed low‐copy repeats (LCRs) with high homology (97%–98%), which align at non‐allelic positions, and subsequent recombination results in deletes or duplicates of a protein part of the two LCRs or also their flanking part. (B) NHEJ: A non‐homologous end‐joining (NHEJ) event involves the recombination repair of double‐strand break (DSBs) between two sequences that do not share any homology. This system changes the two ends and reconnects them together, thus removing the piece between the two DSBs. (C, D) FoSTeS: A fork stalling and template switching (FoSTeS), single FoSTeS event (×1) causing simple rearrangement and multiple FoSTeS events (×2) resulting in complex rearrangement and retro transposition. FoSTeS occurs during DNA replication against NAHR and NHEJ. The leading nascent strand at the left fork attacks the right fork and primers its next synthesis using the right fork as a template. This event occurs twice, removing fragments and flanking each pair of microhomology sites.^127^ TS target site; TSD, duplicated target site.

Different mechanisms, such as DNA recombination, repair and replication can lead to duplication or truncation of gene segments, which often results in non‐functional products.^27^ Several molecular mechanisms, both replication and non‐replication, contribute to the CNV development. Non‐replication mechanisms including non‐allelic homologous recombination (NAHR) in regions of extensive sequence similarity, non‐homologous end joining (NHEJ) and microhomology‐mediated end joining (MMEJ), while replicative mechanisms involve replication slippage, fork stalling, template switching (FoSTeS), microhomology‐mediated break‐induced replication (MMBIR), transposable element (TE) and L1 retrotransposition.^28^, ^29^ NAHR, a recombination‐based mechanism in humans, is characterized by frequent homologous rearrangements.^29^ The integration of a genomic segment with high sequence similarity to another non‐allelic locus causes CNV through NAHR, resulting in duplication in one chromosome region and deletion in the corresponding region of the other chromosome.^30^ Telomere fusion between two chromosomes and the formation of a bicentric chromosome occurs via the NHEJ pathway.^31^ The pathway facilitates the identification of genetic factors and the examination of variations in genes.^32^ The MMEJ pathway causes chromosomal translocation and telomere fusion, followed by alternative NHEJ.^33^ The interplay of NHEJ and MMEJ with non‐homologous sequences can induce chromosomal rearrangements.^34^ Halting the replication fork and separating the lagging strand from the original template causes it to change its physical proximity to another fork, and DNA synthesis begins again, resulting in the creation of the FoSTeS model.^35^ The MMBIR pathway, a repeat‐based mechanism, can cause complex chromosomal rearrangements through recombination between repetitive sequences.^36^ TEs, DNA sequences capable of changing their position within the genome, also contribute to CNV formation.^37^ TE can replicate independently within the genome of the host cell's DNA and relocate^37^, ^38^, ^39^ (Figure 1). The only currently active autonomous transposons in the human genome are L1 retrotransposition elements.^40^ Approximately 17% of the human genome comprises of L1 elements.^41^ The L1 element consists of a 5′ UTR (an internal RNA polymerase II promoter), two open reading frames (ORF1, coding for an RNA‐binding protein, and ORF2, encoding a protein with endonuclease (EN)) and a 3′ UTR (composed of a polyadenylation signal ending with an oligo(dA)‐rich tail of variable length) reverse transcriptase (RT) activity.^41^, ^42^ The transposition of L1 occurs via an RNA intermediate, likely integrating into the genome through targeted primary reverse transcription (TPRT),^41^ duplicate target sites (TSDs) being characteristic of the TPRT process.^41^

## 4 CNVS AND CARDIOVASCULAR DISORDERS

**FIGURE 2:**
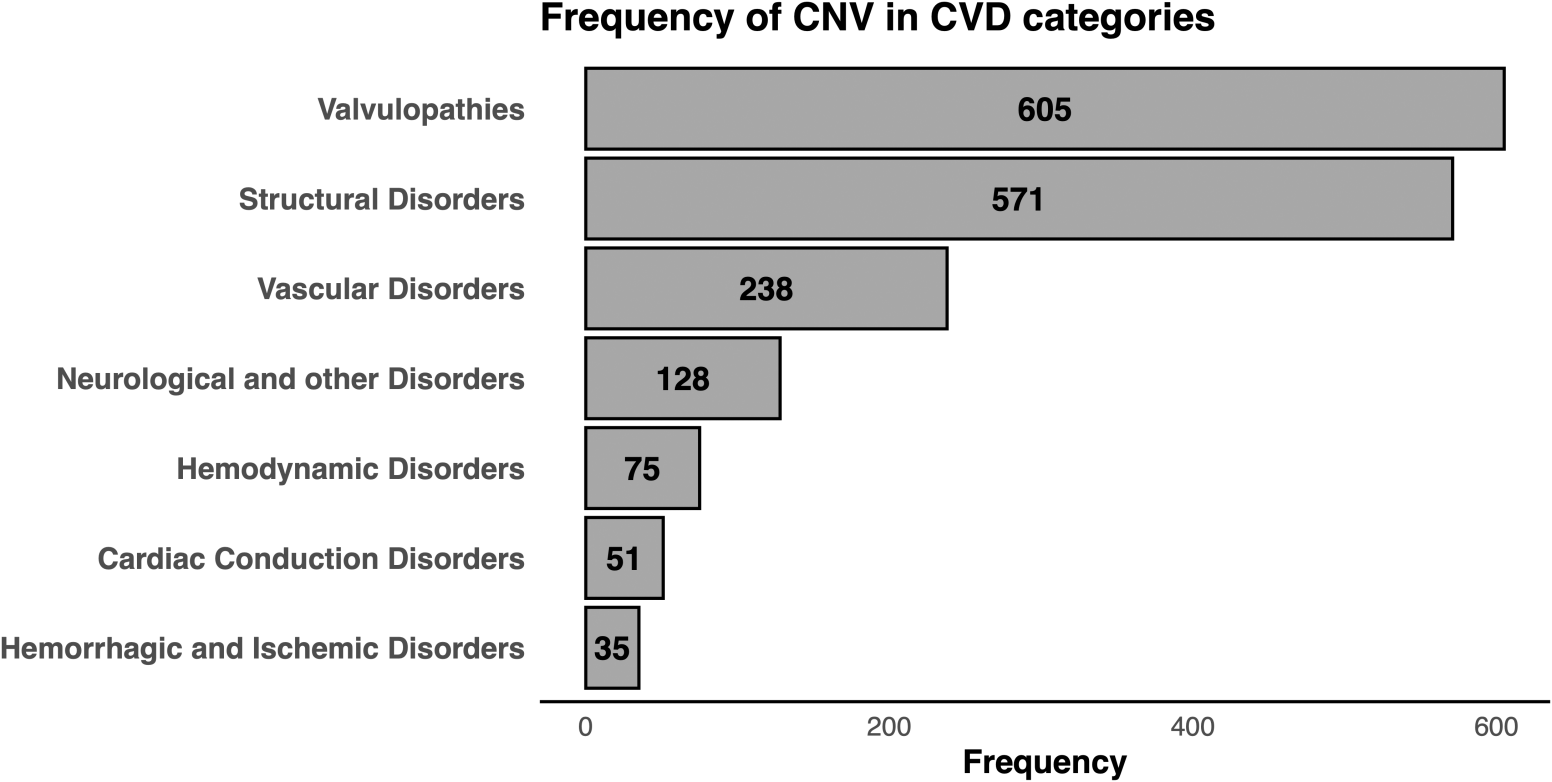
Frequency of reported CNVs for each CVD category.

While CVDs have been extensively studied from various perspectives, including the origin of the disorder, influential factors and genetic variations, there has been limited research on the connection between CVDs and CNVs. One study observed CNVs in 0.3%–11% of individuals with hereditary heart disease.^43^ Additionally, a genome‐wide analysis of low‐frequency CNVs identified 11 significant new genomic associations with major cardiovascular disease traits.^44^ Reported CNVs in CVDs are detailed in Table 1 and Figure 2. Table 1 summarizes the pathogenicity of each variant in accordance with ACMG guidelines, as sourced from the HGMD database (https://www.hgmd.cf.ac.uk/ac/index.php).^45^ Therefore, the pathogenicity of these variants is determined based on the guidelines from the year of publication. Most CNVs associated with cardiovascular diseases have been detected in patients with cardiac valvulopathies, transposition of great arteries (TGA), patent foramen ovale (PFO), secundum atrial septal defect, cardiomegaly, hypertension, cutis marmorata or hypoplastic left heart. The distribution of reported CNVs across these CVDs is illustrated in Figure 2. Approximately 10% of the reported CNVs with at least VUS pathogenicity were detected in patients with cardiac valvulopathies.

**TABLE 1:** All reported CNVs (pathogenic, likely pathogenic and variant of uncertain significance).

No.	CNV interval	Chromosomal location (hg38)	Chromosome	CNV type	Disease	Zygosity	Pathogenicity	Size	Number of genes
1	17:36461608–40389320	q12‐q21.2	17	Duplication	Holosystolic murmur	Heterozygote	Pathogenic	3.93 MB	105
2	7:36464010–44501375	p14.2‐p13	7	Deletion	Aortic valve stenosis	Heterozygote	VUS	8.04 MB	107
3	2:60846545–62662113	p16.1‐p15	2	Deletion	Aortic valve stenosis	Heterozygote	VUS	1.82 MB	34
4	2:142877664–147177434	q22.2‐q22.3	2	Deletion	Aortic valve stenosis	Heterozygote	VUS	4.30 MB	28
5	16:83504564–83636810	q23.3	16	Deletion	Aortic valve stenosis	Heterozygote	VUS	132.25 KB	2
6	5:87009045–90397059	q14.3	5	Deletion	Aortic valve stenosis	Heterozygote	VUS	3.39 MB	26
7	7:120276669–134687108	q31.31‐q33	7	Duplication	Aortic valve stenosis	Heterozygote	VUS	14.41 MB	158
8	2:34048–21148196	p25.3‐p24.1	2	Duplication	Aortic valve stenosis	Heterozygote	VUS	21.11 MB	178
9	18:220070–1224563	p11.32	18	Deletion	Aortic valve stenosis	Heterozygote	VUS	1.00 MB	17
10	3:59717083–61035866	p14.2	3	Duplication	Aortic valve stenosis	Heterozygote	VUS	1.32 MB	5
11	10:123496700–133769367	q26.13‐q26.3	10	Duplication	Aortic valve stenosis	Heterozygote	VUS	10.27 MB	124
12	11:127562910–135074876	q24.2‐q25	11	Deletion	Aortic valve stenosis	Heterozygote	VUS	7.51 MB	69
13	Y:382794–957140	p11.31‐p11.2	Y	Duplication	Aortic valve stenosis	Heterozygote	VUS	574.35 KB	4
14	8:11573697–11948311	p23.1	8	Duplication	Aortic valve stenosis	Heterozygote	VUS	374.62 KB	8
15	6:45497175–46028585	p21.1	6	Duplication	Aortic valve stenosis	Heterozygote	VUS	531.41 KB	4
16	2:139962009–144836022	q22.1‐q22.3	2	Deletion	Aortic valve stenosis	Heterozygote	VUS	4.87 MB	33
17	8:8273108–11984392	p23.1	8	Deletion	Aortic valve stenosis	Heterozygote	VUS	3.71 MB	57
18	7:12050374–13821375	q21.3‐q21.2	7	Duplication	Aortic valve stenosis	Heterozygote	VUS	1.77 MB	8
19	22:48241404–50806138	q13.32‐q13.33	22	Deletion	Aortic valve stenosis	Heterozygote	VUS	2.56 MB	54
20	17:7496685–8030459	p13.1	17	Deletion	Aortic valve stenosis	Heterozygote	VUS	533.77 KB	33
21	11:118519419–118521531	q23.3	11	Deletion	Aortic valve stenosis	Heterozygote	VUS	2.11 KB	2
22	4:42412739–42524827	p13	4	Duplication	Aortic valve stenosis	Heterozygote	VUS	112.09 KB	2
23	3:163217321–166682158	q26.1	3	Duplication	Aortic valve stenosis	Heterozygote	VUS	3.46 MB	17
24	15:74849186–99742814	q24.1‐q26.3	15	Duplication	Aortic valve stenosis	Heterozygote	Pathogenic	24.89 MB	352
25	10:132116268–133409120	q26.3	10	Deletion	Aortic valve stenosis	Heterozygote	Pathogenic	1.29 MB	34
26	10:26729206–28746254	p12.1	10	Deletion	Aortic valve stenosis	Heterozygote	Pathogenic	2.02 MB	41
27	7:73976261–74031476	q11.23	7	Duplication	Aortic valve stenosis	Heterozygote	VUS	55.22 KB	1
28	7:73307413–74728004	q11.23	7	Deletion	Aortic valve stenosis	Heterozygote	VUS	1.42 MB	35
29	14:76887174–76995369	q24.3	14	Duplication	Aortic valve stenosis	Heterozygote	VUS	108.20 KB	3
30	14:77685856–77932925	q24.3	14	Duplication	Aortic valve stenosis	Heterozygote	VUS	247.07 KB	6
31	8:107436509–120735030	q23.1‐q24.12	8	Deletion	Aortic valve stenosis	Heterozygote	Pathogenic	13.30 MB	74
32	15:77492439–77828905	q24.3	15	Duplication	Aortic valve stenosis	Heterozygote	VUS	336.47 KB	3
33	4:169902854–177580762	q33‐q34.3	4	Deletion	Aortic valve stenosis	Heterozygote	Pathogenic	7.68 MB	53
34	8:11465771–11987815	p23.1	8	Duplication	Aortic valve stenosis	Heterozygote	VUS	522.04 KB	12
35	1:45451454–46630224	p34.1‐p33	1	Duplication	Aortic valve stenosis	Heterozygote	VUS	1.18 MB	35
36	5:37188310–37518831	p13.2	5	Duplication	Aortic valve stenosis	Heterozygote	VUS	330.52 KB	8
37	4:169504119–190107927	q33‐q35.2	4	Deletion	Aortic valve stenosis	Heterozygote	Likely pathogenic	20.60 MB	176
38	10:116650499–133334830	q25.3‐q26.3	10	Deletion	Aortic valve stenosis	Heterozygote	Likely pathogenic	16.68 MB	180
39	11:110624066–135076622	q23.1‐q25	11	Deletion	Aortic valve stenosis	Heterozygote	Likely pathogenic	24.45 MB	412
40	21:30205811–46699983	q22.11‐q22.3	21	Deletion	Aortic valve stenosis	Heterozygote	Likely pathogenic	16.49 MB	356
41	11:115124070–135076622	q23.3‐q25	11	Deletion	Aortic valve stenosis	Heterozygote	Likely pathogenic	19.95 MB	330
42	5:10001–18464134	p15.33‐p14.3	5	Deletion	Aortic valve stenosis	Heterozygote	Likely pathogenic	18.45 MB	186
43	7:138458000–159253352	q33‐q36.3	7	Deletion	Aortic valve stenosis	Heterozygote	Likely pathogenic	20.80 MB	391
44	18:63981786–80256282	q22.1‐q23	18	Deletion	Aortic valve stenosis	Heterozygote	Likely pathogenic	16.27 MB	96
45	9:10001–14110001	p24.3‐p23	9	Deletion	Aortic valve stenosis	Heterozygote	Likely pathogenic	14.10 MB	124
46	4:171304119–190107927	q34.1‐q35.2	4	Deletion	Aortic valve stenosis	Heterozygote	Likely pathogenic	18.80 MB	160
47	1:243510658–248938897	q44	1	Deletion	Aortic valve stenosis	Heterozygote	Likely pathogenic	5.43 MB	124
48	6:60001–15191790	p25.3‐p23	6	Deletion	Aortic valve stenosis	Heterozygote	Likely pathogenic	15.13 MB	149
49	16:57908595–76508602	q21‐q23.1	16	Deletion	Aortic valve stenosis	Heterozygote	Likely pathogenic	18.60 MB	272
50	17:150208–11055958	p13.3‐p12	17	Duplication	Aortic valve stenosis	Heterozygote	Likely pathogenic	10.91 MB	332
51	16:66508596–83208894	q21‐q23.3	16	Deletion	Aortic valve stenosis	Heterozygote	Likely pathogenic	16.70 MB	274
52	18:75459057–80256282	q23	18	Deletion	Aortic valve stenosis	Heterozygote	Likely pathogenic	4.80 MB	36
53	17:75992325–83101964	q25.1‐q25.3	17	Duplication	Aortic valve stenosis	Heterozygote	Likely pathogenic	7.11 MB	200
54	4:182603698–190107927	q35.1‐q35.2	4	Deletion	Aortic valve stenosis	Heterozygote	Likely pathogenic	7.50 MB	106
55	6:117872144–130937167	q22.1‐q22.2	6	Deletion	Aortic valve stenosis	Heterozygote	Likely pathogenic	13.07 MB	85
56	9:10001–9010000	p24.3‐p23	9	Duplication	Aortic valve stenosis	Heterozygote	Likely pathogenic	9.00 MB	105
57	8:60001–12798120	p23.3‐p23.1	8	Deletion	Aortic valve stenosis	Heterozygote	Likely pathogenic	12.74 MB	214
58	20:79360–11971352	p13‐p12.2	20	Duplication	Aortic valve stenosis	Heterozygote	Likely pathogenic	11.89 MB	180
59	15:81420604–101898930	q25.2‐q26.3	15	Deletion	Aortic valve stenosis	Heterozygote	Likely pathogenic	20.48 MB	273
60	6:60001–10491781	p25.3‐p24.3	6	Deletion	Aortic valve stenosis	Heterozygote	Likely pathogenic	10.43 MB	103
61	22:17446408–25579479	q11.21‐q12.1	22	Deletion	Aortic valve stenosis	Heterozygote	Likely pathogenic	8.13 MB	332
62	5:93038538–115936404	q15‐q23.1	5	Deletion	Aortic valve stenosis	Heterozygote	Likely pathogenic	22.90 MB	177
63	15:77920603–101898930	q24.3‐q26.3	15	Duplication	Aortic valve stenosis	Heterozygote	Likely pathogenic	23.98 MB	331
64	13:108549652–113340664	q33.3‐q34	13	Deletion	Aortic valve stenosis	Heterozygote	Likely pathogenic	4.79 MB	61
65	7:61071607–77633657	q11.1‐q11.23	7	Deletion	Aortic valve stenosis	Heterozygote	Likely pathogenic	16.56 MB	252
66	22:16367189–25579479	q11.1‐q12.1	22	Deletion	Aortic valve stenosis	Heterozygote	Likely pathogenic	9.21 MB	372
67	17:150208–22763679	p13.3‐p11.1	17	Duplication	Aortic valve stenosis	Heterozygote	Likely pathogenic	22.61 MB	587
68	18:56681771–80256282	q21.31‐q23	18	Deletion	Aortic valve stenosis	Heterozygote	Likely pathogenic	23.57 MB	179
69	9:14210374–16338034	p22.3	9	Deletion	Aortic valve stenosis	Heterozygote	Likely pathogenic	2.13 MB	23
70	16:14991897–18170481	p13.11‐p12.3	16	Duplication	Aortic valve stenosis	Heterozygote	Likely pathogenic	3.18 MB	35
71	7:74060528–74089583	q11.23	7	Deletion	Aortic valve stenosis	Heterozygote	Likely pathogenic	29.06 KB	3
72	4:112955366–130746647	q25‐q28.3	4	Deletion	Aortic valve stenosis	Heterozygote	Likely pathogenic	17.79 MB	128
73	22:18802558–21109971	q11.21	22	Duplication	Aortic valve stenosis	Heterozygote	Likely pathogenic	2.31 MB	89
74	7:40264549–40340443	p14.1	7	Deletion	Aortic valve stenosis	Heterozygote	VUS	75.89 KB	1
75	X:154208334–154607861	q28	X	Duplication	Aortic valve stenosis	Hemizygous	Pathogenic	399.53 KB	28
76	22:19036311–21207225	q11.21	22	Duplication	Aortic valve stenosis	Heterozygote	Likely pathogenic	2.17 MB	84
77	6:1610106–1617092	p25.3	6	Deletion	Aortic valve stenosis	Heterozygote	Likely pathogenic	6.99 KB	1
78	6:107754550–110102404	q21	6	Duplication	Supravalvular aortic stenosis	Heterozygote	VUS	2.35 MB	39
79	7:73312522–74725117	q11.23	7	Deletion	Supravalvular aortic stenosis	Heterozygote	VUS	1.41 MB	34
80	7:72867585–74366074	q11.23	7	Deletion	Supravalvular aortic stenosis	Heterozygote	Pathogenic	1.50 MB	49
81	7:73942884–74031476	q11.23	7	Duplication	Supravalvular aortic stenosis	Heterozygote	VUS	88.59 KB	1
82	X:2782275–57905840	p22.33‐p11.21	X	Deletion	Supravalvular aortic stenosis	Unknown	VUS	55.12 MB	650
83	X:58025272–155611794	p11.21‐q28	X	Copy number gain	Supravalvular aortic stenosis	Unknown	VUS	97.59 MB	1175
84	2:130206881–130387092	q21.1	2	Duplication	Supravalvular aortic stenosis	Heterozygote	VUS	180.21 KB	19
85	7:73976261–74031476	q11.23	7	Duplication	Supravalvular aortic stenosis	Heterozygote	Likely pathogenic	55.22 KB	1
86	7:73736181–73991230	q11.23	7	Deletion	Supravalvular aortic stenosis	Heterozygote	Likely pathogenic	255.05 KB	5
87	7:73304280–74727155	q11.23	7	Deletion	Supravalvular aortic stenosis	Heterozygote	Pathogenic	1.42 MB	35
88	7:73287279–74732517	q11.23	7	Deletion	Supravalvular aortic stenosis	Heterozygote	Pathogenic	1.45 MB	36
89	7:73197914–74345185	q11.23	7	Deletion	Supravalvular aortic stenosis	Heterozygote	VUS	1.15 MB	35
90	11:35159532–45058203	p13‐p11.2	11	Duplication	Supravalvular aortic stenosis	Heterozygote	Pathogenic	9.90 MB	58
91	X:19593628–19929980	p22.12	X	Duplication	Supravalvular aortic stenosis	Heterozygote	VUS	336.35 KB	2
92	15:31729530–32146742	q13.3	15	Deletion	Supravalvular aortic stenosis	Heterozygote	VUS	417.21 KB	3
93	7:74031409–74110950	q11.23	7	Deletion	Supravalvular aortic stenosis	Heterozygote	Pathogenic	79.54 KB	3
94	7:73755813–74719013	q11.23	7	Deletion	Supravalvular aortic stenosis	Heterozygote	Pathogenic	963.20 KB	16
95	7:73308984–74169373	q11.23	7	Deletion	Supravalvular aortic stenosis	Heterozygote	Pathogenic	860.39 KB	25
96	7:72382983–73976980	q11.22‐q11.23	7	Deletion	Supravalvular aortic stenosis	Heterozygote	Likely pathogenic	1.59 MB	47
97	17:13367560–16874788	p12‐p11.2	17	Deletion	Aortic regurgitation	Heterozygote	VUS	3.51 MB	75
98	16:21385454–22761035	p12.2	16	Deletion	Aortic regurgitation	Heterozygote	VUS	1.38 MB	29
99	14:102553910–106880765	q32.31‐q32.33	14	Deletion	Aortic regurgitation	Heterozygote	VUS	4.33 MB	278
100	12:10801653–12079392	p13.2	12	Deletion	Aortic regurgitation	Heterozygote	VUS	1.28 MB	31
101	8:12838024–13059710	p22	8	Duplication	Aortic regurgitation	Heterozygote	VUS	221.69 KB	2
102	22:18921519–20951827	q11.21	22	Deletion	Aortic regurgitation	Heterozygote	VUS	2.03 MB	72
103	7:163010–3174614	p22.3‐p22.2	7	Deletion	Aortic regurgitation	Heterozygote	VUS	3.01 MB	51
104	X:10522949–10523299	p22.2	X	Duplication	Aortic regurgitation	Hemizygous	Pathogenic	351 BP	1
105	16:14991897–16188450	p13.11	16	Duplication	Aortic regurgitation	Heterozygote	VUS	1.20 MB	22
106	16:29584162–29827124	p11.2	16	Duplication	Aortic regurgitation	Heterozygote	VUS	242.96 KB	15
107	16:29861040–30007179	p11.2	16	Duplication	Aortic regurgitation	Heterozygote	VUS	146.14 KB	11
108	16:30085268–30181240	p11.2	16	Duplication	Aortic regurgitation	Heterozygote	VUS	95.97 KB	6
109	4:35672405–35716298	p15.1	4	Deletion	Aortic regurgitation	Heterozygote	VUS	43.89 KB	0
110	14:51787673–51790976	q22.1	14	Deletion	Aortic regurgitation	Heterozygote	VUS	3.30 KB	0
111	5:56016213–56038049	q11.2	5	Deletion	Aortic regurgitation	Heterozygote	VUS	21.84 KB	0
112	20:59436575–59446328	q13.32	20	Deletion	Aortic regurgitation	Heterozygote	VUS	9.75 KB	0
113	10:26844085–37604324	p12.1‐p11.21	10	Duplication	Aortic regurgitation	Heterozygote	Pathogenic	10.76 MB	141
114	7:148165260–159253352	q35‐q36.3	7	Deletion	Aortic regurgitation	Heterozygote	Likely pathogenic	11.09 MB	147
115	5:169040417–181478259	q35.1‐q35.3	5	Deletion	Aortic regurgitation	Heterozygote	Likely pathogenic	12.44 MB	219
116	16:10001–16698642	p13.3‐p13.11	16	Duplication	Aortic regurgitation	Heterozygote	Likely pathogenic	16.69 MB	396
117	10:128511746–133334830	q26.2‐q26.3	10	Deletion	Aortic regurgitation	Heterozygote	Likely pathogenic	4.82 MB	44
118	2:230509781–242160331	q37.1‐q37.3	2	Deletion	Aortic regurgitation	Heterozygote	Likely pathogenic	11.65 MB	211
119	10:116650499–133334830	q25.3‐q26.3	10	Deletion	Aortic regurgitation	Heterozygote	Likely pathogenic	16.68 MB	180
120	8:60001–12798120	p23.3‐p23.1	8	Deletion	Aortic regurgitation	Heterozygote	Likely pathogenic	12.74 MB	214
121	6:60001–10491781	p25.3‐p24.3	6	Deletion	Aortic regurgitation	Heterozygote	Likely pathogenic	10.43 MB	103
122	4:112955366–130746647	q25‐q28.3	4	Deletion	Aortic regurgitation	Heterozygote	Likely pathogenic	17.79 MB	128
123	9:10001–19910002	p24.3‐p21.3	9	Deletion	Aortic regurgitation	Heterozygote	Likely pathogenic	19.90 MB	173
124	12:85805266–88269414	q21.31‐q21.32	12	Duplication	Aortic regurgitation	Heterozygote	Likely pathogenic	2.46 MB	13
125	6:139751940–151251065	q24.31‐q25.1	6	Deletion	Aortic regurgitation	Heterozygote	VUS	11.50 MB	101
126	X:7852595–8457596	p22.31	X	Duplication	Tricuspid stenosis	Heterozygote	VUS	605.00 KB	3
127	3:187669587–196872267	q27.3‐q29	3	Deletion	Tricuspid stenosis	Heterozygote	VUS	9.20 MB	131
128	1:145635445–146046645	q21.1	1	Duplication	Tricuspid stenosis	Heterozygote	VUS	411.20 KB	19
129	17:237158–2012818	p13.3	17	Deletion	Tricuspid stenosis	Heterozygote	VUS	1.78 MB	39
130	13:22992763–24392307	q12.12	13	Deletion	Tricuspid stenosis	Heterozygote	VUS	1.40 MB	26
131	3:18323–16383489	p26.3‐p24.3	3	Deletion	Tricuspid stenosis	Heterozygote	Likely pathogenic	16.37 MB	191
132	X:137892321–154900412	q26.3‐q28	X	Deletion	Tricuspid stenosis	Heterozygote	Likely pathogenic	17.01 MB	265
133	17:150208–3449956	p13.3‐p13.2	17	Deletion	Tricuspid stenosis	Heterozygote	Likely pathogenic	3.30 MB	88
134	12:120199477–132891443	q24.23‐q24.33	12	Duplication	Tricuspid stenosis	Heterozygote	VUS	12.69 MB	201
135	17:150208–11055958	p13.2‐p12	17	Deletion	Tricuspid stenosis	Heterozygote	Likely pathogenic	10.91 MB	332
136	7:138458000–159253352	q33‐q36.3	7	Duplication	Tricuspid stenosis	Heterozygote	Likely pathogenic	20.80 MB	391
137	9:10001–9010000	p24.3‐p23	9	Deletion	Tricuspid stenosis	Heterozygote	Likely pathogenic	9.00 MB	105
138	22:16367189–37152953	q11.1‐q12.3	22	Deletion	Tricuspid stenosis	Heterozygote	Likely pathogenic	20.79 MB	583
139	8:60001–12798120	p23.3‐p23.1	8	Deletion	Tricuspid stenosis	Heterozygote	Likely pathogenic	12.74 MB	214
140	1:914086–2516854	p36.33‐p36.32	1	Deletion	Tricuspid stenosis	Heterozygote	Likely pathogenic	1.60 MB	77
141	9:9345062–16124092	p23‐p22.3	9	Deletion	Tricuspid regurgitation	Heterozygote	VUS	6.78 MB	41
142	2:48991894–49115665	p16.3	2	Deletion	Tricuspid regurgitation	Heterozygote	VUS	123.77 KB	2
143	9:220253–304644	p24.3	9	Duplication	Tricuspid regurgitation	Heterozygote	VUS	84.39 KB	1
144	3:181780965–183363767	q26.33‐q27.1	3	Deletion	Tricuspid regurgitation	Heterozygote	VUS	1.58 MB	19
145	10:109723412–111915979	q25.1‐q25.2	10	Deletion	Tricuspid regurgitation	Heterozygote	VUS	2.19 MB	26
146	12:132449561–132807336	q24.33	12	Duplication	Tricuspid regurgitation	Heterozygote	VUS	357.78 KB	10
147	21:32481897–32686896	q22.11	21	Duplication	Tricuspid regurgitation	Heterozygote	VUS	205.00 KB	8
148	18:62363921–80256699	q21.33‐q23	18	Deletion	Tricuspid regurgitation	Heterozygote	VUS	17.89 MB	118
149	12:80412–34025274	p13.33‐p11.1	12	Duplication	Tricuspid regurgitation	Heterozygote	VUS	33.94 MB	509
150	9:137783226–138076863	q34.3	9	Deletion	Tricuspid regurgitation	Heterozygote	VUS	293.64 KB	3
151	17:36486499–38203073	q12	17	Duplication	Tricuspid regurgitation	Heterozygote	VUS	1.72 MB	30
152	12:45001–38059419	p13.33‐q12	12	Duplication	Tricuspid regurgitation	Heterozygote	VUS	38.01 MB	515
153	22:24734343–40686039	q11.23‐q13.2	22	Duplication	Tricuspid regurgitation	Heterozygote	Pathogenic	15.95 MB	323
154	9:220253–138212068	p24.3‐q34.3	9	Duplication	Tricuspid regurgitation	Heterozygote	Pathogenic	137.99 MB	1553
155	16:46407633–62056029	q11.2‐q21	16	Duplication	Tricuspid regurgitation	Heterozygote	Pathogenic	15.65 MB	199
156	16:15398460–18068310	p13.11‐p12.3	16	Deletion	Tricuspid regurgitation	Heterozygote	VUS	2.67 MB	26
157	11:110624066–135076622	q23.1‐q25	11	Duplication	Tricuspid regurgitation	Heterozygote	Likely pathogenic	24.45 MB	412
158	18:10001–15410899	p11.32‐p11.1	18	Deletion	Tricuspid regurgitation	Heterozygote	Likely pathogenic	15.40 MB	200
159	17:150208–3449956	p13.3‐p13.2	17	Deletion	Tricuspid regurgitation	Heterozygote	Likely pathogenic	3.30 MB	88
160	22:43779219–50668400	q13.2‐q13.33	22	Deletion	Tricuspid regurgitation	Heterozygote	Likely pathogenic	6.89 MB	113
161	7:10239–13687135	p22.3‐p21.3	7	Duplication	Tricuspid regurgitation	Heterozygote	Likely pathogenic	13.68 MB	150
162	7:7087129–16387135	p22.1‐p21.2	7	Triplication	Tricuspid regurgitation	Heterozygote	Likely pathogenic	9.30 MB	43
163	7:13287136–30487144	p21.3‐p14.3	7	Duplication	Tricuspid regurgitation	Heterozygote	Likely pathogenic	17.20 MB	173
164	12:45001–12855799	p13.33‐p13.1	12	Duplication	Tricuspid regurgitation	Heterozygote	Likely pathogenic	12.81 MB	303
165	10:75870236–97250253	q22.2‐q24.1	10	Deletion	Tricuspid regurgitation	Heterozygote	Likely pathogenic	21.38 MB	280
166	7:148165260–159253352	q35‐q36.3	7	Deletion	Tricuspid regurgitation	Heterozygote	Likely pathogenic	11.09 MB	147
167	18:69081783–80256282	q22.1‐q23	18	Deletion	Tricuspid regurgitation	Heterozygote	Likely pathogenic	11.17 MB	73
168	4:10001–4380201	p16.3	4	Deletion	Tricuspid regurgitation	Heterozygote	Likely pathogenic	4.37 MB	94
169	18:75459057–80256282	q23	18	Deletion	Tricuspid regurgitation	Heterozygote	Likely pathogenic	4.80 MB	36
170	1:10001–6905674	p36.33‐p36.31	1	Deletion	Tricuspid regurgitation	Heterozygote	Likely pathogenic	6.90 MB	177
171	10:116650499–133334830	q25.3‐q26.3	10	Deletion	Tricuspid regurgitation	Heterozygote	Likely pathogenic	16.68 MB	180
172	7:10239–7087129	p22.3‐p22.1	7	Deletion	Tricuspid regurgitation	Heterozygote	Likely pathogenic	7.08 MB	119
173	6:142137170–151986751	q24.1‐q25.1	6	Deletion	Tricuspid regurgitation	Heterozygote	Likely pathogenic	9.85 MB	101
174	11:115124071–135076622	q23.3‐q25	11	Deletion	Tricuspid regurgitation	Heterozygote	Likely pathogenic	19.95 MB	330
175	1:914087–4071190	P36.33‐p36.32	1	Deletion	Tricuspid regurgitation	Heterozygote	Likely pathogenic	3.16 MB	108
176	8:240569–11111416	p23.3‐p23.1	8	Deletion	Tricuspid regurgitation	Heterozygote	Likely pathogenic	10.87 MB	152
177	6:148511066–150947913	q25.1	6	Deletion	Tricuspid regurgitation	Heterozygote	VUS	2.44 MB	43
178	15:51314381–73572490	q21.2‐q24.1	15	Duplication	Mitral stenosis	Heterozygote	VUS	22.26 MB	282
179	6:45497175–46028585	P21.1	6	Duplication	Mitral stenosis	Heterozygote	VUS	531.41 KB	4
180	22:38495688–41586395	q13.1‐q13.2	22	Duplication	Mitral stenosis	Heterozygote	Likely pathogenic	3.09 MB	83
181	15:74849186–99742814	q24.1‐q26.3	15	Duplication	Mitral stenosis	Heterozygote	Pathogenic	24.89 MB	352
182	10:132116268–133409120	q26.3	10	Deletion	Mitral stenosis	Heterozygote	Pathogenic	1.29 MB	34
183	17:36459263–37890177	q12	17	Duplication	Mitral stenosis	Heterozygote	Likely pathogenic	1.43 MB	21
184	18:63981786–80256282	q22.1‐q23	18	Deletion	Mitral stenosis	Heterozygote	Likely pathogenic	16.27 MB	96
185	20:59591551–64334167	q13.32‐q13.33	20	Duplication	Mitral stenosis	Heterozygote	Likely pathogenic	4.74 MB	119
186	14:99763910–105773834	q32.2‐q32.33	14	Deletion	Mitral stenosis	Heterozygote	Likely pathogenic	6.01 MB	238
187	11:115124070–135076622	q23.3‐q25	11	Deletion	Mitral stenosis	Heterozygote	Likely pathogenic	19.95 MB	330
188	17:150208–11055958	p13.3‐p12	17	Deletion	Mitral stenosis	Heterozygote	Likely pathogenic	10.91 MB	332
189	8:60001–12798120	p23.3‐p23.1	8	Deletion	Mitral stenosis	Heterozygote	Likely pathogenic	12.74 MB	214
190	18:10001–15410899	p11.32‐p11.1	18	Triplication	Mitral stenosis	Heterozygote	Likely pathogenic	15.40 MB	200
191	17:150208–22763679	p13.3‐p11.1	17	Duplication	Mitral stenosis	Heterozygote	Likely pathogenic	22.61 MB	587
192	17:75992325–83101964	q25.1‐q25.3	17	Deletion	Mitral stenosis	Heterozygote	Likely pathogenic	7.11 MB	200
193	3:177127356–177842479	q26.32	3	Deletion	Mitral stenosis	Heterozygote	Pathogenic	715.12 KB	6
194	6:148592859–151224941	q25.1	6	Deletion	Mitral stenosis	Heterozygote	VUS	2.63 MB	43
195	22:19036311–21207225	q11.21	22	Duplication	Mitral stenosis	Heterozygote	Likely pathogenic	2.17 MB	84
196	8:8134092–11908200	p23.1	8	Deletion	Mitral regurgitation	Heterozygote	VUS	3.77 MB	57
197	5:204734–6753840	p15.33‐p15.31	5	Triplication	Mitral regurgitation	Heterozygote	VUS	6.55 MB	71
198	5:7521125–43644823	p15.31‐p12	5	Duplication	Mitral regurgitation	Heterozygote	VUS	36.12 MB	292
199	6:41232978–43640764	p21.1	6	Deletion	Mitral regurgitation	Heterozygote	VUS	2.41 MB	77
200	16:89409042–89585740	q24.3	16	Deletion	Mitral regurgitation	Heterozygote	VUS	176.70 KB	6
201	5:175780161–178602140	q35.2‐q35.3	5	Deletion	Mitral regurgitation	Heterozygote	VUS	2.82 MB	64
202	8:8594877–11908210	p23.1	8	Deletion	Mitral regurgitation	Heterozygote	VUS	3.31 MB	49
203	1:150214890–164424856	q21.2‐q23.3	1	Duplication	Mitral regurgitation	Heterozygote	VUS	14.21 MB	500
204	3:187460995–191620178	q27.3‐q28	3	Deletion	Mitral regurgitation	Heterozygote	VUS	4.16 MB	33
205	3:138494233–139106215	q22.3‐q23	3	Duplication	Mitral regurgitation	Heterozygote	VUS	611.98 KB	17
206	X:86546862–86579991	q21.2	X	Duplication	Mitral regurgitation	Hemizygous	VUS	33.13 KB	1
207	2:177685217–177712631	q31.2	2	Duplication	Mitral regurgitation	Heterozygote	VUS	27.41 KB	2
208	21:32481897–32686896	q22.11	21	Duplication	Mitral regurgitation	Heterozygote	VUS	205.00 KB	8
209	12:70161879–72759411	q15‐q21.1	12	Deletion	Mitral regurgitation	Heterozygote	VUS	2.60 MB	21
210	15:47387244–49255615	q21.1‐q21.2	15	Deletion	Mitral regurgitation	Heterozygote	VUS	1.87 MB	23
211	6:70667344–75932380	q13‐q14.1	6	Duplication	Mitral regurgitation	Heterozygote	VUS	5.27 MB	67
212	17:45625886–46130946	q21.31	17	Deletion	Mitral regurgitation	Heterozygote	Pathogenic	505.06 KB	9
213	12:45001–38059419	p13.33‐q12	12	Duplication	Mitral regurgitation	Heterozygote	Pathogenic	38.01 MB	515
214	22:38495688–41586395	q13.1‐q13.2	22	Duplication	Mitral regurgitation	Heterozygote	Likely pathogenic	3.09 MB	83
215	14:103179169–105795459	q32.32‐q32.33	14	Deletion	Mitral regurgitation	Heterozygote	Likely pathogenic	2.62 MB	85
216	17:45640337–46082496	q21.31	17	Deletion	Mitral regurgitation	Heterozygote	Pathogenic	442.16 KB	9
217	10:26844085–37604324	p12.1‐p11.21	10	Duplication	Mitral regurgitation	Heterozygote	Pathogenic	10.76 MB	141
218	2:168873339–169042705	q24.3‐q31.1	2	Deletion	Mitral regurgitation	Heterozygote	Pathogenic	169.37 KB	3
219	1:8365461–10720627	p36.23‐p36.22	1	Deletion	Mitral regurgitation	Heterozygote	Pathogenic	2.36 MB	50
220	15:30662946–32223480	q13.2‐q13.3	15	Duplication	Mitral regurgitation	Heterozygote	Likely pathogenic	1.56 MB	15
221	1:119998167–120081455	p12	1	Deletion	Mitral regurgitation	Heterozygote	VUS	83.29 KB	1
222	8:116518581–126118574	q23.3‐q24.13	8	Deletion	Mitral regurgitation	Heterozygote	Likely pathogenic	9.60 MB	99
223	6:60001–7054768	p25.3‐p25.1	6	Deletion	Mitral regurgitation	Heterozygote	Likely pathogenic	6.99 MB	76
224	6:164008557–170687856	q26‐q27	6	Deletion	Mitral regurgitation	Heterozygote	Likely pathogenic	6.68 MB	63
225	3:18323–16383489	p26.3‐p24.3	3	Deletion	Mitral regurgitation	Heterozygote	Likely pathogenic	16.37 MB	191
226	20:44337947–64334167	q13.12‐q13.33	20	Deletion	Mitral regurgitation	Heterozygote	Likely pathogenic	20.00 MB	373
227	8:27386566–39423324	p21.2‐p11.22	8	Deletion	Mitral regurgitation	Heterozygote	Likely pathogenic	12.04 MB	140
228	1:84122296–106345336	p31.1‐p21.1	1	Deletion	Mitral regurgitation	Heterozygote	Likely pathogenic	22.22 MB	238
229	9:10001–19910002	p24.3‐p21.3	9	Duplication	Mitral regurgitation	Heterozygote	Likely pathogenic	19.90 MB	173
230	9:125758167–131295059	q33.3‐q34.13	9	Deletion	Mitral regurgitation	Heterozygote	Likely pathogenic	5.54 MB	124
231	22:43779219–50668400	q13.2‐q13.33	22	Deletion	Mitral regurgitation	Heterozygote	Likely pathogenic	6.89 MB	113
232	18:75459057–80256282	q23	18	Deletion	Mitral regurgitation	Heterozygote	Likely pathogenic	4.80 MB	36
233	4:10001–11322107	p16.3‐p15.33	4	Duplication	Mitral regurgitation	Heterozygote	Likely pathogenic	11.31 MB	208
234	22:16367189–25579479	q11.1‐q12.1	22	Triplication	Mitral regurgitation	Heterozygote	Likely pathogenic	9.21 MB	372
235	9:10001–9010000	p24.3‐p23	9	Duplication	Mitral regurgitation	Heterozygote	Likely pathogenic	9.00 MB	105
236	7:138458000–159253352	q33‐q36.3	7	Deletion	Mitral regurgitation	Heterozygote	Likely pathogenic	20.80 MB	391
237	1:10001–3041036	p36.33‐p36.32	1	Deletion	Mitral regurgitation	Heterozygote	Likely pathogenic	3.03 MB	120
238	12:27134884–27634952	p11.23‐p11.22	12	Duplication	Mitral regurgitation	Heterozygote	Likely pathogenic	500.07 KB	6
239	18:39603398–39921166	q12.3	18	Duplication	Mitral regurgitation	Heterozygote	Likely pathogenic	317.77 KB	7
240	4:112955366–130746647	q25‐q28.3	4	Deletion	Mitral regurgitation	Heterozygote	Likely pathogenic	17.79 MB	128
241	6:139751940–151251065	q24.1‐q25.1	6	Deletion	Mitral regurgitation	Heterozygote	VUS	11.50 MB	101
242	6:144257126–152927913	q24.2‐q25.2	6	Deletion	Mitral regurgitation	Heterozygote	VUS	8.67 MB	86
243	6:148758327–151251017	q25.1	6	Deletion	Mitral regurgitation	Heterozygote	VUS	2.49 MB	43
244	11:110624066–135076622	q23.1‐q25	11	Duplication	Tricuspid regurgitation	Heterozygote	Likely pathogenic	24.45 MB	412
245	4:182603698–190107927	q35.1‐q35.2	4	Deletion	Tricuspid regurgitation	Heterozygote	Likely pathogenic	7.50 MB	106
246	18:10001–15410899	p11.32‐p11.1	18	Deletion	Tricuspid regurgitation	Heterozygote	Likely pathogenic	15.40 MB	200
247	22:43779219–50668400	q13.2‐q13.33	22	Deletion	Tricuspid regurgitation	Heterozygote	Likely pathogenic	6.89 MB	113
248	7:10239–13687135	p22.3‐p21.3	7	Duplication	Tricuspid regurgitation	Heterozygote	Likely pathogenic	13.68 MB	150
249	7:7087129–16387135	p22.1‐p21.2	7	Triplication	Tricuspid regurgitation	Heterozygote	Likely pathogenic	9.30 MB	43
250	7:13287136–30487144	p21.3‐p14.3	7	Duplication	Tricuspid regurgitation	Heterozygote	Likely pathogenic	17.20 MB	173
251	12:45001–12855799	p13.33‐p13.1	12	Duplication	Tricuspid regurgitation	Heterozygote	Likely pathogenic	12.81 MB	303
252	1:914087–4071190	p36.33‐p36.32	1	Deletion	Tricuspid regurgitation	Heterozygote	Likely pathogenic	3.16 MB	108
253	8:240569–11111416	p23.3‐p23.1	8	Deletion	Tricuspid regurgitation	Heterozygote	Likely pathogenic	10.87 MB	152
254	6:148511066–150947913	q25.1	6	Deletion	Tricuspid regurgitation	Heterozygote	VUS	2.44 MB	43
255	8:8261773–12034555	p23.1	8	Deletion	Pulmonic stenosis	Heterozygote	Pathogenic	3.77 MB	59
256	22:29116547–29825060	q12.1‐q12.2	22	Deletion	Pulmonic stenosis	Heterozygote	VUS	708.51 KB	23
257	11:94370665–105717330	q21‐q22.3	11	Duplication	Pulmonic stenosis	Heterozygote	VUS	11.35 MB	98
258	11:107921399–113388163	q22.3‐q23.2	11	Deletion	Pulmonic stenosis	Heterozygote	VUS	5.47 MB	82
259	11:114364506–134781383	q23.2‐q25	11	Duplication	Pulmonic stenosis	Heterozygote	VUS	20.42 MB	334
260	5:171636769–174626263	q35.1‐q35.2	5	Deletion	Pulmonic stenosis	Heterozygote	VUS	2.99 MB	38
261	17:179378–2271915	p13.3	17	Deletion	Pulmonic stenosis	Heterozygote	VUS	2.09 MB	51
262	14:25999244–27205947	q12	14	Duplication	Pulmonic stenosis	Heterozygote	VUS	1.21 MB	8
263	10:57576181–62525570	q21.1‐q21.2	10	Deletion	Pulmonic stenosis	Heterozygote	VUS	4.95 MB	36
264	16:74605350–74772567	q23.1	16	Deletion	Pulmonic stenosis	Heterozygote	VUS	167.22 KB	6
265	2:146117596–151421784	q22.3‐q23.3	2	Deletion	Pulmonic stenosis	Heterozygote	Pathogenic	5.30 MB	43
266	4:206841–3305911	p16.3	4	Deletion	Pulmonic stenosis	Heterozygote	VUS	3.10 MB	66
267	12:57013355–63042498	q13.3‐q14.2	12	Deletion	Pulmonic stenosis	Heterozygote	VUS	6.03 MB	81
268	8:90940996–94538343	q21.3‐q22.1	8	Deletion	Pulmonic stenosis	Heterozygote	VUS	3.60 MB	38
269	8:8134092–11908200	p23.1	8	Deletion	Pulmonic stenosis	Heterozygote	VUS	3.77 MB	57
270	10:101145362–101691786	q24.31‐q24.32	10	Deletion	Pulmonic stenosis	Heterozygote	VUS	546.42 KB	14
271	17:45664085–46120237	q21.31	17	Deletion	Pulmonic stenosis	Heterozygote	VUS	456.15 KB	8
272	3:77230739–78385693	p12.3	3	Deletion	Pulmonic stenosis	Heterozygote	VUS	1.15 MB	6
273	12:84122732–91003505	q21.31‐q21.33	12	Deletion	Pulmonic stenosis	Heterozygote	VUS	6.88 MB	41
274	4:137900712–148008610	q28.3‐q31.23	4	Deletion	Pulmonic stenosis	Heterozygote	VUS	10.11 MB	99
275	1:7752337–13382061	p36.23‐p36.21	1	Deletion	Pulmonic stenosis	Heterozygote	VUS	5.63 MB	154
276	4:164685279–165065394	q32.3	4	Duplication	Pulmonic stenosis	Heterozygote	VUS	380.12 KB	11
277	4:168682078–169070347	q32.3	4	Duplication	Pulmonic stenosis	Heterozygote	VUS	388.27 KB	7
278	8:8134092–14559274	p23.1‐p22	8	Deletion	Pulmonic stenosis	Heterozygote	VUS	6.43 MB	108
279	7:110578659–113007933	q31.1	7	Deletion	Pulmonic stenosis	Heterozygote	VUS	2.43 MB	18
280	21:26619253–35238678	q21.3‐q22.12	21	Deletion	Pulmonic stenosis	Heterozygote	VUS	8.62 MB	147
281	6:90484846–92458812	q15	6	Deletion	Pulmonic stenosis	Heterozygote	VUS	1.97 MB	8
282	1:6143345–15361533	p36.31‐p36.21	1	Deletion	Pulmonic stenosis	Heterozygote	VUS	9.22 MB	200
283	1:74996430–75150570	p31.1	1	Duplication	Pulmonic stenosis	Heterozygote	VUS	154.14 KB	1
284	14:68142602–70452299	q24.1‐q24.2	14	Deletion	Pulmonic stenosis	Heterozygote	VUS	2.31 MB	36
285	4:80703309–82889885	q21.21‐q21.22	4	Deletion	Pulmonic stenosis	Heterozygote	VUS	2.19 MB	24
286	7:99743411–110049506	q22.1‐q31.1	7	Deletion	Pulmonic stenosis	Heterozygote	VUS	10.31 MB	197
287	6:151514794–152068227	q25.1	6	Duplication	Pulmonic stenosis	Heterozygote	VUS	553.43 KB	3
288	8:11573697–11948311	p23.1	8	Duplication	Pulmonic stenosis	Heterozygote	VUS	374.62 KB	8
289	3:196055007–197607943	q29	3	Deletion	Pulmonic stenosis	Heterozygote	VUS	1.55 MB	43
290	11:127334657–128917701	q24.2‐q24.3	11	Duplication	Pulmonic stenosis	Heterozygote	VUS	1.58 MB	14
291	19:19143277–19326270	p13.11	19	Deletion	Pulmonic stenosis	Heterozygote	VUS	182.99 KB	11
292	18:46771950–48955824	q21.1	18	Deletion	Pulmonic stenosis	Heterozygote	VUS	2.18 MB	26
293	4:184671593–187364224	q35.1‐q35.2	4	Duplication	Pulmonic stenosis	Heterozygote	VUS	2.69 MB	38
294	11:84828513–85032827	q14.1	11	Deletion	Pulmonic stenosis	Heterozygote	VUS	204.31 KB	2
295	3:153821150–198235559	q25.2‐q29	3	Trisomy	Pulmonic stenosis	Mosaic	Pathogenic	44.41 MB	540
296	18:62363921–80256699	q21.33‐q23	18	Deletion	Pulmonic stenosis	Heterozygote	VUS	17.89 MB	118
297	16:14991897–16188450	p13.11	16	Duplication	Pulmonic stenosis	Heterozygote	VUS	1.20 MB	22
298	16:15427856–17710509	p13.11‐p12.3	16	Deletion	Pulmonic stenosis	Heterozygote	VUS	2.28 MB	24
299	15:31436716–39451488	q13.3‐q14	15	Deletion	Pulmonic stenosis	Heterozygote	VUS	8.01 MB	82
300	X:153981220–154604471	q28	X	Duplication	Pulmonic stenosis	Heterozygote	VUS	623.25 KB	35
301	16:12007578–14668412	p13.13‐p13.12	16	Deletion	Pulmonic stenosis	Heterozygote	VUS	2.66 MB	21
302	2:139962009–144836022	q22.1‐q22.3	2	Deletion	Pulmonic stenosis	Heterozygote	VUS	4.87 MB	33
303	10:102092528–102936341	q24.32	10	Uniparental Isodisomy	Pulmonic stenosis	Heterozygote	VUS	843.81 KB	32
304	18:3100146–3344863	p11.31	18	Triplication	Pulmonic stenosis	Heterozygote	VUS	244.72 KB	4
305	X:6489363–7902394	p22.31	X	Deletion	Pulmonic stenosis	Heterozygote	Pathogenic	1.41 MB	7
306	7:73352334–74718984	q11.23	7	Deletion	Pulmonic stenosis	Heterozygote	VUS	1.37 MB	31
307	2:212242453–212305630	q34	2	Deletion	Pulmonic stenosis	Heterozygote	VUS	63.18 KB	1
308	17:237158–2012818	p13.3	17	Deletion	Pulmonic stenosis	Heterozygote	VUS	1.78 MB	39
309	13:22992763–24392307	q12.12	13	Deletion	Pulmonic stenosis	Heterozygote	VUS	1.40 MB	26
310	8:7896002–11984392	p23.1	8	Deletion	Pulmonic stenosis	Heterozygote	VUS	4.09 MB	75
311	8:8242662–12048898	p23.1	8	Deletion	Pulmonic stenosis	Heterozygote	VUS	3.81 MB	60
312	X:5779569–6324910	p22.32‐p22.31	X	Deletion	Pulmonic stenosis	Heterozygote	VUS	545.34 KB	1
313	6:366332–771504	p25.3	6	Deletion	Pulmonic stenosis	Heterozygote	VUS	405.17 KB	3
314	22:16413886–20208946	q11.1‐q11.21	22	Deletion	Pulmonic stenosis	Heterozygote	VUS	3.80 MB	125
315	17:7412738–7750866	p13.1	17	Duplication	Pulmonic stenosis	Heterozygote	VUS	338.13 KB	30
316	3:11351615–11576994	p25.3	3	Deletion	Pulmonic stenosis	Heterozygote	VUS	225.38 KB	2
317	4:70295481–70415837	q13.3	4	Deletion	Pulmonic stenosis	Heterozygote	VUS	120.36 KB	4
318	1:246911039–248878730	q44	1	Duplication	Pulmonic stenosis	Heterozygote	VUS	1.97 MB	95
319	9:137775065–137782613	q34.3	9	Deletion	Pulmonic stenosis	Heterozygote	Likely pathogenic	7.55 KB	1
320	15:101504247–101770451	q26.3	15	Duplication	Pulmonic stenosis	Heterozygote	VUS	266.20 KB	7
321	6:97186220–112715805	q16.1‐q21	6	Deletion	Pulmonic stenosis	Heterozygote	VUS	15.53 MB	136
322	19:36065923–36076834	q13.12	19	Deletion	Pulmonic stenosis	Heterozygote	Likely pathogenic	10.91 KB	1
323	1:244968795–245502219	q44	1	Duplication	Pulmonic stenosis	Heterozygote	VUS	533.42 KB	7
324	1:233098426–245221161	q42.2‐q44	1	Duplication	Pulmonic stenosis	Heterozygote	VUS	12.12 MB	134
325	1:245312845–248918440	q44	1	Deletion	Pulmonic stenosis	Heterozygote	VUS	3.61 MB	101
326	7:73312522–74725117	q11.23	7	Deletion	Pulmonic stenosis	Heterozygote	VUS	1.41 MB	34
327	13:18445861–114344403	q11.34	13	Trisomy	Pulmonic stenosis	Heterozygote	Pathogenic	95.90 MB	
328	3:53927806–55308766	p21.1‐p14.3	3	Duplication	Pulmonic stenosis	Heterozygote	Likely pathogenic	1.38 MB	8
329	6:181563–6144937	p25.3‐p25.1	6	Triplication	Pulmonic stenosis	Heterozygote	Pathogenic	5.96 MB	65
330	19:13822207–16209262	p13.12‐p13.11	19	Deletion	Pulmonic stenosis	Heterozygote	Pathogenic	2.39 MB	109
331	12:56433896–58198236	q13.3‐q14.1	12	Deletion	Pulmonic stenosis	Heterozygote	VUS	1.76 MB	70
332	7:86944768–95002065	q21.12‐q21.3	7	Deletion	Pulmonic stenosis	Heterozygote	Pathogenic	8.06 MB	83
333	7:99120349–107362504	q22.1‐q22.3	7	Deletion	Pulmonic stenosis	Heterozygote	Pathogenic	8.24 MB	195
334	10:26729206–28746254	p12.1	10	Deletion	Pulmonic stenosis	Heterozygote	Pathogenic	2.02 MB	41
335	8:8273108–8496211	p23.1	8	Deletion	Pulmonic stenosis	Heterozygote	VUS	223.10 KB	3
336	22:25770313–29142004	q12.1	22	Deletion	Pulmonic stenosis	Heterozygote	Pathogenic	3.37 MB	44
337	9:28561983–28745048	p21.1	9	Deletion	Pulmonic stenosis	Heterozygote	Likely pathogenic	183.07 KB	2
338	10:79882192–87068232	q22.3‐q23.2	10	Deletion	Pulmonic stenosis	Heterozygote	Likely pathogenic	7.19 MB	80
339	13:33754499–40799535	q13.2‐q14.11	13	Deletion	Pulmonic stenosis	Heterozygote	VUS	7.05 MB	74
340	15:46072402–61905184	q21.1‐q22.2	15	Duplication	Pulmonic stenosis	Heterozygote	Likely pathogenic	15.83 MB	163
341	3:69193959–72127663	p14.1‐p13	3	Deletion	Pulmonic stenosis	Heterozygote	Pathogenic	2.93 MB	21
342	1:115800145–119998439	p13.1‐p12	1	Deletion	Pulmonic stenosis	Heterozygote	VUS	4.20 MB	75
343	3:182657028–184519164	q26.33‐q27.1	3	Deletion	Pulmonic stenosis	Heterozygote	Likely pathogenic	1.86 MB	53
344	7:73228070–74946081	q11.23	7	Deletion	Pulmonic stenosis	Heterozygote	Pathogenic	1.72 MB	44
345	10:90451–1775938	p15.3	10	Deletion	Pulmonic stenosis	Heterozygote	Pathogenic	1.69 MB	17
346	10:1843722–12497634	p15.3‐p13	10	Duplication	Pulmonic stenosis	Heterozygote	Pathogenic	10.65 MB	112
347	20:14648507–14775777	p12.1	20	Deletion	Pulmonic stenosis	Heterozygote	VUS	127.27 KB	2
348	X:148197338–153002821	q28	X	Deletion	Pulmonic stenosis	Heterozygote	VUS	4.81 MB	76
349	3:32034–4308818	p26.3‐p26.1	3	Deletion	Pulmonic stenosis	Heterozygote	VUS	4.28 MB	26
350	3:4341585–6138452	p26.1	3	Duplication	Pulmonic stenosis	Heterozygote	VUS	1.80 MB	13
351	3:196013486–197583580	q29	3	Duplication	Pulmonic stenosis	Heterozygote	VUS	1.57 MB	43
352	2:110287239–110641666	q13	2	Deletion	Pulmonic stenosis	Heterozygote	Pathogenic	354.43 KB	7
353	8:139933282–140075958	q24.3	8	Deletion	Pulmonic stenosis	Heterozygote	VUS	142.68 KB	2
354	8:6285586–6421430	p23.2‐p23.1	8	Duplication	Pulmonic stenosis	Heterozygote	VUS	135.84 KB	2
355	4:64685–10366772	p16.3‐p16.1	4	Deletion	Pulmonic stenosis	Heterozygote	VUS	10.30 MB	204
356	9:122841289–123259829	q33.2‐q33.3	9	Deletion	Pulmonic stenosis	Heterozygote	VUS	418.54 KB	10
357	8:8235543–12031270	p23.1	8	Deletion	Pulmonic stenosis	Heterozygote	Pathogenic	3.80 MB	61
358	5:176143674–177800215	q35.2‐q35.3	5	Duplication	Pulmonic stenosis	Heterozygote	Pathogenic	1.66 MB	48
359	7:65558–6831312	p22.3‐p22.1	7	Duplication	Pulmonic stenosis	Heterozygote	Pathogenic	6.77 MB	111
360	4:71660–3870653	p16.3	4	Deletion	Pulmonic stenosis	Heterozygote	Pathogenic	3.80 MB	77
361	X:5165249–5436978	p22.32	X	Deletion	Pulmonic stenosis	Heterozygote	VUS	271.73 KB	3
362	7:73287279–74732517	q11.23	7	Deletion	Pulmonic stenosis	Heterozygote	Pathogenic	1.45 MB	36
363	15:23123715–42596849	q11.2‐q15.2	15	Duplication	Pulmonic stenosis	Heterozygote	Pathogenic	19.47 MB	370
364	1:61091785–61304381	p31.3	1	Deletion	Pulmonic stenosis	Heterozygote	Pathogenic	212.60 KB	2
365	2:65312462–69370077	p14‐p13.3	2	Deletion	Pulmonic stenosis	Heterozygote	Likely pathogenic	4.06 MB	46
366	8:68978294–71671393	q13.2‐q13.3	8	Deletion	Pulmonic stenosis	Heterozygote	Pathogenic	2.69 MB	26
367	9:98011849–98225122	q22.33	9	Duplication	Pulmonic stenosis	Heterozygote	VUS	213.27 KB	6
368	1:222929033–222939163	q41	1	Deletion	Pulmonic stenosis	Heterozygote	VUS	10.13 KB	1
369	16:80905979–90186088	q23.2‐q24.3	16	Duplication	Pulmonic stenosis	Heterozygote	Pathogenic	9.28 MB	157
370	15:98760770–101843276	q26.3	15	Deletion	Pulmonic stenosis	Heterozygote	Pathogenic	3.08 MB	48
371	16:21940058–22368876	p12.2	16	Deletion	Pulmonic stenosis	Heterozygote	Likely pathogenic	428.82 KB	9
372	1:74432538–74594777	p31.1	1	Deletion	Pulmonic stenosis	Heterozygote	Likely pathogenic	162.24 KB	5
373	4:169902854–177580762	q33‐q34.3	4	Deletion	Pulmonic stenosis	Heterozygote	Pathogenic	7.68 MB	53
374	7:73308984–74715504	q11.23	7	Deletion	Pulmonic stenosis	Heterozygote	Pathogenic	1.41 MB	34
375	1:237110660–248938897	q43‐q44	1	Deletion	Pulmonic stenosis	Heterozygote	Likely pathogenic	11.83 MB	175
376	4:169504119–190107927	q33‐q35.2	4	Deletion	Pulmonic stenosis	Heterozygote	Likely pathogenic	20.60 MB	176
377	8:60001–12798120	p23.3‐p23.1	8	Deletion	Pulmonic stenosis	Heterozygote	Likely pathogenic	12.74 MB	214
378	18:63981786–80256282	q22.1‐q23	18	Deletion	Pulmonic stenosis	Heterozygote	Likely pathogenic	16.27 MB	96
379	6:60001–24999771	p25.3‐p22.3	6	Duplication	Pulmonic stenosis	Heterozygote	Pathogenic	24.94 MB	231
380	3:182999511–198235559	q26.33‐q29	3	Deletion	Pulmonic stenosis	Heterozygote	Pathogenic	15.24 MB	268
381	4:10001–11322107	p16.3‐p15.33	4	Duplication	Pulmonic stenosis	Heterozygote	Likely pathogenic	11.31 MB	208
382	4:10001–11322107	p16.3‐p15.33	4	Deletion	Pulmonic stenosis	Heterozygote	Likely pathogenic	11.31 MB	208
383	21:30205811–46699983	q22.11‐q22.3	21	Duplication	Pulmonic stenosis	Heterozygote	Likely pathogenic	16.49 MB	356
384	5:169040417–181478259	q35.1‐q35.3	5	Deletion	Pulmonic stenosis	Heterozygote	Likely pathogenic	12.44 MB	219
385	7:61071607–77633657	q11.1‐q11.23	7	Deletion	Pulmonic stenosis	Heterozygote	Likely pathogenic	16.56 MB	252
386	10:14061–17217995	p15.3‐p13	10	Deletion	Pulmonic stenosis	Heterozygote	Likely pathogenic	17.20 MB	189
387	9:10001–14110001	p24.3‐p23	9	Deletion	Pulmonic stenosis	Heterozygote	Likely pathogenic	14.10 MB	124
388	X:137892321–154900412	q26.3‐q28	X	Deletion	Pulmonic stenosis	Heterozygote	Likely pathogenic	17.01 MB	265
389	11:130724895–135076622	q24.3‐q25	11	Deletion	Pulmonic stenosis	Heterozygote	Likely pathogenic	4.35 MB	32
390	13:109649654–114344403	q34	13	Deletion	Pulmonic stenosis	Heterozygote	Likely pathogenic	4.69 MB	83
391	4:10001–11322107	p16.3‐p15.33	4	Deletion	Pulmonic stenosis	Heterozygote	Likely pathogenic	11.31 MB	208
392	15:97955767–101981189	q26.2‐q26.3	15	Deletion	Pulmonic stenosis	Heterozygote	Likely pathogenic	4.03 MB	66
393	5:10001–18464134	p15.33‐p14.3	5	Deletion	Pulmonic stenosis	Heterozygote	Likely pathogenic	18.45 MB	186
394	11:110624066–135076622	q23.1‐q25	11	Duplication	Pulmonic stenosis	Heterozygote	Likely pathogenic	24.45 MB	412
395	13:93249746–113340664	q31.3‐q34	13	Deletion	Pulmonic stenosis	Heterozygote	Likely pathogenic	20.09 MB	209
396	21:38306208–46699983	q22.2‐q22.3	21	Deletion	Pulmonic stenosis	Heterozygote	Likely pathogenic	8.39 MB	193
397	11:130724895–135076622	q24.3‐q25	11	Duplication	Pulmonic stenosis	Heterozygote	Likely pathogenic	4.35 MB	32
398	7:10239–2787125	p22.3	7	Duplication	Pulmonic stenosis	Heterozygote	Likely pathogenic	2.78 MB	50
399	17:150208–6755957	p13.3‐p13.1	17	Deletion	Pulmonic stenosis	Heterozygote	Likely pathogenic	6.61 MB	188
400	9:10001–9010000	p24.3‐p23	9	Deletion	Pulmonic stenosis	Heterozygote	Likely pathogenic	9.00 MB	105
401	14:87463903–105773834	q31.3‐q32.33	14	Deletion	Pulmonic stenosis	Heterozygote	Likely pathogenic	18.31 MB	387
402	15:88455765–101898930	q25.3‐q26.3	15	Duplication	Pulmonic stenosis	Heterozygote	Likely pathogenic	13.44 MB	182
403	18:10001–15410899	p11.32‐p11.1	18	Deletion	Pulmonic stenosis	Heterozygote	Likely pathogenic	15.40 MB	200
404	5:169040417–181478259	q35.1‐q35.3	5	Duplication	Pulmonic stenosis	Heterozygote	Likely pathogenic	12.44 MB	219
405	4:107021239–123121240	q25‐q28.1	4	Deletion	Pulmonic stenosis	Heterozygote	Likely pathogenic	16.10 MB	173
406	6:164008557–170687856	q26‐q27	6	Deletion	Pulmonic stenosis	Heterozygote	Likely pathogenic	6.68 MB	63
407	1:235310645–248938897	q42.3‐q44	1	Deletion	Pulmonic stenosis	Heterozygote	Likely pathogenic	13.63 MB	208
408	20:9171353–21371362	p12.3‐p11.22	20	Deletion	Pulmonic stenosis	Heterozygote	Likely pathogenic	12.20 MB	127
409	8:6355071–28886564	p23.1‐p21.1	8	Deletion	Pulmonic stenosis	Heterozygote	Likely Pathogenic	22.53 MB	353
410	19:42904008–58603472	q13.31‐q13.43	19	Duplication	Pulmonic stenosis	Heterozygote	Likely pathogenic	15.70 MB	785
411	17:80311606–83101964	q25.3	17	Deletion	Pulmonic stenosis	Heterozygote	Likely pathogenic	2.79 MB	94
412	17:150208–11055958	p13.3‐p12	17	Duplication	Pulmonic stenosis	Heterozygote	Likely pathogenic	10.91 MB	332
413	7:138458000–159253352	q33‐q36.3	7	Deletion	Pulmonic stenosis	Heterozygote	Likely pathogenic	20.80 MB	391
414	19:60001–6948989	p13.3‐p13.2	19	Deletion	Pulmonic stenosis	Heterozygote	Likely pathogenic	6.89 MB	260
415	19:55796823–58603472	q13.42‐q13.43	19	Deletion	Pulmonic stenosis	Heterozygote	Likely pathogenic	2.81 MB	127
416	18:75459057–80256282	q23	18	Deletion	Pulmonic stenosis	Heterozygote	Likely pathogenic	4.80 MB	36
417	17:75992325–83101964	q25.1‐q25.3	17	Duplication	Pulmonic stenosis	Heterozygote	Likely pathogenic	7.11 MB	200
418	4:10001–19022108	p16.3‐p15.31	4	Deletion	Pulmonic stenosis	Heterozygote	Likely pathogenic	19.01 MB	262
419	4:182603698–190107927	q35.1‐q35.2	4	Duplication	Pulmonic stenosis	Heterozygote	Likely pathogenic	7.50 MB	106
420	22:17446408–25579479	q11.21‐q12.1	22	Deletion	Pulmonic stenosis	Heterozygote	Likely pathogenic	8.13 MB	332
421	11:115124070–135076622	q23.3‐q25	11	Deletion	Pulmonic stenosis	Heterozygote	Likely pathogenic	19.95 MB	330
422	4:10001–15822108	p16.3‐p15.32	4	Deletion	Pulmonic stenosis	Heterozygote	Likely pathogenic	15.81 MB	238
423	16:59308595–72008600	q21‐q22.2	16	Deletion	Pulmonic stenosis	Heterozygote	Likely pathogenic	12.70 MB	191
424	9:99836718–124836711	q31.1‐q33.3	9	Duplication	Pulmonic stenosis	Heterozygote	Pathogenic	25.00 MB	273
425	2:230509781–242160331	q37.1‐q37.3	2	Duplication	Pulmonic stenosis	Heterozygote	Likely pathogenic	11.65 MB	211
426	8:18798210–39423324	p22‐p11.22	8	Deletion	Pulmonic stenosis	Heterozygote	Likely pathogenic	20.63 MB	234
427	19:60001–12628186	p13.3‐p13.13	19	Duplication	Pulmonic stenosis	Heterozygote	Likely pathogenic	12.57 MB	487
428	11:68775956–88627184	q13.3‐q14.3	11	Duplication	Pulmonic stenosis	Heterozygote	Likely pathogenic	19.85 MB	294
429	4:5780201–28322109	p16.2‐p15.1	4	Deletion	Pulmonic stenosis	Heterozygote	Likely pathogenic	22.54 MB	204
430	22:16367189–37152953	q11.1‐q12.3	22	Duplication	Pulmonic stenosis	Heterozygote	Likely pathogenic	20.79 MB	583
431	20:17871356–25871364	p12.1‐p11.1	20	Duplication	Pulmonic stenosis	Heterozygote	Likely pathogenic	8.00 MB	135
432	6:148437171–170687856	q24.3‐q27	6	Deletion	Pulmonic stenosis	Heterozygote	Likely pathogenic	22.25 MB	229
433	4:10001–11322107	p16.3‐p15.33	4	Deletion	Pulmonic stenosis	Heterozygote	Likely pathogenic	11.31 MB	208
434	6:160508557–170687856	q25.3‐q27	6	Deletion	Pulmonic stenosis	Heterozygote	Likely pathogenic	10.18 MB	78
435	4:138721241–154821243	q31.1‐q32.1	4	Deletion	Pulmonic stenosis	Heterozygote	Likely pathogenic	16.10 MB	157
436	19:60001–13828186	p13.3‐p13.12	19	Duplication	Pulmonic stenosis	Heterozygote	Likely pathogenic	13.77 MB	535
437	20:17871357–25771364	p12.1‐p11.1	20	Duplication	Pulmonic stenosis	Heterozygote	Likely pathogenic	7.90 MB	133
438	4:175703700–190107927	q34.2‐q35.2	4	Deletion	Pulmonic stenosis	Heterozygote	Likely pathogenic	14.40 MB	134
439	18:71081784–80256282	q22.3‐q23	18	Deletion	Pulmonic stenosis	Heterozygote	Likely pathogenic	9.17 MB	62
440	4:10001–4380201	p16.3	4	Deletion	Pulmonic stenosis	Heterozygote	Likely pathogenic	4.37 MB	94
441	11:60001–2822194	p15.5‐p15.4	11	Duplication	Pulmonic stenosis	Heterozygote	Likely pathogenic	2.76 MB	115
442	10:116650499–133334830	q25.3‐q26.3	10	Deletion	Pulmonic stenosis	Heterozygote	Likely pathogenic	16.68 MB	180
443	8:2266819–12798120	p23.3‐p23.1	8	Deletion	Pulmonic stenosis	Heterozygote	Likely pathogenic	10.53 MB	193
444	20:5071354–21371362	p13‐p11.22	20	Deletion	Pulmonic stenosis	Heterozygote	Likely pathogenic	16.30 MB	167
445	7:67590864–77926032	q11.22‐q21.11	7	Deletion	Pulmonic stenosis	Heterozygote	Likely pathogenic	10.34 MB	145
446	4:171004120–190107927	q34.1‐q35.2	4	Deletion	Pulmonic stenosis	Heterozygote	Likely pathogenic	19.10 MB	163
447	8:6355071–12798120	p23.1	8	Deletion	Pulmonic stenosis	Heterozygote	Likely pathogenic	6.44 MB	186
448	22:16367189–25579479	q11.1‐q12.1	22	Duplication	Pulmonic stenosis	Heterozygote	Likely pathogenic	9.21 MB	372
449	18:10001–27420036	p11.32‐q11.2	18	Deletion	Pulmonic stenosis	Heterozygote	Likely pathogenic	27.41 MB	277
450	17:150208–22763679	p13.3‐p11.1	17	Duplication	Pulmonic stenosis	Heterozygote	Likely pathogenic	22.61 MB	587
451	12:14655799–33155799	p12.3‐p11.21	12	Deletion	Pulmonic stenosis	Heterozygote	Likely pathogenic	18.50 MB	179
452	3:67938047–85499843	p14.1‐p12.1	3	Deletion	Pulmonic stenosis	Heterozygote	Likely pathogenic	17.56 MB	114
453	8:2266820–12798120	p23.3‐p23.1	8	Deletion	Pulmonic stenosis	Heterozygote	Likely pathogenic	10.53 MB	193
454	15:19961586–32045284	q11.1‐q13.3	15	Duplication	Pulmonic stenosis	Heterozygote	Likely pathogenic	12.08 MB	291
455	22:20387910–21107568	q11.21	22	Deletion	Pulmonic stenosis	Heterozygote	Likely pathogenic	719.66 KB	32
456	6:164108558–170745979	q27	6	Duplication	Pulmonic stenosis	Heterozygote	Likely pathogenic	6.64 MB	64
457	9:14210374–16338034	p22.3	9	Deletion	Pulmonic stenosis	Heterozygote	Likely pathogenic	2.13 MB	23
458	16:14991897–18170481	p13.11‐p12.3	16	Duplication	Pulmonic stenosis	Heterozygote	Likely pathogenic	3.18 MB	35
459	8:114180690–137750255	q23.3‐q24.23	8	Deletion	Pulmonic stenosis	Heterozygote	Likely pathogenic	23.57 MB	172
460	17:2918018–6298546	p13.3‐p13.2	17	Deletion	Pulmonic stenosis	Heterozygote	Likely pathogenic	3.38 MB	104
461	1:914086–2516854	p36.33‐p36.32	1	Deletion	Pulmonic stenosis	Heterozygote	Likely pathogenic	1.60 MB	77
462	7:74060528–74089583	q11.23	7	Deletion	Pulmonic stenosis	Heterozygote	Likely pathogenic	29.06 KB	3
463	11:1925415–5476387	p15.5‐p15.4	11	Duplication	Pulmonic stenosis	Heterozygote	Likely pathogenic	3.55 MB	157
464	9:13155536–14115572	p23	9	Deletion	Pulmonic stenosis	Heterozygote	Likely pathogenic	960.04 KB	7
465	3:196004885–197612599	q29	3	Deletion	Pulmonic stenosis	Heterozygote	Likely pathogenic	1.61 MB	43
466	7:72950006–76680766	q11.23	7	Deletion	Pulmonic stenosis	Heterozygote	Likely pathogenic	3.73 MB	102
467	8:29904086–37638266	p12‐p11.23	8	Deletion	Pulmonic stenosis	Heterozygote	Likely pathogenic	7.73 MB	64
468	5:77568419–93038538	q13.3‐q15	5	Deletion	Pulmonic stenosis	Heterozygote	Likely pathogenic	15.47 MB	129
469	9:10001–19910002	p24.3‐p21.3	9	Deletion	Pulmonic stenosis	Heterozygote	Likely pathogenic	19.90 MB	173
470	1:147034756–148352079	q21.1‐q21.2	1	Deletion	Pulmonic stenosis	Heterozygote	Pathogenic	1.32 MB	36
471	X:85834323–86460905	q21.2	X	Duplication	Pulmonic stenosis	Heterozygote	VUS	626.58 KB	10
472	2:239045268–242099213	q37.3	2	Deletion	Pulmonic stenosis	Heterozygote	Pathogenic	3.05 MB	60
473	X:11091–4781375	p22.33‐p22.32	X	Deletion	Pulmonic stenosis	Heterozygote	Pathogenic	4.77 MB	48
474	7:40264549–40340443	p14.1	7	Deletion	Pulmonic stenosis	Heterozygote	VUS	75.89 KB	1
475	4:126938401–128222207	q28.1‐q28.2	4	Duplication	Pulmonic stenosis	Heterozygote	VUS	1.28 MB	9
476	8:8244119–12002342	p23.1	8	Deletion	Pulmonic stenosis	Heterozygote	Pathogenic	3.76 MB	58
477	6:148758327–151251017	q25.1	6	Deletion	Pulmonic stenosis	Heterozygote	VUS	2.49 MB	43
478	13:18722404–21586267	q11‐q12.11	13	Duplication	Pulmonic stenosis	Heterozygote	VUS	2.86 MB	81
479	X:131613445–131916984	q26.2	X	Duplication	Pulmonic stenosis	Heterozygote	VUS	303.54 KB	5
480	7:74031409–74110950	q11.23	7	Deletion	Pulmonic stenosis	Heterozygote	Pathogenic	79.54 KB	3
481	13:22992823–24365462	q12.12	13	Deletion	Pulmonic stenosis	Heterozygote	VUS	1.37 MB	26
482	15:33054514–33695455	q13.3‐q14	15	Duplication	Pulmonic stenosis	Heterozygote	VUS	640.94 KB	4
483	18:52376298–80254946	q21.2‐q23	18	Monosomy	Pulmonic stenosis	Heterozygote	Pathogenic	27.88 MB	212
484	18:60914122–80244381	q21.32‐q23	18	Deletion	Pulmonic stenosis	Heterozygote	Pathogenic	19.33 MB	126
485	22:37124249–50295602	q12.3‐q13.33	22	Duplication	Pulmonic stenosis	Heterozygote	Likely pathogenic	13.17 MB	282
486	X:44956231–45164142	p11.3	X	Duplication	Pulmonic stenosis	Heterozygote	VUS	207.91 KB	2
487	10:84211438–84367966	q23.1	10	Duplication	Pulmonic stenosis	Heterozygote	VUS	156.53 KB	6
488	10:84374979–94104833	q23.1‐q23.33	10	Duplication/Triplication	Pulmonic stenosis	Heterozygote	Likely pathogenic	9.73 MB	143
489	20:64164668–64241486	q13.33	20	Deletion	Pulmonic stenosis	Heterozygote	VUS	76.82 KB	1
490	19:46985018–47155132	q13.32	19	Duplication	Pulmonic stenosis	Heterozygote	VUS	170.12 KB	5
491	18:14316–12274495	p11.32‐p11.21	18	Duplication	Pulmonic stenosis	Heterozygote	Pathogenic	12.26 MB	146
492	18:57137419–80224243	q21.31‐q23	18	Deletion	Pulmonic stenosis	Heterozygote	Likely pathogenic	23.09 MB	176
493	7:8136670–8545866	p21.3	7	Duplication	Pulmonary insufficiency	Heterozygote	VUS	409.20 KB	3
494	15:22657386–23095194	q11.2	15	Deletion	Pulmonary insufficiency	Heterozygote	VUS	437.81 KB	7
495	4:156882124–189800812	q32.1‐q35.2	4	Deletion	Pulmonary insufficiency	Heterozygote	VUS	32.92 MB	242
496	3:192924147–198043535	q29	3	Duplication	Pulmonary insufficiency	Heterozygote	VUS	5.12 MB	115
497	8:60001–12798120	p23.3‐p23.1	8	Deletion	Pulmonary insufficiency	Heterozygote	Likely pathogenic	12.74 MB	214
498	7:148165260–159253352	q35‐q36.3	7	Deletion	Pulmonary insufficiency	Heterozygote	Likely pathogenic	11.09 MB	147
499	14:100863910–105773834	q32.2‐q32.33	14	Deletion	Pulmonary insufficiency	Heterozygote	Likely pathogenic	4.91 MB	216
500	18:71081784–80256282	q22.3‐q23	18	Deletion	Pulmonary insufficiency	Heterozygote	Likely pathogenic	9.17 MB	62
501	2:235309357–242160331	q37.2‐q37.3	2	Duplication	Pulmonary insufficiency	Heterozygote	Likely pathogenic	6.85 MB	107
502	22:17446408–25579479	q11.21‐q12.1	22	Deletion	Pulmonary insufficiency	Heterozygote	Likely pathogenic	8.13 MB	332
503	18:10001–15410899	p11.32‐p11.1	18	Triplication	Pulmonary insufficiency	Heterozygote	Likely pathogenic	15.40 MB	200
504	6:160508557–170687856	q25.3‐q27	6	Deletion	Pulmonary insufficiency	Heterozygote	Likely pathogenic	10.18 MB	78
505	18:75459057–80256282	q23	18	Deletion	Pulmonary insufficiency	Heterozygote	Likely pathogenic	4.80 MB	36
506	16:21937751–22412377	p12.2	16	Deletion	Hypertension	Heterozygote	VUS	474.63 KB	11
507	22:22687273–23317723	q11.22‐q11.23	22	Deletion	Hypertension	Heterozygote	VUS	630.45 KB	49
508	22:22728147–22880538	q11.22	22	Deletion	Hypertension	Heterozygote	VUS	152.39 KB	18
509	3:59717083–61035866	p14.2	3	Duplication	Hypertension	Heterozygote	VUS	1.32 MB	5
510	10:123496700–133769367	q26.13‐q26.3	10	Duplication	Hypertension	Heterozygote	VUS	10.27 MB	124
511	11:127562910–135074876	q24.2‐q25	11	Deletion	Hypertension	Heterozygote	VUS	7.51 MB	69
512	16:29837876–30179218	p11.2	16	Deletion	Hypertension	Heterozygote	VUS	341.34 KB	21
513	4:174735417–178345522	q34.1‐q34.3	4	Deletion	Hypertension	Heterozygote	VUS	3.61 MB	22
514	1:45350307–45470048	p34.1	1	Deletion	Hypertension	Heterozygote	VUS	119.74 KB	2
515	6:75633443–84537981	q14.1‐q14.3	6	Deletion	Hypertension	Heterozygote	VUS	8.90 MB	53
516	9:117132868–118101205	q33.1	9	Duplication	Hypertension	Heterozygote	VUS	968.34 KB	9
517	5:35588987–39328404	p13.2‐p13.1	5	Duplication	Hypertension	Heterozygote	VUS	3.74 MB	50
518	17:496040–1368660	p13.3	17	Duplication	Hypertension	Heterozygote	VUS	872.62 KB	15
519	4:10026273–10551262	p16.1	4	Duplication	Hypertension	Heterozygote	VUS	524.99 KB	7
520	14:29312198–30083730	q12	14	Deletion	Hypertension	Heterozygote	VUS	771.53 KB	3
521	6:203288–11735845	p25.3‐p24.1	6	Duplication	Hypertension	Heterozygote	Pathogenic	11.53 MB	120
522	15:31719559–32225000	q13.3	15	Duplication	Hypertension	Heterozygote	VUS	505.44 KB	3
523	X:80673777–80736644	q21.1	X	Deletion	Hypertension	Hemizygous	VUS	62.87 KB	2
524	8:1817367–7081774	p23.3‐p23.1	8	Duplication	Hypertension	Heterozygote	Pathogenic	5.26 MB	37
525	8:12698495–22959073	p23.1‐p21.3	8	Deletion	Hypertension	Heterozygote	Pathogenic	10.26 MB	94
526	X:6495863–8147172	p22.31	X	Deletion	Hypertension	Hemizygous	VUS	1.65 MB	8
527	1:236127731–236511859	q42.3‐q43	1	Duplication	Hypertension	Heterozygote	VUS	384.13 KB	5
528	22:18906348–21048193	q11.21	22	Duplication	Hypertension	Heterozygote	Likely pathogenic	2.14 MB	84
529	8:6518891–6801141	p23.1	8	Duplication	Hypertension	Heterozygote	Pathogenic	282.25 KB	7
530	8:6558958–6745909	p23.1	8	Duplication	Hypertension	Heterozygote	VUS	186.95 KB	7
531	16:14792538–16526893	p13.11	16	Duplication	Hypertension	Heterozygote	Pathogenic	1.73 MB	41
532	7:43376–9415156	p22.3‐p21.3	7	Duplication	Hypertension	Heterozygote	Pathogenic	9.37 MB	133
533	17:59919654–61940527	q23.1‐q23.2	17	Deletion	Hypertension	Heterozygote	Likely pathogenic	2.02 MB	38
534	16:80905979–90186088	q23.2‐q24.3	16	Duplication	Hypertension	Heterozygote	Pathogenic	9.28 MB	157
535	15:98760770–101843276	q26.3	15	Deletion	Hypertension	Heterozygote	Pathogenic	3.08 MB	48
536	7:10239–2887125	p22.3‐p22.2	7	Deletion	Hypertension	Heterozygote	Likely pathogenic	2.88 MB	50
537	7:155270643–159335973	q36.3	7	Deletion	Hypertension	Heterozygote	Likely pathogenic	4.07 MB	37
538	4:23122108–41357055	p15.2‐p13	4	Deletion	Hypertension	Heterozygote	Likely pathogenic	18.23 MB	118
539	15:19794748–33320507	q11.1‐q13.3	15	Deletion	Hypertension	Heterozygote	Likely pathogenic	13.53 MB	317
540	18:10001–15410001	p11.32‐p11.1	18	Deletion	Hypertension	Heterozygote	Likely pathogenic	15.40 MB	200
541	17:73702165–74672979	q25.1	17	Duplication	Hypertension	Heterozygote	Likely pathogenic	970.82 KB	19
542	5:22149–21269047	p15.33‐p14.3	5	Duplication	Hypertension	Heterozygote	VUS	21.25 MB	195
543	6:204009–1386819	p25.3	6	Deletion	Hypertension	Heterozygote	VUS	1.18 MB	8
544	18:59502100–80256949	q21.32‐q23	18	Deletion	Hypertension	Heterozygote	Likely pathogenic	20.75 MB	143
545	17:31095176–31095461	q11.2	17	Deletion	Hypertension	Heterozygote	Pathogenic	286 BB	2
546	16:73977–546314	p13.3	16	Duplication	Hypertension	Heterozygote	Likely pathogenic	472.34 KB	26
547	7:73210658–74801875	q11.23	7	Deletion	Hypertension	Heterozygote	Pathogenic	1.59 MB	41
548	22:38177001–50665472	q13.1‐q13.33	22	Duplication	Portal hypertension	Heterozygote	Likely pathogenic	12.49 MB	266
549	4:87914850–88246255	q22.1	4	Deletion	Low‐to‐normal blood pressure	Heterozygote	Likely pathogenic	331.41 KB	7
550	X:66188608–66631460	q12	X	Duplication	Orthostatic hypotension	Heterozygote	VUS	442.85 KB	4
551	12:121255–6038780	p13.33‐p13.31	12	Deletion	Vasovagal syncope	Heterozygote	Pathogenic	5.92 MB	78
552	16:81239406–90028073	q23.2‐q24.3	16	Duplication	Vasovagal syncope	Heterozygote	Pathogenic	8.79 MB	141
553	4:8225924–8336806	p16.1	4	Deletion	Subdural haemorrhage	Heterozygote	VUS	110.88 KB	3
554	22:28248728–28547489	q12.1	22	Duplication	Subdural haemorrhage	Heterozygote	VUS	298.76 KB	2
555	X:18490854–18605032	p22.13	X	Duplication	Subdural haemorrhage	Heterozygote	Likely pathogenic	114.18 KB	2
556	22:18902649–21186058	q11.21	22	Deletion	Subdural haemorrhage	Heterozygote	Pathogenic	2.28 MB	89
557	X:111196251–123377432	q23‐q25	X	Duplication	Subdural haemorrhage	Heterozygote	VUS	12.18 MB	153
558	16:15037866–16155750	p13.11	16	Duplication	Grade I preterm intraventricular haemorrhage	Heterozygote	Likely pathogenic	1.12 MB	21
559	9:33414186–39156957	p13.3‐p12	9	Deletion	Grade I preterm intraventricular haemorrhage	Heterozygote	Pathogenic	5.74 MB	166
560	10:14061–2967808	p15.3	10	Deletion	Antepartum haemorrhage	Heterozygote	Likely pathogenic	2.95 MB	26
561	10:116950500–133787422	q25.3‐q26.3	10	Deletion	Antepartum haemorrhage	Heterozygote	Likely pathogenic	16.84 MB	208
562	8:60001–12798120	p23.3‐p23.1	8	Deletion	Antepartum haemorrhage	Heterozygote	Likely pathogenic	12.74 MB	214
563	X:10001–24622226	p22.33‐p22.11	X	Deletion	Antepartum haemorrhage	Heterozygote	Likely pathogenic	24.61 MB	227
564	18:10001–15410899	p11.32‐p11.1	18	Duplication	Antepartum haemorrhage	Heterozygote	Likely pathogenic	15.40 MB	200
565	6:60001–15191790	p25.3‐p23	6	Deletion	Antepartum haemorrhage	Heterozygote	Likely pathogenic	15.13 MB	149
566	9:132995060–136245043	q34.13‐q34.3	9	Deletion	Antepartum haemorrhage	Heterozygote	Likely pathogenic	3.25 MB	77
567	12:45001–10056134	p13.33‐p13.2	12	Deletion	Antepartum haemorrhage	Heterozygote	Likely pathogenic	10.01 MB	227
568	12:120199477–132891443	q24.23‐q24.33	12	Duplication	Antepartum haemorrhage	Heterozygote	Likely pathogenic	12.69 MB	201
569	19:60001–12628186	p13.3‐p13.13	19	Duplication	Antepartum haemorrhage	Heterozygote	Likely pathogenic	12.57 MB	487
570	1:49922308–60422307	p33.p32.1	1	Deletion	Antepartum haemorrhage	Heterozygote	Likely pathogenic	10.50 MB	154
571	4:10001–4380201	p16.3	4	Deletion	Antepartum haemorrhage	Heterozygote	Likely pathogenic	4.37 MB	94
572	4:10001–11322107	p16.3‐p15.33	4	Deletion	Antepartum haemorrhage	Heterozygote	Likely pathogenic	11.31 MB	208
573	13:93249746–113340664	q31.3‐q34	13	Deletion	Antepartum haemorrhage	Heterozygote	Likely pathogenic	20.09 MB	209
574	22:21821157–23133259	q11.22‐q11.23	22	Deletion	Antepartum haemorrhage	Heterozygote	Likely pathogenic	1.31 MB	112
575	3:18323–16383489	p26.3‐p24.3	3	Deletion	Antepartum haemorrhage	Heterozygote	Likely pathogenic	16.37 MB	191
576	22:16367189–25579479	q11.1‐q12.1	22	Triplication	Antepartum haemorrhage	Heterozygote	Likely pathogenic	9.21 MB	372
577	21:34353927–34533685	q22.11‐q22.12	21	Duplication	Antepartum haemorrhage	Heterozygote	Likely pathogenic	179.76 KB	6
578	21:14166659–20412272	q11.2‐q21.1	21	Deletion	Antepartum haemorrhage	Heterozygote	Likely pathogenic	6.25 MB	55
579	22:44037805–50739836	q13.31‐q13.33	22	Deletion	Antepartum haemorrhage	Heterozygote	Likely pathogenic	6.70 MB	108
580	X:154762806–154769457	q28	X	Duplication	Vitreous haemorrhage	Heterozygote	Likely pathogenic	6.65 KB	3
581	22:18907322–21109830	q11.21	22	Deletion	Retinal haemorrhage	Heterozygote	Pathogenic	2.20 MB	85
582	19:3769051–4128726	p13.3	19	Deletion	Raynaud phenomenon	Heterozygote	Pathogenic	359.68 KB	14
583	19:53042501–53219888	q13.41‐q13.42	19	Deletion	Transient ischemic attack	Heterozygote	VUS	177.39 KB	6
584	13:40782715–64043738	q14.11‐q21.31	13	Deletion	Transient ischemic attack	Heterozygote	Pathogenic	23.26 MB	280
585	19:53042501–53219888	q13.41‐q13.42	19	Deletion	Transient ischemic attack	Heterozygote	VUS	177.39 KB	6
586	13:40782715–64043738	q14.11‐q21.31	13	Deletion	Transient ischemic attack	Heterozygote	Pathogenic	23.26 MB	280
587	7:97963126–98710291	q21.3‐q22.1	7	Deletion	Pulmonary venous hypertension	Heterozygote	VUS	747.17 KB	14
588	1:230644357–232248217	q42.2	1	Deletion	Pulmonary arterial hypertension	Heterozygote	VUS	1.60 MB	26
589	8:36935579–43478495	p11.23‐p11.1	8	Duplication	Pulmonary arterial hypertension	Heterozygote	VUS	6.54 MB	89
590	1:618888–3416040	p36.33‐p36.32	1	Deletion	Pulmonary arterial hypertension	Heterozygote	VUS	2.80 MB	105
591	21:24346438–24930774	q21.2	21	Duplication	Pulmonary arterial hypertension	Heterozygote	VUS	584.34 KB	2
592	18:21838370–37913418	q11.2‐q12.2	18	Duplication	Pulmonary arterial hypertension	Heterozygote	Likely pathogenic	16.08 MB	129
593	2:161979940–164197324	q24.2‐q24.3	2	Deletion	Pulmonary arterial hypertension	Heterozygote	Pathogenic	2.22 MB	15
594	3:71754635–71755198	p13	3	Duplication	Pulmonary arterial hypertension	Heterozygote	Likely PATHOGENIC	564 BP	2
595	4:118587452–176410394	q26‐q34.2	4	Duplication	Pulmonary arterial hypertension	Heterozygote	Pathogenic	57.82 MB	440
596	4:177487644–189193772	q34.3‐q35.2	4	Deletion	Pulmonary arterial hypertension	Heterozygote	Pathogenic	11.71 MB	96
597	21:30635100–33096932	q22.11	21	Triplication	Pulmonary arterial hypertension	Heterozygote	VUS	2.46 MB	47
598	X:154762806–154769457	q28	X	Duplication	Pulmonary arterial hypertension	Heterozygote	Likely pathogenic	6.65 KB	3
599	18:148963–12790521	p11.32‐p11.21	18	Duplication	Pulmonary arterial hypertension	Heterozygote	Pathogenic	12.64 MB	151
600	18:64809105–80254946	q22.1‐q23	18	Deletion	Pulmonary arterial hypertension	Heterozygote	Pathogenic	15.45 MB	91
601	2:168873339–169042705	q24.3‐q31.1	2	Deletion	Pulmonary arterial hypertension	Heterozygote	VUS	169.37 KB	3
602	3:32241–9647908	p26.3‐p25.3	3	Deletion	Pulmonary arterial hypertension	Heterozygote	Pathogenic	9.62 MB	62
603	16:70749398–90044855	q22.1‐q24.3	16	Duplication	Pulmonary arterial hypertension	Heterozygote	Pathogenic	19.30 MB	258
604	17:59919654–61940527	q23.1‐q23.2	17	Deletion	Pulmonary arterial hypertension	Heterozygote	Likely pathogenic	2.02 MB	38
605	17:80017160–83084062	q25.3	17	Duplication	Pulmonary arterial hypertension	Heterozygote	Likely pathogenic	3.07 MB	102
606	5:174340672–181292788	q35.2‐q35.3	5	Deletion	Pulmonary arterial hypertension	Heterozygote	Likely pathogenic	6.95 MB	149
607	22:33519384–36552435	q12.3	22	Deletion	Pulmonary arterial hypertension	Heterozygote	VUS	3.03 MB	40
608	16:82280425–90058024	q23.3‐q24.3	16	Duplication/Triplication	Pulmonary arterial hypertension	Heterozygote	Pathogenic	7.78 MB	129
609	X:75570631–75606484	q13.3	X	Deletion	Pulmonary arterial hypertension	Heterozygote	VUS	35.85 KB	1
610	9:3286700–4051530	p24.2	9	Duplication	Pulmonary arterial hypertension	Heterozygote	VUS	764.83 KB	4
611	6:139751940–151251065	q24.1‐q25.1	6	Deletion	Pulmonary arterial hypertension	Heterozygote	VUS	11.50 MB	101
612	9:27962012–28083674	p21.2‐p21.1	9	Deletion	Pulmonary arterial hypertension	Heterozygote	VUS	121.66 KB	1
613	21:45227163–46680243	q22.3	21	Duplication	Pulmonary arterial hypertension	Heterozygote	Likely pathogenic	1.45 MB	34
614	2:161979940–164197324	q24.2‐q24.3	2	Deletion	High‐output congestive heart failure	Heterozygote	Pathogenic	2.22 MB	15
615	8:6366725–6424109	p23.1	8	Deletion	Right bundle branch block	Heterozygote	VUS	57.38 KB	2
616	5:106094496–106807476	q21.3	5	Deletion	Atrioventricular block	Heterozygote	VUS	712.98 KB	0
617	X:7349823–8127240	p22.31	X	Duplication	Atrioventricular block	Heterozygote	VUS	777.42 KB	4
618	6:136408405–150720236	q23.3‐q25.1	6	Deletion	Atrioventricular block	Heterozygote	VUS	14.31 MB	139
619	3:130125618–130328473	q22.1	3	Deletion	Atrioventricular block	Heterozygote	VUS	202.86 KB	4
620	5:172058335–174409159	q35.1‐q35.2	5	Deletion	Atrioventricular block	Heterozygote	Pathogenic	2.35 MB	34
621	1:243561658–244738083	q44	1	Deletion	Atrioventricular block	Heterozygote	Likely pathogenic	1.18 MB	12
622	6:123216338–123636797	q22.31	6	Duplication	ST segment elevation	Heterozygote	VUS	420.46 KB	2
623	1:115700007–115744912	p13.1	1	Duplication	ST segment elevation	Heterozygote	VUS	44.91 KB	1
624	4:185501697–185535508	q35.1	4	Deletion	Sudden cardiac death	Heterozygote	VUS	33.81 KB	1
625	1:115700007–115744912	p13.1	1	Duplication	Sudden cardiac death	Heterozygote	VUS	44.91 KB	1
626	12:32896509–32896756	p11.21	12	Deletion	Sudden cardiac death	Heterozygote	Pathogenic	248 BP	1
627	X:154379690–154380802	q28	X	Duplication	Sudden cardiac death	Heterozygote	Likely pathogenic	1.11 KB	1
628	19:55151770–55156644	q13.42	19	Duplication	Sudden cardiac death	Heterozygote	Likely pathogenic	4.88 KB	1
629	X:154379197–154421726	q28	X	Duplication	Sudden cardiac death	Heterozygote	VUS	42.53 KB	5
630	17:8288497–8290092	p13.1	17	Duplication	Sudden cardiac death	Heterozygote	VUS	1.60 KB	2
631	6:73479732–73497896	q13	6	Deletion	Sudden cardiac death	Heterozygote	VUS	18.16 KB	3
632	5:122927590–124065270	q23.2	5	Duplication	Wolff–Parkinson–White syndrome	Heterozygote	VUS	1.14 MB	13
633	17:36459737–37889943	q12	17	Deletion	Wolff–Parkinson–White syndrome	Heterozygote	VUS	1.43 MB	21
634	2:233346901–233462705	q37.1	2	Duplication	Premature ventricular contraction	Heterozygote	VUS	115.81 KB	2
635	6:123216338–123636797	q22.31	6	Duplication	Ventricular fibrillation	Heterozygote	VUS	420.46 KB	2
636	1:115700007–115744912	p13.1	1	Duplication	Ventricular fibrillation	Heterozygote	VUS	44.91 KB	1
637	1:20214248–22563917	p36.12	1	Deletion	Supraventricular tachycardia	Heterozygote	Likely pathogenic	2.35 MB	53
638	22:20713403–21111370	q11.21	22	Deletion	Supraventricular tachycardia	Heterozygote	VUS	397.97 KB	16
639	11:18278567–30302330	p15.1‐p14.1	11	Deletion	Supraventricular tachycardia	Heterozygote	Pathogenic	12.02 MB	106
640	4:71661–547305	p16.3	4	Deletion	Supraventricular tachycardia	Heterozygote	Likely pathogenic	475.64 KB	11
641	3:58342111–58475638	p14.3	3	Deletion	Supraventricular tachycardia	Heterozygote	VUS	133.53 KB	2
642	1:233008441–248918469	p24.3‐p23	1	Deletion	Supraventricular tachycardia	Heterozygote	Pathogenic	11.02 MB	101
643	9:204193–11219608	q42.2‐q44	9	Duplication	Supraventricular tachycardia	Heterozygote	Pathogenic	15.91 MB	235
644	9:3286700–4051530	p24.2	9	Duplication	Supraventricular tachycardia	Heterozygote	VUS	764.83 KB	4
645	20:63928257–64198942	q13.33	20	Deletion	Paroxysmal atrial fibrillation	Heterozygote	VUS	270.69 KB	17
646	16:21940058–22368876	p12.2	16	Deletion	Atrial flutter	Heterozygote	Likely pathogenic	428.82 KB	9
647	1:74432538–74594777	p31.1	1	Deletion	Atrial flutter	Heterozygote	Likely pathogenic	162.24 KB	5
648	1:20214248–22563917	p36.12	1	Deletion	Supraventricular tachycardia	Heterozygote	Likely pathogenic	2.35 MB	53
649	22:20713403–21111370	q11.21	22	Deletion	Supraventricular tachycardia	Heterozygote	VUS	397.97 KB	16
650	11:18278567–30302330	p15.1‐p14.1	11	Deletion	Supraventricular tachycardia	Heterozygote	Pathogenic	12.02 MB	106
651	4:71661–547305	p16.3	4	Deletion	Supraventricular tachycardia	Heterozygote	Likely pathogenic	475.64 KB	11
652	3:58342111–58475638	p14.3	3	Deletion	Supraventricular tachycardia	Heterozygote	VUS	133.53 KB	2
653	9:204193–11219608	p24.3‐p23	9	Deletion	Supraventricular tachycardia	Heterozygote	Pathogenic	11.02 MB	101
654	1:233008441–248918469	q42.2‐q22	1	Duplication	Supraventricular tachycardia	Heterozygote	Pathogenic	15.91 MB	235
655	9:3286700–4051530	p24.2	9	Duplication	Supraventricular tachycardia	Heterozygote	VUS	764.83 KB	4
656	15:52093367–52287085	q21.2	15	Deletion	Sinus bradycardia	Heterozygote	Pathogenic	193.72 KB	5
657	1:20214248–22563917	p36.12	1	Deletion	Supraventricular tachycardia	Heterozygote	Likely pathogenic	2.35 MB	53
658	22:20713403–21111370	q11.21	22	Deletion	Supraventricular tachycardia	Heterozygote	VUS	397.97 KB	16
659	11:18278567–30302330	p15.1‐p14.1	11	Deletion	Supraventricular tachycardia	Heterozygote	Pathogenic	12.02 MB	106
660	4:71661–547305	p16.3	4	Deletion	Supraventricular tachycardia	Heterozygote	Likely pathogenic	475.64 KB	11
661	3:58342111–58475638	p14.3	3	Deletion	Supraventricular tachycardia	Heterozygote	VUS	133.53 KB	2
662	9:204193–11219608	p24.3‐p23	9	Deletion	Supraventricular tachycardia	Heterozygote	Pathogenic	11.02 MB	101
663	1:233008441–248918469	q42.2‐q44	1	Duplication	Supraventricular tachycardia	Heterozygote	Pathogenic	15.91 MB	235
664	9:3286700–4051530	p24.2	9	Duplication	Supraventricular tachycardia	Heterozygote	VUS	764.83 KB	4
665	15:52093367–52287085	q21.2	15	Deletion	Sinus bradycardia	Homozygous	Pathogenic	193.72 KB	5
666	13:29522341–114317867	q12.3‐q34	13	Deletion	Abnormality of ductus venosus blood flow	Heterozygote	Pathogenic	84.80 MB	841
667	9:220253–138212068	p24.3‐q34.3	9	Duplication	Abnormality of ductus venosus blood flow	Heterozygote	Pathogenic	137.99 MB	1553
668	17:16919369–20289856	p11.2	17	Duplication	Persistent fetal circulation	Heterozygote	Pathogenic	3.37 MB	108
669	6:93926448–104047419	q16.1‐q16.3	6	Deletion	Persistent patent ductus venosus	Heterozygote	VUS	10.12 MB	44
670	16:29662633–30187279	p11.2	16	Deletion	Persistent patent ductus venosus	Heterozygote	Likely pathogenic		33
671	9:220253–138212068	p24.3‐q34.3	9	Duplication	Congenital portosystemic venous shunt	Heterozygote	Pathogenic	137.99 MB	1553
672	X:141258960–141676008	q27.2	X	Deletion	Left ventricular outflow tract obstruction	Hemizygous	VUS	417.05 KB	5
673	16:46407633–62056029	q11.2‐q21	16	Deletion	Left ventricular outflow tract obstruction	Heterozygote	Pathogenic	15.65 MB	199
674	9:27962012–28083674	p21.2‐p21.1	9	Deletion	Right ventricular failure	Heterozygote	VUS	121.66 KB	1
675	5:55905262–55925941	q11.2	5	Deletion	Livedo racemosa	Heterozygote	VUS	20.68 KB	1
676	7:65231558–65401160	q11.21	7	Deletion	Cutis marmorata	Heterozygote	VUS	169.60 KB	2
677	10:127891809–133609001	q26.2‐q26.3	10	Deletion	Cutis marmorata	Heterozygote	VUS		62
678	15:23747749–24638732	q11.2	15	Deletion	Cutis marmorata	Heterozygote	VUS	890.98 KB	4
679	X:140520186–141855335	q27.1‐q27.2	X	Copy‐Number Gain	Cutis marmorata	Heterozygote	VUS	1.34 MB	15
680	16:11847293–12521441	p13.13‐p13.12	16	Deletion	Cutis marmorata	Heterozygote	VUS	674.15 KB	9
681	10:28131504–28279784	p12.1	10	Deletion	Cutis marmorata	Heterozygote	VUS	148.28 KB	1
682	22:28248728–28547489	q12.1	22	Duplication	Cutis marmorata	Heterozygote	VUS	298.76 KB	2
683	12:47226813–47279664	q13.11	12	Deletion	Cutis marmorata	Heterozygote	VUS	52.85 KB	1
684	2:147820862–148029473	q22.3‐q23.1	2	Deletion	Cutis marmorata	Heterozygote	VUS	208.61 KB	4
685	7:5111943–6705939	p22.1	7	Deletion	Cutis marmorata	Heterozygote	VUS	1.59 MB	39
686	3:1556634–2578945	p26.3	3	Duplication	Cutis marmorata	Heterozygote	VUS	1.02 MB	7
687	17:31932306–32513506	q11.2	17	Duplication	Cutis marmorata	Heterozygote	VUS	581.20 KB	16
688	12:121448835–122111763	q24.31	12	Deletion	Cutis marmorata	Heterozygote	Likely pathogenic	662.93 KB	16
689	8:370697–6550837	p23.3‐p23.1	8	Deletion	Cutis marmorata	Heterozygote	Pathogenic	6.18 MB	26
690	8:28727550–43319892	p21.1‐p11.1	8	Duplication	Cutis marmorata	Heterozygote	Pathogenic	14.59 MB	164
691	8:142700374–144198925	q24.3	8	Duplication	Cutis marmorata	Heterozygote	VUS	1.50 MB	73
692	12:80684–14848486	p13.33‐p12.3	12	Duplication	Cutis marmorata	Heterozygote	Pathogenic	14.77 MB	334
693	17:61859823–65666019	q23.2‐q24.1	17	Duplication	Cutis marmorata	Heterozygote	Pathogenic	3.81 MB	84
694	13:83947880–84072685	q31.1	13	Deletion	Cutis marmorata	Heterozygote	VUS	124.81 KB	1
695	2:134928637–134991490	q21.3	2	Deletion	Cutis marmorata	Heterozygote	Pathogenic	62.85 KB	2
696	4:9369056–10361054	p16.1	4	Duplication	Cutis marmorata	Heterozygote	VUS	992.00 KB	26
697	1:155613266–155715711	q22	1	Duplication	Cutis marmorata	Heterozygote	VUS	102.45 KB	3
698	X:41914977–41937743	p11.4	X	Deletion	Cutis marmorata	Unknown	VUS	22.77 KB	2
699	X:136142690–156003433	q26.3‐q28	X	Copy number gain	Cutis marmorata	Unknown	VUS	19.86 MB	322
700	12:69192659–78600931	q15‐q21.2	12	Deletion	Cutis marmorata	Heterozygote	VUS	9.41 MB	74
701	15:31729530–32218662	q13.3	15	Duplication	Cutis marmorata	Heterozygote	Likely pathogenic	489.13 KB	3
702	X:153651239–154140759	q28	X	Duplication	Cutis marmorata	Hemizygous	Pathogenic	489.52 KB	28
703	22:37079460–50668400	q12.3‐q13.33	22	Deletion	Cutis marmorata	Heterozygote	Likely pathogenic	13.59 MB	302
704	7:10239–2887125	p22.3‐p22.2	7	Deletion	Cutis marmorata	Heterozygote	Likely pathogenic	2.88 MB	50
705	7:155270643–159335973	q36.3	7	Deletion	Cutis marmorata	Heterozygote	Likely pathogenic	4.07 MB	37
706	21:41106203–46699983	q22.2‐q22.3	21	Deletion	Cutis marmorata	Heterozygote	Likely pathogenic	5.59 MB	155
707	12:67419953–75519953	q15‐q21.2	12	Deletion	Cutis marmorata	Heterozygote	Likely pathogenic	8.10 MB	82
708	6:60001–15191790	p25.3‐p23	6	Deletion	Cutis marmorata	Heterozygote	Likely pathogenic	15.13 MB	149
709	16:10001–16698642	p13.3‐p13.11	16	Duplication	Cutis marmorata	Heterozygote	Likely pathogenic	16.69 MB	396
710	4:11322107–28322109	p15.33‐p15.1	4	Deletion	Cutis marmorata	Heterozygote	Likely pathogenic	17.00 MB	103
711	4:10001–4380201	p16.3	4	Deletion	Cutis marmorata	Heterozygote	Likely pathogenic	4.37 MB	94
712	16:10001–16698642	p13.3‐p13.11	16	Duplication	Cutis marmorata	Heterozygote	Likely pathogenic	16.69 MB	396
713	1:10002–7105674	p36.33‐p36.23	1	Deletion	Cutis marmorata	Heterozygote	Likely pathogenic	7.10 MB	177
714	8:60001–12798120	p23.3‐p23.1	8	Duplication	Cutis marmorata	Heterozygote	Likely pathogenic	12.74 MB	214
715	16:10778611–14481815	p13.13‐p13.12	16	Deletion	Cutis marmorata	Heterozygote	VUS	3.70 MB	43
716	6:3527076–7328347	p25.2‐p24.3	6	Deletion	Cutis marmorata	Heterozygote	Likely pathogenic	3.80 MB	36
717	1:86718710–89191178	p22.3‐p22.2	1	Deletion	Cutis marmorata	Heterozygote	Likely pathogenic	2.47 MB	23
718	1:58090676–62872765	p32.2‐p31.3	1	Deletion	Cutis marmorata	Heterozygote	Likely pathogenic	4.78 MB	42
719	7:73341271–74719860	q11.23	7	Duplication	Cutis marmorata	Heterozygote	Likely pathogenic	1.38 MB	31
720	3:185722922–198107137	q27.2‐q29	3	Deletion	Cutis marmorata	Heterozygote	Likely pathogenic	12.38 MB	197
721	10:54087–2193561	p15.3	10	Deletion	Cutis marmorata	Heterozygote	Likely pathogenic	2.14 MB	23
722	17:150208–3449956	p13.3‐p13.2	17	Deletion	Cutis marmorata	Heterozygote	Likely pathogenic	3.30 MB	88
723	17:75992325–83101964	q25.1‐q25.3	17	Duplication	Cutis marmorata	Heterozygote	Likely pathogenic	7.11 MB	200
724	13:108549652–113340664	q33.3‐q34	13	Deletion	Cutis marmorata	Heterozygote	Likely pathogenic	4.79 MB	61
725	9:10001–19910002	p24.3‐p21.3	9	Deletion	Cutis marmorata	Heterozygote	Likely pathogenic	19.90 MB	173
726	2:24880430–24959324	p23.3	2	Duplication	Cutis marmorata	Heterozygote	VUS	78.89 KB	2
727	13:96748485–97079899	q32.1	13	Duplication	Cutis marmorata	Heterozygote	VUS	331.42 KB	5
728	7:142246343–143005211	q34	7	Duplication	Cutis marmorata	Heterozygote	VUS	758.87 KB	92
729	16:15322000–28469000	p13.11‐p12.1	16	Triplication	Cutis marmorata	Heterozygote	Pathogenic	13.15 MB	181
730	16:28469000–29039000	p12.1‐p11.2	16	Duplication	Cutis marmorata	Heterozygote	Pathogenic	570.00 KB	27
731	14:44454169–44550417	q21.2	14	Deletion	Single umbilical artery	Heterozygote	VUS	96.25 KB	2
732	12:115067695–117003878	q24.21‐q24.22	12	Deletion	Single umbilical artery	Heterozygote	VUS	1.94 MB	17
733	X:155162126–155204445	q28	X	Deletion	Single umbilical artery	Heterozygote	VUS	42.32 KB	1
734	3:7573866–7751818	p26.1	3	Deletion	Single umbilical artery	Heterozygote	VUS	177.95 KB	1
735	16:21796533–22566270	p12.2	16	Deletion	Single umbilical artery	Heterozygote	VUS	769.74 KB	18
736	17:36461608–37849604	q12	17	Duplication	Single umbilical artery	Heterozygote	Likely pathogenic	1.39 MB	20
737	4:81801808–85231815	q21.22‐q21.23	4	Deletion	Single umbilical artery	Heterozygote	Pathogenic	3.43 MB	43
738	4:94698628–100508099	q22.3‐q24	4	Deletion	Single umbilical artery	Heterozygote	Pathogenic	5.81 MB	49
739	1:157103652–161185777	q23.1‐q23.3	1	Deletion	Single umbilical artery	Heterozygote	Pathogenic	4.08 MB	130
740	9:220253–138212068	p24.3‐q34.3	9	Duplication	Single umbilical artery	Heterozygote	Pathogenic	137.99 MB	1553
741	4:171939008–173040957	q34.1	4	Duplication	Single umbilical artery	Heterozygote	VUS	1.10 MB	1
742	12:129362035–131808811	q24.33	12	Deletion	Single umbilical artery	Heterozygote	Likely pathogenic	2.45 MB	22
743	9:611628–8842561	p24.3‐p24.1	9	Deletion	Single umbilical artery	Heterozygote	VUS	8.23 MB	90
744	3:9690977–10309753	p25.3	3	Duplication	Single umbilical artery	Heterozygote	Likely pathogenic	618.78 KB	34
745	6:85804332–92980180	q14.3‐q16.1	6	Deletion	Single umbilical artery	Heterozygote	VUS	7.18 MB	63
746	7:16924079–17372724	p21.1	7	Duplication	Single umbilical artery	Heterozygote	Likely pathogenic	448.65 KB	2
747	3:31131688–31481760	p23	3	Duplication	Single umbilical artery	Heterozygote	Likely pathogenic	350.07 KB	5
748	4:184573019–184825004	q35.1	4	Triplication	Single umbilical artery	Heterozygote	Likely pathogenic	251.99 KB	5
749	4:184866040–185237737	q35.1	4	Duplication	Single umbilical artery	Heterozygote	Likely pathogenic	371.70 KB	9
750	19:12312229–12608061	p13.2‐p13.13	19	Duplication	Single umbilical artery	Heterozygote	Likely pathogenic	295.83 KB	15
751	5:108178781–125671740	q21.3‐q23.2	5	Deletion	Single umbilical artery	Heterozygote	Pathogenic	17.49 MB	138
752	16:14954894–16155750	p13.11	16	Deletion	Single umbilical artery	Heterozygote	Likely pathogenic	1.20 MB	22
753	22:18932429–21086225	q11.21	22	Deletion	Single umbilical artery	Heterozygote	Pathogenic	2.15 MB	84
754	1:824382–10809098	p36.33‐p36.22	1	Deletion	Single umbilical artery	Heterozygote	Pathogenic	9.98 MB	212
755	2:39309479–39378125	p22.1	2	Deletion	Single umbilical artery	Heterozygote	VUS	68.65 KB	1
756	16:15398460–18068310	p13.11‐p12.3	16	Deletion	Single umbilical artery	Heterozygote	VUS	2.67 MB	26
757	1:205248452–209238739	q32.1‐q32.2	1	Duplication	Single umbilical artery	Heterozygote	Likely pathogenic	3.99 MB	73
758	1:66313039–87411543	p31.3‐p22.3	1	Deletion	Single umbilical artery	Heterozygote	Likely pathogenic	21.10 MB	187
759	X:152834352–153149489	q28	X	Duplication	Single umbilical artery	Heterozygote	VUS	315.14 KB	7
760	4:51519–27236489	p16.3‐p15.2	4	Trisomy	Single umbilical artery	‐	Likely pathogenic	27.18 MB	309
761	4:164440253–189975519	q32.3‐q35.2	4	Monosomy	Single umbilical artery	‐	Likely pathogenic	25.54 MB	203
762	19:55273362–56984851	q13.42‐q13.43	19	Deletion	Single umbilical artery	Heterozygote	Likely pathogenic	1.71 MB	73
763	6:154792944–170572661	q25.2‐q27	6	Deletion	Single umbilical artery	Heterozygote	VUS	15.78 MB	142
764	6:161560551–170602152	q26.q27	6	Deletion	Single umbilical artery	Heterozygote	VUS	9.04 MB	70
765	7:73307413–74725194	q11.23	7	Deletion	Single umbilical artery	Heterozygote	Pathogenic	1.42 MB	35
766	21:37055773–37401364	q22.13	21	Deletion	Single umbilical artery	Heterozygote	Pathogenic	345.59 KB	7
767	15:26494994–26885708	q12	15	Duplication	Single umbilical artery	Heterozygote	Likely pathogenic	390.71 KB	2
768	X:21999383–22596314	p22.11	X	Duplication	Single umbilical artery	Heterozygote	Likely pathogenic	596.93 KB	6
769	17:16919369–20289856	p11.2	17	Duplication	Single umbilical artery	Heterozygote	Pathogenic	3.37 MB	108
770	15:91886440–101858549	q26.1‐q26.3	15	Deletion	Single umbilical artery	Heterozygote	Pathogenic	9.97 MB	99
771	14:51777266–56423924	q22.1‐q22.3	14	Duplication	Echogenic intracardiac focus	Heterozygote	VUS	4.65 MB	53
772	22:31596389–36746187	q12.2‐q12.3	22	Deletion	Double outlet right ventricle with subaortic ventricular septal defect and pulmonary stenosis	Heterozygote	Pathogenic	5.15 MB	75
773	7:98371236–102449202	q21.3‐q22.1	7	Deletion	Double outlet right ventricle with subaortic ventricular septal defect and pulmonary stenosis	Heterozygote	Likely pathogenic	4.08 MB	137
774	X:139602445–139849294	q27.1	X	Duplication	Double outlet right ventricle with doubly committed ventricular septal defect without pulmonary stenosis	Hemizygous	VUS	246.85 KB	2
775	X:144935166–145374689	q27.3	X	Duplication	Double outlet right ventricle with doubly committed ventricular septal defect without pulmonary stenosis	Hemizygous	VUS	439.52 KB	3
776	15:30848497–31407819	q13.2‐q13.3	15	Duplication	Restrictive cardiomyopathy	Heterozygote	VUS	559.32 KB	8
777	15:55255492–55652832	q21.3	15	Duplication	Hypertrophic cardiomyopathy/ Concentric hypertrophic cardiomyopathy	Heterozygote	VUS	397.34 KB	10
778	17:1355564–1399125	p13.3	17	Duplication	Right ventricular cardiomyopathy	Heterozygote	VUS	43.56 KB	1
779	12:32821356–32824162	p11.21	12	Duplication	Right ventricular cardiomyopathy	Heterozygote	Likely pathogenic	2.81 KB	1
780	12:32821356–32824162	p11.21	12	Duplication	Right ventricular cardiomyopathy	Heterozygote	Likely pathogenic	2.81 KB	1
781	12:32821356–32824162	p11.21	12	Duplication	Right ventricular cardiomyopathy	Heterozygote	Likely pathogenic	2.81 KB	1
782	12:32896509–32896756	p11.21	12	Deletion	Right ventricular cardiomyopathy	Heterozygote	Pathogenic	248 BP	1
783	6:7567354–7586717	p24.3	6	Deletion	Right ventricular cardiomyopathy	Heterozygote	Pathogenic	19.36 KB	1
784	4:119136512–119187789	q26	4	Deletion	Right ventricular cardiomyopathy	Heterozygote	VUS	51.28 KB	1
785	1:1164293–6646766	p36.33‐p36.31	1	Deletion	Noncompaction cardiomyopathy/ Left ventricular noncompaction cardiomyopathy	Heterozygote	Pathogenic	5.48 MB	127
786	12:114354081–114753069	q24.21	12	Duplication	Noncompaction cardiomyopathy/ Left ventricular noncompaction cardiomyopathy	Heterozygote	Pathogenic	398.99 KB	6
787	1:824382–5872507	p36.33‐p36.31	1	Deletion	Noncompaction cardiomyopathy/ Left ventricular noncompaction cardiomyopathy	Heterozygote	Pathogenic	5.05 MB	120
788	1:169681158–174857597	q24.2‐q25.1	1	Duplication	Dilated cardiomyopathy	Heterozygote	VUS	5.18 MB	81
789	6:18072634–18168285	p22.3	6	Deletion	Dilated cardiomyopathy	Heterozygote	VUS	95.65 KB	3
790	13:59861973–60087678	q21.2	13	Deletion	Dilated cardiomyopathy	Heterozygote	VUS	225.71 KB	3
791	6:64322921–64586067	q12	6	Duplication	Dilated cardiomyopathy	Heterozygote	VUS	263.15 KB	1
792	22:20713403–21111370	q11.21	22	Deletion	Dilated cardiomyopathy	Heterozygote	VUS	397.97 KB	16
793	6:60358037–60572374	q11.1	6	Duplication	Dilated cardiomyopathy	Heterozygote	VUS	214.34 KB	1
794	3:151158721–151450174	q25.1	3	Deletion	Dilated cardiomyopathy	Heterozygote	Pathogenic	291.45 KB	9
795	3:107660761–108017912	q13.12	3	Duplication	Dilated cardiomyopathy	Heterozygote	VUS	357.15 KB	3
796	1:198722401–199149645	q32.1	1	Deletion	Dilated cardiomyopathy	Heterozygote	VUS	427.25 KB	7
797	1:174637351–174836898	q25.1	1	Deletion	Dilated cardiomyopathy	Heterozygote	Likely pathogenic	199.55 KB	2
798	8:13485631–14757736	p22	8	Deletion	Dilated cardiomyopathy	Heterozygote	VUS	1.27 MB	7
799	10:86574716–91433986	q23.2‐q23.32	10	Deletion	Dilated cardiomyopathy	Heterozygote	Pathogenic	4.86 MB	84
800	17:1355564–1399125	p13.3	17	Duplication	Dilated cardiomyopathy	Heterozygote	VUS	43.56 KB	1
801	3:107660761–108017912	q13.12	3	Duplication	Dilated cardiomyopathy	Heterozygote	VUS	357.15 KB	3
802	1:198722401–199149645	q32.1	1	Deletion	Dilated cardiomyopathy	Heterozygote	VUS	427.25 KB	7
803	1:174637351–174836898	q25.1	1	Deletion	Dilated cardiomyopathy	Heterozygote	Likely pathogenic	199.55 KB	2
804	8:13485631–14757736	p22	8	Deletion	Dilated cardiomyopathy	Heterozygote	VUS	1.27 MB	7
805	10:86574716–91433986	q23.2‐q23.32	10	Deletion	Dilated cardiomyopathy	Heterozygote	Pathogenic	4.86 MB	84
806	17:1355564–1399125	p13.3	17	Duplication	Dilated cardiomyopathy	Heterozygote	VUS	43.56 KB	1
807	6:7577779–7581569	p24.3	6	Deletion	Dilated cardiomyopathy	Heterozygote	Pathogenic	3.79 KB	1
808	X:31323598–31968339	p21.2‐p21.1	X	Duplication	Dilated cardiomyopathy	Heterozygote	Pathogenic	644.74 KB	2
809	15:34788096–34794830	q14	15	Duplication	Dilated cardiomyopathy	Heterozygote	VUS	6.74 KB	2
810	11:128911264–128921035	q24.3	11	Duplication	Dilated cardiomyopathy	Heterozygote	VUS	9.77 KB	1
811	5:155210847–155506673	q33.2	5	Duplication	Dilated cardiomyopathy	Heterozygote	VUS	295.83 KB	0
812	1:240080542–241247275	q43	1	Deletion	Dilated cardiomyopathy	Heterozygote	VUS	1.17 MB	9
813	10:3568840–3819992	p15.2‐p15.1	10	Deletion	Dilated cardiomyopathy	Heterozygote	VUS	251.15 KB	1
814	17:36461608–37808136	q12	17	Duplication	Dilated cardiomyopathy	Heterozygote	Likely pathogenic	1.35 MB	20
815	3:177127356–177842479	q26.32	3	Deletion	Dilated cardiomyopathy	Heterozygote	Pathogenic	715.12 KB	6
816	6:136408405–150720236	q23.3‐q25.1	6	Deletion	Dilated cardiomyopathy	Heterozygote	VUS	14.31 MB	139
817	6:139751940–151251065	q24.1‐q25.1	6	Deletion	Dilated cardiomyopathy	Heterozygote	VUS	11.50 MB	101
818	6:140683771–152501023	q24.1‐q25.2	6	Deletion	Dilated cardiomyopathy	Heterozygote	VUS	11.82 MB	110
819	6:145645950–156381320	q24.3‐q25.3	6	Deletion	Dilated cardiomyopathy	Heterozygote	Pathogenic	10.74 MB	112
820	14:103738574–106879501	q32.33	14	Deletion	Dilated cardiomyopathy	Heterozygote	Pathogenic	3.14 MB	240
821	11:119244263–135075340	q23.3‐q25	11	Duplication	Dilated cardiomyopathy	Heterozygote	Pathogenic	15.83 MB	243
822	6:148446766–152062907	q24.3‐q25.1	6	Deletion	Myocarditis	Heterozygote	Likely pathogenic	3.62 MB	57
823	3:177127356–177842479	q26.32	3	Deletion	Myocarditis	Heterozygote	Pathogenic	715.12 KB	6
824	X:1208053–1345928	p22.33	X	Duplication	Apical hypertrabeculation of the left ventricle	Heterozygote	VUS	137.88 KB	5
825	2:178559311–178753180	q31.2	2	Duplication	Left ventricular noncompaction	Heterozygote	VUS	193.87 KB	2
826	2:50287608–55594710	p16.3‐p16.1	2	Duplication	Left ventricular noncompaction	Heterozygote	VUS	5.31 MB	46
827	1:3416000–3699821	p36.32	1	Deletion	Left ventricular noncompaction	Heterozygote	VUS	283.82 KB	8
828	20:5046097–5318456	p13‐p12.3	20	Duplication	Left ventricular noncompaction	Heterozygote	VUS	272.36 KB	6
829	15:22655582–23174546	q11.2	15	Deletion	Pericardial effusion	Heterozygote	Likely pathogenic	518.97 KB	11
830	3:139496506–140095723	q23	3	Duplication	Pericardial effusion	Heterozygote	VUS	599.22 KB	7
831	3:25220–4562613	P26.3‐p26.1	3	Deletion	Pericardial effusion	Heterozygote	Pathogenic	4.54 MB	29
832	3:146823867–198118362	q24‐q29	3	Trisomy	Pericardial effusion	Heterozygote	Pathogenic	51.29 MB	625
833	9:123265569–138114463	q33.3‐q34.3	9	Duplication	Pericardial effusion	Heterozygote	Pathogenic	14.85 MB	354
834	11:129164294–134998513	q24.3‐q25	11	Deletion	Pericardial effusion	Heterozygote	Pathogenic	5.83 MB	55
835	9:117132868–118101205	q33.1	9	Duplication	Pericarditis	Heterozygote	VUS	968.34 KB	9
836	2:15804759–28198897	p24.3‐p23.2	2	Deletion	Pericarditis	Heterozygote	Pathogenic	12.39 MB	177
837	15:53499664–55483486	q21.3	15	Duplication	Congenital malformation of the left heart	Heterozygote	VUS	1.98 MB	14
838	21:16958355–18651524	q21.1	21	Duplication	Congenital malformation of the left heart	Heterozygote	VUS	1.69 MB	16
839	12:27140841–27633482	p11.23‐p11.22	12	Deletion	Congenital malformation of the left heart	Heterozygote	VUS	492.64 KB	6
840	3:42630175–47953678	p22.1‐p21.31	3	Deletion	Congenital malformation of the left heart	Heterozygote	VUS	5.32 MB	116
841	13:110147979–110165754	q34	13	Deletion	Congenital malformation of the left heart	Heterozygote	VUS	17.78 KB	1
842	6:45497175–46028585	p21.1	6	Duplication	Congenital malformation of the left heart	Heterozygote	VUS	531.41 KB	4
843	7:163010–3174614	p22.3‐p22.2	7	Deletion	Congenital malformation of the left heart	Heterozygote	VUS	3.01 MB	51
844	X:140096407–140535377	q27.1	X	Duplication	Congenital malformation of the left heart	Hemizygous	VUS	438.97 KB	3
845	5:176463495–176884937	q35.2	5	Duplication	Congenital malformation of the left heart	Heterozygote	VUS	421.44 KB	12
846	6:154722732–156940397	q25.2‐q25.3	6	Duplication	Congenital malformation of the left heart	Heterozygote	VUS	2.22 MB	13
847	11:121707908–134396138	q24.1‐q25	11	Deletion	Congenital malformation of the left heart	Heterozygote	Pathogenic	12.69 MB	202
848	7:114697164–114805914	q31.1	7	Deletion	Congenital malformation of the left heart	Heterozygote	VUS	108.75 KB	0
849	3:94328590–99315508	q11.2‐q12.1	3	Duplication	Congenital malformation of the left heart	Heterozygote	VUS	4.99 MB	49
850	7:122780212–122813613	q31.32	7	Duplication	Congenital malformation of the left heart	Heterozygote	VUS	33.40 KB	1
851	13:22992823–24365462	q12.12	13	Deletion	Congenital malformation of the left heart	Heterozygote	VUS	1.37 MB	26
852	7:73307413–74728004	q11.23	7	Deletion	Congenital malformation of the left heart	Heterozygote	Likely Pathogenic	1.42 MB	35
853	4:51819158–52988799	q12	4	Deletion	Congenital malformation of the left heart	Heterozygote	Likely pathogenic	1.17 MB	16
854	17:36459263–37890177	q12	17	Duplication	Congenital malformation of the left heart	Heterozygote	Likely pathogenic	1.43 MB	21
855	10:79891321–87105165	q22.3‐q23.2	10	Deletion	Congenital malformation of the left heart	Heterozygote	Pathogenic	7.21 MB	81
856	X:1197001–1308697	p22.33	X	Duplication	Congenital malformation of the left heart	Heterozygote	VUS	111.70 KB	4
857	X:22975373–23283624	p22.11	X	Deletion	Congenital malformation of the left heart	Heterozygote	VUS	308.25 KB	3
858	X:22969215–23312907	p22.11	X	Deletion	Congenital malformation of the left heart	Heterozygote	VUS	343.69 KB	3
859	15:30627532–39678751	q13.2‐q14	15	Deletion	Congenital malformation of the left heart	Heterozygote	Pathogenic	9.05 MB	99
860	21:44416925–46671337	q22.3	21	Deletion	Congenital malformation of the left heart	Heterozygote	VUS	2.25 MB	78
861	5:10001–18464134	p15.33‐p14.3	5	Deletion	Congenital malformation of the left heart	Heterozygote	Likely pathogenic	18.45 MB	186
862	9:10001–9010000	p24.3‐p23	9	Deletion	Congenital malformation of the left heart	Heterozygote	Likely Pathogenic	9.00 MB	105
863	10:116650499–133334830	q25.3‐q26.3	10	Deletion	Congenital malformation of the left heart	Heterozygote	Likely pathogenic	16.68 MB	180
864	11:115124070–135076622	q23.3‐q25	11	Deletion	Congenital malformation of the left heart	Heterozygote	Likely pathogenic	19.95 MB	330
865	7:138458000–159253352	q33‐q36.3	7	Deletion	Congenital malformation of the left heart	Heterozygote	Likely pathogenic	20.80 MB	391
866	6:60001–7054768	p25.3‐p25.1	6	Deletion	Congenital malformation of the left heart	Heterozygote	Likely pathogenic	6.99 MB	76
867	11:130724895–135076622	q24.3‐q25	11	Deletion	Congenital malformation of the left heart	Heterozygote	Likely pathogenic	4.35 MB	32
868	7:10239–7087129	p22.3‐p22.1	7	Deletion	Congenital malformation of the left heart	Heterozygote	Likely pathogenic	7.08 MB	119
869	7:148165260–159253352	q35‐q36.3	7	Deletion	Congenital malformation of the left heart	Heterozygote	Likely pathogenic	11.09 MB	147
870	8:60001–12798120	p23.3‐p23.1	8	Deletion	Congenital malformation of the left heart	Heterozygote	Likely pathogenic	12.74 MB	214
871	17:150208–11055958	p13.3‐p12	17	Deletion	Congenital malformation of the left heart	Heterozygote	Likely pathogenic	10.91 MB	332
872	18:10001–15410899	p11.32‐p11.1	18	Deletion	Congenital malformation of the left heart	Heterozygote	Likely pathogenic	15.40 MB	200
873	11:110624066–135076622	q23.1‐q25	11	Deletion	Congenital malformation of the left heart	Heterozygote	Likely pathogenic	24.45 MB	412
874	4:10001–11322107	p16.3‐p15.33	4	Deletion	Congenital malformation of the left heart	Heterozygote	Likely pathogenic	11.31 MB	208
875	21:30205811–46699983	q22.11‐q22.3	21	Deletion	Congenital malformation of the left heart	Heterozygote	Likely pathogenic	16.49 MB	356
876	1:84122296–106345336	p31.1‐p21.1	1	Deletion	Congenital malformation of the left heart	Heterozygote	Likely pathogenic	22.22 MB	238
877	5:93038538–110336400	q15‐q22.1	5	Duplication	Congenital malformation of the left heart	Heterozygote	Likely pathogenic	17.30 MB	124
878	22:17446408–25579479	q11.21‐q12.1	22	Deletion	Congenital malformation of the left heart	Heterozygote	Likely pathogenic	8.13 MB	332
879	22:16367189–25579479	q11.1‐q12.1	22	Triplication	Congenital malformation of the left heart	Heterozygote	Likely pathogenic	9.21 MB	372
880	17:150208–22763679	p13.3‐p11.1	17	Duplication	Congenital malformation of the left heart	Heterozygote	Likely pathogenic	22.61 MB	587
881	17:75992325–83101964	q25.1‐q25.3	17	Deletion	Congenital malformation of the left heart	Heterozygote	Likely pathogenic	7.11 MB	200
882	11:119461255–130228212	q23.3‐q24.3	11	Deletion	Congenital malformation of the left heart	Heterozygote	Likely pathogenic	10.77 MB	191
883	6:40090097–43991939	p21.2‐p21.1	6	Deletion	Congenital malformation of the left heart	Heterozygote	Likely pathogenic	3.90 MB	97
884	4:3711215–3817215	p16.3	4	Duplication	Congenital malformation of the left heart	Heterozygote	VUS	106.00 KB	6
885	1:46883711–47050641	p33	1	Deletion	Congenital malformation of the left heart	Heterozygote	VUS	166.93 KB	8
886	12:99514349–99601258	q23.1	12	Deletion	Congenital malformation of the left heart	Heterozygote	VUS	86.91 KB	1
887	X:22007061–22604873	p22.11	X	Duplication	Congenital malformation of the left heart	Hemizygous	VUS	597.81 KB	6
888	9:271257–75147265	p24.3‐q21.13	9	Trisomy	Congenital malformation of the left heart	Imbalance arising from a balanced parental rearrangement	Pathogenic	74.88 MB	666
889	14:100537637–103792487	q32.2‐q32.33	14	Duplication	Congenital malformation of the left heart	Heterozygote	VUS	3.25 MB	161
890	4:51519–27236489	p16.3‐p15.2	4	Trisomy	Congenital malformation of the left heart	Heterozygote	Likely pathogenic	27.18 MB	309
891	4:164440253–189975519	q32.3‐q35.2	4	Monosomy	Congenital malformation of the left heart	Heterozygote	Likely pathogenic	25.54 MB	203
892	1:147114096–148472223	q21.1‐q21.2	1	Duplication	Congenital malformation of the left heart	Heterozygote	Likely pathogenic	1.36 MB	39
893	16:29320029–30321210	p11.2	16	Duplication	Dilatation of the ventricular cavity	Heterozygote	VUS	1.00 MB	52
894	3:72839134–72888875	p13	3	Deletion	Dilatation of the ventricular cavity	Heterozygote	VUS	49.74 KB	4
895	18:3455704–3693638	p11.31	18	Duplication	Double inlet left ventricle	Heterozygote	VUS	237.94 KB	6
896	X:154890313–155331062	q28	X	Deletion	Right ventricular hypertrophy	Unknown	VUS	440.75 KB	14
897	2:168873339–169042705	q24.3‐q31.1	2	Deletion	Right ventricular hypertrophy	Heterozygote	VUS	169.37 KB	3
898	X:7604904–8165131	p22.31	X	Duplication	Congenital hypertrophy of left ventricle	Hemizygous	VUS	560.23 KB	4
899	1:205248452–209238739	q32.1‐q32.2	1	Duplication	Congenital hypertrophy of left ventricle	Heterozygote	Likely PATHOGENIC	3.99 MB	73
900	22:18907322–21109830	q11.21	22	Duplication	Congenital hypertrophy of left ventricle	Heterozygote	Likely pathogenic	2.20 MB	85
901	15:22646842–23220738	q11.2	15	Deletion	Subaortic ventricular septal bulge	Heterozygote	VUS	573.90 KB	13
902	8:60734933–60743189	q12.2	8	Deletion	Membranous subvalvular aortic stenosis	Heterozygote	Likely pathogenic	8.26 KB	1
903	X:7604904–8165131	p22.31	X	Duplication	Congenital hypertrophy of left ventricle	Hemizygous	VUS	560.23 KB	4
904	1:205248452–209238739	q32.1‐q32.2	1	Duplication	Congenital hypertrophy of left ventricle	Heterozygote	Likely pathogenic	3.99 MB	73
905	2:168873339–169042705	q24.3‐q31.1	2	Deletion	Right ventricular dilatation	Heterozygote	VUS	169.37 KB	3
906	17:1355564–1399125	p13.3	17	Duplication	Right ventricular cardiomyopathy	Heterozygote	VUS	43.56 KB	1
907	12:32821356–32824162	p11.21	12	Duplication	Right ventricular cardiomyopathy	Heterozygote	Likely pathogenic	2.81 KB	1
908	12:32896509–32896756	p11.21	12	Deletion	Right ventricular cardiomyopathy	Heterozygote	Pathogenic	248 BP	1
909	6:7567354–7586717	p24.3	6	Deletion	Right ventricular cardiomyopathy	Heterozygote	Pathogenic	19.36 KB	1
910	17:44843532–44855081	q21.31	17	Deletion	Hypoplasia of right ventricle	Heterozygote	Likely pathogenic	11.55 KB	2
911	20:53693529–54133284	q13.2	20	Duplication	Hypoplasia of right ventricle	Heterozygote	VUS	439.76 KB	3
912	20:56754320–57358556	q13.31	20	Duplication	Hypoplasia of right ventricle	Heterozygote	VUS	604.24 KB	8
913	8:11179395–11867556	p23.1	8	Deletion	Hypoplasia of right ventricle	Heterozygote	Likely pathogenic	688.16 KB	18
914	4:104369862–110623700	q24‐q25	4	Duplication	Hypoplasia of right ventricle	Heterozygote	Likely pathogenic	6.25 MB	75
915	4:3711215–3817215	p16.3	4	Duplication	Hypoplasia of right ventricle	Heterozygote	VUS	106.00 KB	3
916	1:46883711–47050641	p33	1	Deletion	Hypoplasia of right ventricle	Heterozygote	VUS	166.93 KB	8
917	12:99514349–99601258	q23.1	12	Deletion	Hypoplasia of right ventricle	Heterozygote	VUS	86.91 KB	1
918	X:154890313–155331062	q28	X	Deletion	Right ventricular hypertrophy	Heterozygote	VUS	440.75 KB	14
919	2:168873339–169042705	q24.3‐q31.1	2	Deletion	Right ventricular hypertrophy	Heterozygote	VUS	169.37 KB	3
920	17:79681473–83151271	q25.3	17	Duplication	Ventricular septal hypertrophy	Heterozygote	Likely pathogenic	3.47 MB	111
921	5:181100067–181285262	q35.3	5	Deletion	Ventricular septal hypertrophy	Heterozygote	Likely Pathogenic	185.20 KB	14
922	X:6489363–7902394	p22.31	X	Deletion	Perimembranous ventricular septal defect	Heterozygote	Pathogenic	1.41 MB	7
923	14:44454169–44550417	q21.2	14	Deletion	Perimembranous ventricular septal defect	Heterozygote	VUS	96.25 KB	2
924	12:115067695–117003878	q24.21‐q24.22	12	Deletion	Perimembranous ventricular septal defect	Heterozygote	VUS	1.94 MB	17
925	X:155162126–155204445	q28	X	Deletion	Perimembranous ventricular septal defect	Heterozygote	VUS	42.32 KB	1
926	15:94225382–97781641	q26.2	15	Deletion	Perimembranous ventricular septal defect	Heterozygote	VUS	3.56 MB	21
927	9:135626922–137002729	q34.3	9	Deletion	Perimembranous ventricular septal defect	Heterozygote	Likely pathogenic	1.38 MB	59
928	2:78687690–79825248	p12	2	Duplication	Perimembranous ventricular septal defect	Heterozygote	Likely pathogenic	1.14 MB	12
929	16:15310625–18535408	p13.11‐p12.3	16	Duplication	Perimembranous ventricular septal defect	Heterozygote	VUS	3.22 MB	39
930	22:18907352–21151099	q11.21	22	Deletion	Perimembranous ventricular septal defect	Heterozygote	VUS	2.24 MB	88
931	3:195844767–197572911	q29	3	Duplication	Perimembranous ventricular septal defect	Heterozygote	Likely pathogenic	1.73 MB	48
932	19:22753907–41007211	p12‐q13.2	19	Duplication	Perimembranous ventricular septal defect	Heterozygote	Likely pathogenic	18.25 MB	363
933	3:57122002–60536953	p14.3‐p14.2	3	Duplication	Perimembranous ventricular septal defect	Heterozygote	Likely pathogenic	3.41 MB	36
934	2:62135817–66126317	p15‐p14	2	Deletion	Perimembranous ventricular septal defect	Heterozygote	Pathogenic	3.99 MB	61
935	11:126487037–135068576	q24.2‐q25	11	Deletion	Perimembranous ventricular septal defect	Heterozygote	Pathogenic	8.58 MB	77
936	8:64008628–65042993	q12.3	8	Duplication	Perimembranous ventricular septal defect	Heterozygote	Likely pathogenic	1.03 MB	7
937	11:96284930–98188464	q21‐q22.1	11	Duplication	Inlet ventricular septal defect	Heterozygote	VUS	1.90 MB	11
938	2:200861402–205639850	q33.1‐q33.3	2	Duplication	Inlet ventricular septal defect	Heterozygote	VUS	4.78 MB	92
939	22:18902649–21087655	q11.21	22	Deletion	Inlet ventricular septal defect	Heterozygote	Pathogenic	2.19 MB	85
940	1:10001–6112088	p36.33‐p36.31	1	Deletion	Inlet ventricular septal defect	Heterozygote	Pathogenic	6.10 MB	153
941	3:136422538–148529216	q22.3‐q24	3	Deletion	Gerbode ventricular septal defect	Heterozygote	Pathogenic	12.11 MB	121
942	6:145343316–155645383	q24.3‐q25.3	6	Deletion	Gerbode ventricular septal defect	Heterozygote	Likely pathogenic	10.30 MB	110
943	2:94863294–102677859	q11.1‐q12.1	2	Duplication	Gerbode ventricular septal defect	Heterozygote	pathogenic	7.81 MB	143
944	22:38495688–41586395	q13.1‐q13.2	22	Duplication	Restrictive ventricular septal defect	Heterozygote	Likely pathogenic	3.09 MB	83
945	5:14380879–14463148	p15.2	5	Deletion	Non‐restrictive ventricular septal defect	Heterozygote	Pathogenic	82.27 KB	1
946	9:120762786–124425340	q33.2‐q33.3	9	Triplication	Non‐restrictive ventricular septal defect	Heterozygote	VUS	3.66 MB	63
947	10:111535658–117903475	q25.2‐q26.11	10	Deletion	Apical muscular ventricular septal defect	Heterozygote	VUS	6.37 MB	68
948	15:53499664–55483486	q21.3	15	Duplication	Hypoplastic left heart	Heterozygote	VUS	1.98 MB	14
949	21:16958355–18651524	q21.1	21	Duplication	Hypoplastic left heart	Heterozygote	VUS	1.69 MB	16
950	12:27140841–27633482	p11.23‐p11.22	12	Deletion	Hypoplastic left heart	Heterozygote	VUS	492.64 KB	6
951	3:42630175–47953678	p22.1‐p21.31	3	Deletion	Hypoplastic left heart	Heterozygote	VUS	5.32 MB	116
952	13:110147979–110165754	q34	13	Deletion	Hypoplastic left heart	Heterozygote	VUS	17.78 KB	1
953	6:45497175–46028585	p21.1	6	Duplication	Hypoplastic left heart	Heterozygote	VUS	531.41 KB	4
954	7:163010–3174614	p22.3‐p22.2	7	Deletion	Hypoplastic left heart	Heterozygote	VUS	3.01 MB	51
955	X:140096407–140535377	q27.1	X	Duplication	Hypoplastic left heart	Hemizygous	VUS	438.97 KB	3
956	5:176463495–176884937	q35.2	5	Duplication	Hypoplastic left heart	Heterozygote	VUS	421.44 KB	12
957	6:154722732–156940397	q25.2‐q25.3	6	Duplication	Hypoplastic left heart	Heterozygote	VUS	2.22 MB	13
958	11:121707908–134396138	q24.1‐q25	11	Deletion	Hypoplastic left heart	Heterozygote	Pathogenic	12.69 MB	202
959	7:114697164–114805914	q31.1	7	Deletion	Hypoplastic left heart	Heterozygote	VUS	108.75 KB	0
960	3:94328590–99315508	q11.2‐q12.1	3	Duplication	Hypoplastic left heart	Heterozygote	VUS	4.99 MB	49
961	7:122780212–122813613	q31.32	7	Duplication	Hypoplastic left heart	Heterozygote	VUS	33.40 KB	1
962	13:22992823–24365462	q12.12	13	Deletion	Hypoplastic left heart	Heterozygote	VUS	1.37 MB	26
963	7:73307413–74728004	q11.23	7	Deletion	Hypoplastic left heart	Heterozygote	Likely pathogenic	1.42 MB	35
964	17:36459263–37890177	q12	17	Duplication	Hypoplastic left heart	Heterozygote	Likely pathogenic	1.43 MB	21
965	10:79891321–87105165	q22.3‐q23.2	10	Deletion	Hypoplastic left heart	Heterozygote	Pathogenic	7.21 MB	81
966	X:1197001–1308697	p22.33	X	Duplication	Hypoplastic left heart	Heterozygote	VUS	111.70 KB	4
967	X:22975373–23283624	p22.11	X	Deletion	Hypoplastic left heart	Hemizygous	VUS	308.25 KB	3
968	X:22969215–23312907	p22.11	X	Deletion	Hypoplastic left heart	Heterozygote	VUS	343.69 KB	3
969	15:30627532–39678751	q13.2‐q14	15	Deletion	Hypoplastic left heart	Heterozygote	Pathogenic	9.05 MB	99
970	21:44416925–46671337	p22.3	21	Deletion	Hypoplastic left heart	Heterozygote	VUS	2.25 MB	78
971	5:10001–18464134	p15.33‐p14.3	5	Deletion	Hypoplastic left heart	Heterozygote	Likely pathogenic	18.45 MB	186
972	9:10001–9010000	p24.3‐p23	9	Deletion	Hypoplastic left heart	Heterozygote	Likely pathogenic	9.00 MB	105
973	10:116650499–133334830	q25.3‐q26.3	10	Deletion	Hypoplastic left heart	Heterozygote	Likely pathogenic	16.68 MB	180
974	11:115124070–135076622	q23.3‐q25	11	Deletion	Hypoplastic left heart	Heterozygote	Likely pathogenic	19.95 MB	330
975	7:138458000–159253352	q33‐q36.3	7	Deletion	Hypoplastic left heart	Heterozygote	Likely pathogenic	20.80 MB	391
976	6:60001–7054768	p25.3‐p25.1	6	Deletion	Hypoplastic left heart	Heterozygote	Likely pathogenic	6.99 MB	76
977	11:130724895–135076622	q24.3‐q25	11	Deletion	Hypoplastic left heart	Heterozygote	Likely pathogenic	4.35 MB	32
978	7:10239–7087129	p22.3‐p22.1	7	Deletion	Hypoplastic left heart	Heterozygote	Likely pathogenic	7.08 MB	119
979	7:148165260–159253352	q35‐q36.3	7	Deletion	Hypoplastic left heart	Heterozygote	Likely pathogenic	11.09 MB	147
980	8:60001–12798120	p23.3‐p23.1	8	Deletion	Hypoplastic left heart	Heterozygote	Likely pathogenic	12.74 MB	214
981	17:150208–11055958	p13.3‐p12	17	Deletion	Hypoplastic left heart	Heterozygote	Likely pathogenic	10.91 MB	332
982	18:10001–15410899	p11.32‐p11.1	18	Deletion	Hypoplastic left heart	Heterozygote	Likely pathogenic	15.40 MB	200
983	11:110624066–135076622	q23.1‐q25	11	Deletion	Hypoplastic left heart	Heterozygote	Likely pathogenic	24.45 MB	412
984	4:10001–11322107	p16.3‐p15.33	4	Deletion	Hypoplastic left heart	Heterozygote	Likely pathogenic	11.31 MB	208
985	21:30205811–46699983	q22.11‐q22.3	21	Deletion	Hypoplastic left heart	Heterozygote	Likely pathogenic	16.49 MB	356
986	1:84122296–106345336	p31.1‐p21.1	1	Deletion	Hypoplastic left heart	Heterozygote	Likely pathogenic	22.22 MB	238
987	5:93038538–110336400	q15‐q22.1	5	Duplication	Hypoplastic left heart	Heterozygote	Likely pathogenic	17.30 MB	124
988	22:17446408–25579479	q11.21‐q12.1	22	Deletion	Hypoplastic left heart	Heterozygote	Likely pathogenic	8.13 MB	332
989	22:16367189–25579479	q11.1‐q12.1	22	Triplication	Hypoplastic left heart	Heterozygote	Likely pathogenic	9.21 MB	372
990	17:150208–22763679	p13.3‐p11.1	17	Duplication	Hypoplastic left heart	Heterozygote	Likely pathogenic	22.61 MB	587
991	17:75992325–83101964	q25.1‐q25.3	17	Deletion	Hypoplastic left heart	Heterozygote	Likely pathogenic	7.11 MB	200
992	11:119461255–130228212	q23.3‐q24.3	11	Deletion	Hypoplastic left heart	Heterozygote	Likely pathogenic	10.77 MB	191
993	6:40090097–43991939	p21.2‐p21.1	6	Deletion	Hypoplastic left heart	Heterozygote	Likely pathogenic	3.90 MB	97
994	4:3711215–3817215	p16.3	4	Duplication	Hypoplastic left heart	Heterozygote	VUS	106.00 KB	2
995	1:46883711–47050641	p33	1	Deletion	Hypoplastic left heart	Heterozygote	VUS	166.93 KB	8
996	12:99514349–99601258	q23.1	12	Deletion	Hypoplastic left heart	Heterozygote	VUS	86.91 KB	1
997	X:22007061–22604873	p22.11	X	Duplication	Hypoplastic left heart	Hemizygous	VUS	597.81 KB	6
998	9:271257–75147265	p24.3‐q21.13	9	Trisomy	Hypoplastic left heart	Imbalance arising from a balanced parental rearrangement	Pathogenic	74.88 MB	666
999	14:100537637–103792487	q32.2‐q32.33	14	Duplication	Hypoplastic left heart	Heterozygote	VUS	3.25 MB	161
1000	4:51519–27236489	p16.3‐p15.2	4	Trisomy	Hypoplastic left heart	De novo (unconfirmed parentage)	VUS	27.18 MB	309
1001	4:164440253–189975519	q32.3‐q35.2	4	Monosomy	Hypoplastic left heart	Heterozygote	Likely pathogenic	25.54 MB	203
1002	1:147114096–148472223	q21.1‐q21.2	1	Duplication	Hypoplastic left heart	Heterozygote	Likely pathogenic	1.36 MB	39
1003	14:19956421–20328537	q11.2	14	Duplication	Hypoplastic right heart	Heterozygote	VUS	372.12 KB	24
1004	3:28978241–30044284	p24.1	3	Deletion	Hypoplastic right heart	Heterozygote	VUS	1.07 MB	7
1005	11:68880916–70504258	q13.3‐q13.4	11	Duplication	Hypoplastic right heart	Heterozygote	VUS	1.62 MB	33
1006	18:148963–8434247	p11.32‐p11.23	18	Deletion	Hypoplastic right heart	Heterozygote	Pathogenic	8.29 MB	84
1007	18:78964307–80252149	q23	18	Deletion	Hypoplastic right heart	Heterozygote	VUS	1.29 MB	16
1008	X:154890313–155331062	q28	X	Duplication	Situs inversus totalis	Heterozygote	VUS	440.75 KB	14
1009	20:458743–1986063	p13	20	Deletion	Situs inversus totalis	Heterozygote	Likely pathogenic	1.53 MB	33
1010	4:82171455–84826958	q21.22‐q21.23	4	Duplication	Situs inversus totalis	Heterozygote	VUS	2.66 MB	36
1011	7:10239–7087129	p22.3‐p22.1	7	Deletion	Situs inversus totalis	Heterozygote	Likely pathogenic	7.08 MB	119
1012	7:148165260–159253352	q35‐q36.3	7	Deletion	Situs inversus totalis	Heterozygote	Likely pathogenic	11.09 MB	147
1013	17:69192265–83101964	q24.3‐q25.3	17	Duplication	Situs inversus totalis	Heterozygote	Likely pathogenic	13.91 MB	310
1014	11:130724895–135076622	q24.3‐q25	11	Deletion	Situs inversus totalis	Heterozygote	Likely pathogenic	4.35 MB	32
1015	15:88455765–101898930	q25.3‐q26.3	15	Duplication	Situs inversus totalis	Heterozygote	Likely pathogenic	13.44 MB	182
1016	2:235309357–242160331	q37.2‐q37.3	2	Deletion	Situs inversus totalis	Heterozygote	Likely pathogenic	6.85 MB	107
1017	3:188671476–188811213	q28	3	Deletion	Ectopia cordis	Heterozygote	VUS	139.74 KB	2
1018	8:6285586–6421430	p23.2‐p23.1	8	Duplication	Left atrial isomerism	Heterozygote	VUS	135.84 KB	2
1019	X:154890313–155331062	q28	X	Duplication	Situs inversus totalis	Heterozygote	VUS	440.75 KB	14
1020	20:458743–1986063	p13	20	Deletion	Situs inversus totalis	Heterozygote	Likely pathogenic	1.53 MB	33
1021	17:69192265–83101964	q24.3‐q25.3	17	Duplication	Situs inversus totalis	Heterozygote	Likely pathogenic	13.91 MB	310
1022	11:130724895–135076622	q24.3‐q25	11	Duplication	Situs inversus totalis	Heterozygote	Likely pathogenic	4.35 MB	32
1023	15:88455765–101898930	q25.3‐q26.3	15	Duplication	Situs inversus totalis	Heterozygote	Likely pathogenic	13.44 MB	182
1024	2:235309357–242160331	q37.2‐q37.3	2	Deletion	Situs inversus totalis	Heterozygote	Likely pathogenic	6.85 MB	107
1025	X:6570680–8129470	p22.31	X	Duplication	Congenitally corrected transposition of the great arteries with ventricular septal defect	Heterozygote	VUS	1.56 MB	7
1026	19:16040734–17815693	p13.12‐p13.11	19	Duplication	Primum atrial septal defect	Heterozygote	VUS	1.77 MB	55
1027	7:146252855–159327017	q35‐q36.3	7	Duplication	Primum atrial septal defect	Heterozygote	Pathogenic	13.07 MB	154
1028	6:160289271–170610394	q25.3‐q27	6	Duplication	Primum atrial septal defect	Heterozygote	Pathogenic	10.32 MB	77
1029	11:27055600–27203827	p14.2‐p14.1	11	Duplication	Secundum atrial septal defect	Heterozygote	VUS	148.23 KB	2
1030	7:34211999–34424526	p14.3	7	Deletion	Secundum atrial septal defect	Heterozygote	VUS	212.53 KB	2
1031	17:30573691–31222388	q11.2	17	Duplication	Secundum atrial septal defect	Heterozygote	VUS	648.70 KB	17
1032	17:31235553–32021851	q11.2	17	Duplication	Secundum atrial septal defect	Heterozygote	VUS	786.30 KB	21
1033	X:363568–531543	p22.33	X	Duplication	Secundum atrial septal defect	Heterozygote	VUS	167.98 KB	2
1034	8:143432008–144119817	q24.3	8	Deletion	Secundum atrial septal defect	Heterozygote	VUS	687.81 KB	38
1035	3:66617625–73199155	p14.1‐p13	3	Deletion	Secundum atrial septal defect	Heterozygote	VUS	6.58 MB	55
1036	17:44875738–44883960	q21.31	17	Deletion	Secundum atrial septal defect	Heterozygote	Pathogenic	8.22 KB	1
1037	4:170223490–190036318	q33‐q35.2	4	Deletion	Secundum atrial septal defect	Heterozygote	VUS	19.81 MB	156
1038	17:36486499–38203073	q12	17	Duplication	Secundum atrial septal defect	Heterozygote	Likely pathogenic	1.72 MB	30
1039	17:16096687–22700673	p12‐p11.1	17	Deletion	Secundum atrial septal defect	Heterozygote	Pathogenic	6.60 MB	198
1040	1:233098426–245221161	q42.2‐q44	1	Duplication	Secundum atrial septal defect	Heterozygote	VUS	12.12 MB	134
1041	1:245312845–248918440	q44	1	Deletion	Secundum atrial septal defect	Heterozygote	VUS	3.61 MB	101
1042	12:27153357–27607134	p11.23‐p11.22	12	Deletion	Secundum atrial septal defect	Heterozygote	VUS	453.78 KB	6
1043	13:18445861–114344403	q11‐q34	13	Trisomy	Secundum atrial septal defect	De novo mosaic	Pathogenic	95.90 MB	1059
1044	10:101241107–101630125	q24.32	10	Deletion	Secundum atrial septal defect	Heterozygote	Likely pathogenic	389.02 KB	11
1045	X:154762806–154769457	q28	X	Duplication	Secundum atrial septal defect	Heterozygote	Likely pathogenic	6.65 KB	3
1046	17:60095339–62174207	q23.1‐q23.2	17	Deletion	Secundum atrial septal defect	Heterozygote	Likely pathogenic	2.08 MB	31
1047	17:79681473–83151271	q25.3	17	Duplication	Secundum atrial septal defect	Heterozygote	Likely pathogenic	3.47 MB	111
1048	5:181100067–181285262	q35.3	5	Deletion	Secundum atrial septal defect	Heterozygote	Likely pathogenic	185.20 KB	14
1049	X:253129–576281	p22.33	X	Duplication	Secundum atrial septal defect	Heterozygote	VUS	323.15 KB	6
1050	1:59124589–70791262	p32.1‐p31.1	1	Deletion	Secundum atrial septal defect	Heterozygote	Pathogenic	11.67 MB	128
1051	15:36502640–40724206	q14‐q15.1	15	Deletion	Secundum atrial septal defect	Heterozygote	Pathogenic	4.22 MB	52
1052	X:140427884–152932877	q27.1‐q28	X	Duplication	Secundum atrial septal defect	Hemizygous	VUS	12.50 MB	150
1053	6:139751940–151251065	q24.1‐q25.1	6	Deletion	Secundum atrial septal defect	Heterozygote	VUS	11.50 MB	101
1054	7:100640465–101419778	q22.1	7	Deletion	Secundum atrial septal defect	Heterozygote	VUS	779.31 KB	42
1055	7:101531694–101889441	q22.1	7	Deletion	Secundum atrial septal defect	Heterozygote	VUS	357.75 KB	4
1056	15:82543423–84146185	q25.2	15	Deletion	Patent foramen ovale	Heterozygote	Likely pathogenic	1.60 MB	23
1057	1:146911859–148314590	q21.1‐q21.2	1	Deletion	Patent foramen ovale	Heterozygote	VUS	1.40 MB	36
1058	1:240306763–241796025	q43	1	Deletion	Patent foramen ovale	Heterozygote	VUS	1.49 MB	14
1059	22:44606392–50739807	q13.31‐q13.33	22	Deletion	Patent foramen ovale	Heterozygote	VUS	6.13 MB	100
1060	12:45001–38059419	p13.33‐q12	12	Duplication	Patent foramen ovale	Heterozygote	Pathogenic	38.01 MB	515
1061	22:44596347–50780581	q13.31‐q13.33	22	Deletion	Patent foramen ovale	Heterozygote	Pathogenic	6.18 MB	101
1062	X:7996636–9651770	p22.31‐p22.2	X	Deletion	Patent foramen ovale	Hemizygous	Pathogenic	1.66 MB	12
1063	15:31729530–32218662	q13.3	15	Duplication	Patent foramen ovale	Heterozygote	VUS	489.13 KB	3
1064	6:160782513–170602152	q26‐q27	6	Deletion	Patent foramen ovale	Heterozygote	VUS	9.82 MB	73
1065	10:26844085–37604324	p12.1‐p11.21	10	Duplication	Patent foramen ovale	Heterozygote	Pathogenic	10.76 MB	141
1066	4:179411165–184039226	q34.3‐q35.1	4	Deletion	Patent foramen ovale	Heterozygote	VUS	4.63 MB	28
1067	6:108064200–108808231	q21	6	Duplication	Patent foramen ovale	Heterozygote	VUS	744.03 KB	13
1068	X:72428926–72912769	q13.1	X	Deletion	Patent foramen ovale	Heterozygote	Likely pathogenic	483.84 KB	12
1069	X:73010102–73193912	q13.2	X	Duplication	Patent foramen ovale	Heterozygote	Likely pathogenic	183.81 KB	2
1070	X:16652919–17076699	p22.2	X	Duplication	Patent foramen ovale	Heterozygote	VUS	423.78 KB	10
1071	15:29959656–32218662	q13.1‐q13.3	15	Deletion	Patent foramen ovale	Heterozygote	VUS	2.26 MB	40
1072	8:133426878–145039460	q24.22‐q24.3	8	Duplication	Patent foramen ovale	Heterozygote	VUS	11.61 MB	165
1073	1:240507087–247032805	q43‐q44	1	Deletion	Patent foramen ovale	Heterozygote	VUS	6.53 MB	71
1074	7:73307413–74728004	q11.23	7	Deletion	Patent foramen ovale	Heterozygote	Likely pathogenic	1.42 MB	35
1075	19:12978943–14596136	p13.13‐p13.12	19	Duplication	Patent foramen ovale	Heterozygote	VUS	1.62 MB	53
1076	16:29641678–30187279	p11.2	16	Duplication	Patent foramen ovale	Heterozygote	Pathogenic	545.60 KB	33
1077	17:202094–1975772	p13.3	17	Triplication	Patent foramen ovale	Heterozygote	Pathogenic	1.77 MB	39
1078	21:35797490–36067050	q22.12	21	Duplication	Patent foramen ovale	Heterozygote	VUS	269.56 KB	8
1079	4:71660–15968877	p16.3‐p15.32	4	Deletion	Patent foramen ovale	Heterozygote	Pathogenic	15.90 MB	241
1080	22:28076186–29013583	q12.1	22	Deletion	Patent foramen ovale	Heterozygote	VUS	937.40 KB	9
1081	9:204193–11219608	p24.3‐p23	9	Deletion	Patent foramen ovale	Heterozygote	Pathogenic	11.02 MB	101
1082	1:233008441–248918469	q42.2‐q44	1	Duplication	Patent foramen ovale	Heterozygote	Pathogenic	15.91 MB	235
1083	15:47078482–50577047	q21.1‐q21.2	15	Deletion	Patent foramen ovale	Heterozygote	Likely PATHOGENIC	3.50 MB	42
1084	7:114718063–116364773	q31.1‐q31.2	7	Duplication	Patent foramen ovale	Heterozygote	VUS	1.65 MB	9
1085	18:142096–14928855	p11.32‐p11.21	18	Duplication	Patent foramen ovale	Heterozygote	Pathogenic	14.79 MB	191
1086	1:147034756–148352079	q21.1‐q21.2	1	Deletion	Patent foramen ovale	Heterozygote	Pathogenic	1.32 MB	36
1087	X:85834323–86460905	q21.2	X	Duplication	Patent foramen ovale	Heterozygote	VUS	626.58 KB	10
1088	2:239045268–242099213	q37.3	2	Deletion	Patent foramen ovale	Heterozygote	Pathogenic	3.05 MB	60
1089	7:97963126–98710291	q21.3‐q22.1	7	Deletion	Patent foramen ovale	Heterozygote	VUS	747.17 KB	14
1090	1:199165325–202872996	q32.1	1	Deletion	Patent foramen ovale	Heterozygote	VUS	3.71 MB	66
1091	12:27153387–27607219	p11.23‐p11.22	12	Deletion	Patent foramen ovale	Heterozygote	VUS	453.83 KB	6
1092	6:148369109–150048238	q24.3‐q25.1	6	Deletion	Patent foramen ovale	Heterozygote	VUS	1.68 MB	34
1093	16:2023575–2096889	p13.3	16	Deletion	Patent foramen ovale	Heterozygote	Pathogenic	73.31 KB	6
1094	18:62564616–80252149	q21.33‐q23	18	Deletion	Patent foramen ovale	Heterozygote	Pathogenic	17.69 MB	115
1095	18:139089–283571	p11.32	18	Duplication/Triplication	Patent foramen ovale	Heterozygote	VUS	144.48 KB	3
1096	18:59300050–78353807	q21.32‐q23	18	Deletion	Patent foramen ovale	Heterozygote	Pathogenic	19.05 MB	129
1097	10:111710341–115096817	q25.2‐q25.3	10	Deletion	Patent foramen ovale	Heterozygote	Pathogenic	3.39 MB	40
1098	21:45227163–46680243	q22.3	21	Duplication	Patent foramen ovale	Heterozygote	Likely pathogenic	1.45 MB	34
1099	1:218359863–219693583	q41	1	Duplication	Patent foramen ovale	Heterozygote	VUS	1.33 MB	11
1100	6:161846895–170605878	q26‐q27	6	Deletion	Patent foramen ovale	Heterozygote	VUS	8.76 MB	70
1101	14:100873055–100990082	q32.2‐q32.31	14	Deletion	Patent foramen ovale	Heterozygote	Pathogenic	117.03 KB	48
1102	1:244031205–245453782	q44	1	Deletion	Patent foramen ovale	Heterozygote	Pathogenic	1.42 MB	20
1103	17:16919369–20289856	p11.2	17	Duplication	Patent foramen ovale	Heterozygote	Pathogenic	3.37 MB	108
1104	19:31803110–34965684	q12‐q13.11	19	Deletion	Sinus venosus atrial septal defect	Heterozygote	Pathogenic	3.16 MB	64
1105	6:84123051–87895734	q14.2‐q15	6	Deletion	Sinus venosus atrial septal defect	Heterozygote	VUS	3.77 MB	41
1106	16:28813457–29064948	p11.2	16	Duplication	Atrial septal dilatation	Heterozygote	Likely pathogenic	251.49 KB	14
1107	16:21826171–22396610	p12.2	16	Deletion	Atrial septal dilatation	Heterozygote	Likely pathogenic	570.44 KB	13
1108	17:79681473–83151271	q25.3	17	Duplication	Ventricular septal hypertrophy	Heterozygote	Likely pathogenic	3.47 MB	111
1109	5:181100067–181285262	q35.3	5	Deletion	Ventricular septal hypertrophy	Heterozygote	Likely pathogenic	185.20 KB	14
1110	14:44454169–44550417	q21.2	14	Deletion	Perimembranous ventricular septal defect	Heterozygote	VUS	96.25 KB	2
1111	12:115067695–117003878	q24.21‐q24.22	12	Deletion	Perimembranous ventricular septal defect	Heterozygote	VUS	1.94 MB	17
1112	X:155162126–155204445	q28	X	Deletion	Perimembranous ventricular septal defect	Heterozygote	VUS		1
1113	15:94225382–97781641	q26.2	15	Deletion	Perimembranous ventricular septal defect	Heterozygote	VUS	3.56 MB	21
1114	2:78687690–79825248	p12	2	Duplication	Perimembranous ventricular septal defect	Heterozygote	Likely pathogenic	1.14 MB	12
1115	16:15310625–18535408	p13.11‐p12.3	16	Duplication	Perimembranous ventricular septal defect	Heterozygote	VUS	3.22 MB	39
1116	22:18907352–21151099	q11.21	22	Deletion	Perimembranous ventricular septal defect	Heterozygote	VUS	2.24 MB	88
1117	3:195844767–197572911	q29	3	Duplication	Perimembranous ventricular septal defect	Heterozygote	Likely pathogenic	1.73 MB	48
1118	19:22753907–41007211	p12‐q13.2	19	Duplication	Perimembranous ventricular septal defect	Heterozygote	Likely pathogenic	18.25 MB	363
1119	3:57122002–60536953	p14.3‐p14.2	3	Duplication	Perimembranous ventricular septal defect	Heterozygote	Likely pathogenic	3.41 MB	36
1120	2:62135817–66126317	p15‐p14	2	Deletion	Perimembranous ventricular septal defect	Heterozygote	Pathogenic	3.99 MB	61
1121	11:126487037–135068576	q24.2‐q25	11	Deletion	Perimembranous ventricular septal defect	Heterozygote	Pathogenic	8.58 MB	77
1122	8:64008628–65042993	q12.3	8	Duplication	Perimembranous ventricular septal defect	Heterozygote	Likely pathogenic	1.03 MB	7
1123	11:96284930–98188464	q21‐q22.1	11	Duplication	Inlet ventricular septal defect	Heterozygote	VUS	1.90 MB	11
1124	2:200861402–205639850	q33.1‐q33.3	2	Duplication	Inlet ventricular septal defect	Heterozygote	VUS	4.78 MB	92
1125	1:10001–6112088	p36.33‐p36.31	1	Deletion	Inlet ventricular septal defect	Heterozygote	Pathogenic	6.10 MB	153
1126	3:136422538–148529216	q22.3‐q24	3	Deletion	Gerbode ventricular septal defect	Heterozygote	Pathogenic	12.11 MB	121
1127	6:145343316–155645383	q24.3‐q25.3	6	Deletion	Gerbode ventricular septal defect	Heterozygote	Likely pathogenic	10.30 MB	110
1128	2:94863294–102677859	q11.1‐q12.1	2	Duplication	Gerbode ventricular septal defect	Heterozygote	Pathogenic	7.81 MB	143
1129	22:38495688–41586395	q13.1‐q13.2	22	Duplication	Restrictive ventricular septal defect	Heterozygote	Likely pathogenic	3.09 MB	83
1130	5:14380879–14463148	p15.2	5	Deletion	Non‐restrictive ventricular septal defect	Heterozygote	Pathogenic	82.27 KB	1
1131	9:120762786–124425340	q33.2‐q33.3	9	Triplication	Non‐restrictive ventricular septal defect	Heterozygote	VUS	3.66 MB	63
1132	10:111535658–117903475	q25.2‐q26.11	10	Deletion	Apical muscular ventricular septal defect	Heterozygote	VUS	6.37 MB	68
1133	6:236142–490909	p25.3	6	Deletion	Complete atrioventricular canal defect	Heterozygote	VUS	254.77 KB	3
1134	8:8273108–11984392	p23.1	8	Deletion	Intermediate atrioventricular canal defect	Heterozygote	Pathogenic	3.71 MB	57
1135	5:176463495–176884937	q35.2	5	Duplication	Partial atrioventricular canal defect	Heterozygote	VUS	421.44 KB	12
1136	8:8273050–11865694	p23.1	8	Deletion	Partial atrioventricular canal defect	Heterozygote	Pathogenic	3.59 MB	53
1137	19:16040734–17815693	p13.12‐p13.11	19	Duplication	Primum atrial septal defect	Heterozygote	VUS	1.77 MB	55
1138	6:160289271–170610394	q25.3‐q27	6	Deletion	Primum atrial septal defect	Heterozygote	Pathogenic	10.32 MB	77
1139	14:52230529–57883649	q22.1‐q23.1	14	Deletion	Transposition of the great arteries	Heterozygote	VUS	5.65 MB	61
1140	3:36982520–38602038	p22.2	3	Deletion	Transposition of the great arteries	Heterozygote	VUS	1.62 MB	37
1141	1:147093177–148262736	q21	1	Deletion	Transposition of the great arteries	Heterozygote	VUS	1.17 MB	27
1142	6:118371140–119216358	q22.31	6	Duplication	Transposition of the great arteries	Heterozygote	VUS	845.22 KB	10
1143	16:7693139–7724540	p13.3	16	Deletion	Transposition of the great arteries	Heterozygote	VUS	31.40 KB	1
1144	4:10415765–10578160	p16.1	4	Duplication	Transposition of the great arteries	Heterozygote	VUS	162.40 KB	2
1145	3:46811233–50787139	p21.31‐p21.2	3	Deletion	Transposition of the great arteries	Heterozygote	Pathogenic	3.98 MB	152
1146	2:3033979–3451125	p25.3	2	Duplication	Transposition of the great arteries	Heterozygote	VUS	417.15 KB	4
1147	18:3455704–3693638	p11.31	18	Duplication	Transposition of the great arteries	Heterozygote	VUS	237.94 KB	6
1148	17:14194981–15538864	p12	17	Duplication	Transposition of the great arteries	Heterozygote	VUS	1.34 MB	18
1149	22:17215100–17429717	q11.1‐q11.21	22	Duplication	Transposition of the great arteries	Heterozygote	VUS	214.62 KB	6
1150	16:14874998–16198378	p13.11	16	Duplication	Transposition of the great arteries	Heterozygote	VUS	1.32 MB	30
1151	X:122825305–123037517	q25	X	Duplication	Transposition of the great arteries	Heterozygote	VUS	212.21 KB	0
1152	3:3446847–12070901	p26.2‐p25.2	3	Deletion	Transposition of the great arteries	Heterozygote	Pathogenic	8.62 MB	95
1153	3:243725–1595242	p26.3	3	Duplication	Transposition of the great arteries	Heterozygote	Likely pathogenic	1.35 MB	9
1154	9:2602116–2818582	p24.2	9	Deletion	Transposition of the great arteries	Heterozygote	Likely pathogenic	216.47 KB	4
1155	20:3208292–3613133	p13	20	Duplication	Transposition of the great arteries	Heterozygote	Likely pathogenic	404.84 KB	7
1156	2:130720420–131158155	q21.1	2	Duplication	Transposition of the great arteries	Heterozygote	Likely pathogenic	437.74 KB	8
1157	10:18438878–19641332	p12.31	10	Deletion	Transposition of the great arteries	Heterozygote	VUS	1.20 MB	8
1158	1:78721605–79312258	p31.1	1	Duplication	Transposition of the great arteries	Heterozygote	VUS	590.65 KB	2
1159	3:129431310–129504396	q21.3‐q22.1	3	Duplication	Transposition of the great arteries	Heterozygote	VUS	73.09 KB	2
1160	5:37115041–37331810	p13.2	5	Duplication	Transposition of the great arteries	Heterozygote	VUS	216.77 KB	7
1161	5:44639158–44866295	p12	5	Deletion	Transposition of the great arteries	Heterozygote	VUS	227.14 KB	4
1162	X:68359398–68376850	q12	X	Deletion	Transposition of the great arteries	Heterozygote	VUS	17.45 KB	1
1163	13:70018572–70142292	q21.33	13	Duplication	Transposition of the great arteries	Heterozygote	VUS	123.72 KB	2
1164	2:9484424–9608389	p25.1	2	Duplication	Transposition of the great arteries	Heterozygote	VUS	123.97 KB	3
1165	18:148963–8434247	p11.32‐p11.23	18	Deletion	Transposition of the great arteries	Heterozygote	Pathogenic	8.29 MB	84
1166	18:78964307–80252149	q23	18	Deletion	Transposition of the great arteries	Heterozygote	VUS	1.29 MB	16
1167	2:106543025–112341109	q12.2‐q14.1	2	Duplication	Transposition of the great arteries	Heterozygote	Pathogenic	5.80 MB	90
1168	7:75467141–76611451	q11.23	7	Deletion	Transposition of the great arteries	Heterozygote	Likely pathogenic	1.14 MB	29
1169	13:65041294–70828179	q21.31‐q21.33	13	Deletion	Transposition of the great arteries	Heterozygote	Likely pathogenic	5.79 MB	35
1170	6:118416465–118733633	q22.31	6	Duplication	Transposition of the great arteries	Heterozygote	Likely pathogenic	317.17 KB	4
1171	8:1179532–2053428	p23.3	8	Duplication	Transposition of the great arteries	Heterozygote	Likely pathogenic	873.90 KB	11
1172	22:31596389–36746187	q12.2‐q12.3	22	Deletion	Double outlet right ventricle with subaortic ventricular septal defect and pulmonary stenosis	Heterozygote	Pathogenic	5.15 MB	75
1173	7:98371236–102449202	q21.3‐q22.1	7	Deletion	Double outlet right ventricle with subaortic ventricular septal defect and pulmonary stenosis	Heterozygote	Likely pathogenic	4.08 MB	137
1174	X:139602445–139849294	q27.1	X	Duplication	Double outlet right ventricle with doubly committed ventricular septal defect without pulmonary stenosis	Heterozygote	VUS		2
1175	X:144935166–145374689	q27.3	X	Duplication	Double outlet right ventricle with doubly committed ventricular septal defect without pulmonary stenosis	Heterozygote	VUS	439.52 KB	3
1176	17:11693675–11705290	p12	17	Deletion	Tetralogy of Fallot with pulmonary stenosis	Heterozygote	Pathogenic	11.62 KB	1
1177	5:129398190–134608482	q23.3‐q31.1	5	Deletion	Tetralogy of Fallot with pulmonary stenosis	Heterozygote	Likely pathogenic	5.21 MB	71
1178	2:130594260–131340224	q21.1	2	Duplication	Tetralogy of Fallot with pulmonary stenosis	Heterozygote	VUS	745.97 KB	22
1179	2:136911052–146191493	q22.1‐q22.3	2	Deletion	Tetralogy of Fallot with pulmonary stenosis	Heterozygote	VUS	9.28 MB	54
1180	19:18837985–20217030	p13.11‐p12	19	Deletion	Tetralogy of Fallot with absent pulmonary valve	Heterozygote	VUS	1.38 MB	49
1181	17:20492297–20698718	p11.2	17	Deletion	Tetralogy of Fallot with absent pulmonary valve	Heterozygote	VUS		10
1182	22:18902649–21103321	q11.21	22	Deletion	Tetralogy of Fallot with absent pulmonary valve	Heterozygote	Pathogenic	2.20 MB	86
1183	8:16240338–30579491	p22‐p12	8	Deletion	Tetralogy of Fallot with atrioventricular canal defect	Heterozygote	VUS	14.34 MB	179
1184	18:3455704–3693638	p11.31	18	Duplication	Univentricular heart with absent left sided atrioventricular connection	Heterozygote	VUS	237.94 KB	6
1185	22:31596389–36746187	q12.2‐q12.3	22	Deletion	Mitral atresia	Heterozygote	Pathogenic	5.15 MB	75
1186	17:20492297–20698718	p11.2	17	Deletion	Tetralogy of Fallot with absent pulmonary valve	Heterozygote	VUS		10
1187	17:12929636–13010804	p12	17	Duplication	Pulmonary valve defects	Heterozygote	VUS	81.17 KB	2
1188	10:73250763–73252152	q22.2	10	Triplication	Pulmonary valve defects	Heterozygote	VUS	1.39 KB	2
1189	X:131613445–131916984	q26.2	X	Duplication	Pulmonary valve defects	Heterozygote	VUS	303.54 KB	5
1190	10:49460397–49839620	q11.23	10	Duplication	Pulmonary valve atresia	Heterozygote	VUS	379.22 KB	9
1191	20:53693529–54133284	q13.2	20	Duplication	Pulmonary valve atresia	Heterozygote	VUS	439.76 KB	3
1192	20:56754320–57358556	q13.31	20	Duplication	Pulmonary valve atresia	Heterozygote	VUS	604.24 KB	8
1193	7:146707917–146801857	q35	7	Deletion	Pulmonary valve atresia	Heterozygote	VUS	93.94 KB	1
1194	22:21444524–22366442	q11.21‐q11.22	22	Duplication	Pulmonary valve atresia	Heterozygote	VUS	921.92 KB	
1195	3:195933984–198235059	q29	3	Deletion	Pulmonary valve atresia	Heterozygote	Pathogenic	2.30 MB	63
1196	6:148511066–150947913	q25.1	6	Deletion	Bicuspid pulmonary valve	Heterozygote	VUS	2.44 MB	43
1197	9:136428708–138059695	q34.3	9	Deletion	Dysplastic aortic valve	Heterozygote	VUS	1.63 MB	85
1198	6:165121797–165432415	q27	6	Duplication	Dysplastic aortic valve	Heterozygote	VUS	310.62 KB	3
1199	X:10814–49724669	p22.33‐p11.23	X	Deletion	Aortic valve atresia	Heterozygote	Pathogenic	49.71 MB	542
1200	7:114697164–114805914	q31.1	7	Deletion	Aortic valve atresia	Heterozygote	VUS	108.75 KB	0
1201	4:39505462–39647341	p14	4	Deletion	Aortic valve atresia	Heterozygote	VUS	141.88 KB	5
1202	2:1–242193529	p25.3‐q37.3	2	Uniparental Isodisomy	Bicuspid aortic valve	Maternally inherited	Pathogenic	242.19 MB	2885
1203	10:101189104–101669105	q24.31‐q24.32	10	Duplication	Bicuspid aortic valve	Heterozygote	Pathogenic	480.00 KB	13
1204	11:118519419–118521531	q23.3	11	Deletion	Bicuspid aortic valve	Heterozygote	VUS	2.11 KB	2
1205	6:10384535–12272134	p24.3‐p24.1	6	Deletion	Bicuspid aortic valve	Heterozygote	VUS	1.89 MB	29
1206	11:61773887–61817001	q12.2	11	Deletion	Bicuspid aortic valve	Heterozygote	VUS	43.12 KB	7
1207	1:240306763–241796025	q43	1	Deletion	Bicuspid aortic valve	Heterozygote	VUS	1.49 MB	14
1208	X:38629478–38760055	p11.4	X	Duplication	Bicuspid aortic valve	Heterozygote	VUS	130.58 KB	1
1209	3:182535725–185194287	q26.33‐q27.2	3	Deletion	Bicuspid aortic valve	Heterozygote	VUS	2.66 MB	64
1210	13:19452055–24928760	q12.11‐q12.13	13	Deletion	Bicuspid aortic valve	Heterozygote	VUS	5.48 MB	105
1211	3:163217321–166682158	q26.1	3	Duplication	Bicuspid aortic valve	Heterozygote	VUS	3.46 MB	17
1212	7:39595149–40001945	p14.1	7	Duplication	Bicuspid aortic valve	Heterozygote	VUS	406.80 KB	10
1213	18:62157072–62337400	q21.33	18	Duplication	Bicuspid aortic valve	Heterozygote	VUS	180.33 KB	3
1214	1:1088608–2524557	p36.33‐p36.32	1	Duplication	Bicuspid aortic valve	Heterozygote	VUS	1.44 MB	67
1215	16:82173150–83629769	q23.3	16	Deletion	Bicuspid aortic valve	Heterozygote	VUS	1.46 MB	7
1216	21:39456728–40132519	q22.2	21	Duplication	Bicuspid aortic valve	Heterozygote	Pathogenic	675.79 KB	10
1217	16:46766–1324901	p13.3	16	Deletion	Bicuspid aortic valve	Heterozygote	VUS	1.28 MB	69
1218	2:237902839–242126245	q37.3	2	Duplication	Bicuspid aortic valve	Heterozygote	VUS	4.22 MB	82
1219	8:8235544–12078492	p23.1	8	Duplication	Bicuspid aortic valve	Heterozygote	Likely pathogenic	3.84 MB	63
1220	5:84265306–85573193	q14.3	5	Duplication	Bicuspid aortic valve	Heterozygote	VUS	1.31 MB	5
1221	18:77032666–80252149	q23	18	Duplication	Bicuspid aortic valve	Heterozygote	VUS	3.22 MB	22
1222	4:188003957–189995523	q35.2	4	Deletion	Bicuspid aortic valve	Heterozygote	VUS	1.99 MB	18
1223	15:22655582–23107440	q11.2	15	Deletion	Bicuspid aortic valve	Heterozygote	VUS	451.86 KB	7
1224	3:182657028–184519164	q26.33‐q27.1	3	Deletion	Bicuspid aortic valve	Heterozygote	Likely pathogenic	1.86 MB	53
1225	10:90451–1775938	p15.3	10	Deletion	Bicuspid aortic valve	Heterozygote	Pathogenic	1.69 MB	17
1226	10:1843722–12497634	p15.3‐p13	10	Duplication	Bicuspid aortic valve	Heterozygote	Pathogenic	10.65 MB	112
1227	20:14648507–14775777	p12.1	20	Deletion	Bicuspid aortic valve	Heterozygote	VUS	127.27 KB	2
1228	22:46842096–47069296	q13.31	22	Duplication	Bicuspid aortic valve	Heterozygote	VUS	227.20 KB	2
1229	22:43827591–50739836	q13.31‐q13.33	22	Deletion	Bicuspid aortic valve	Heterozygote	Pathogenic	6.91 MB	113
1230	15:22650812–23118746	q11.2	15	Deletion	Bicuspid aortic valve	Heterozygote	Likely PATHOGENIC	467.94 KB	7
1231	X:521205–813813	p22.33	X	Duplication	Bicuspid aortic valve	Heterozygote	VUS	292.61 KB	3
1232	6:149369609–151464532	q25.1	6	Deletion	Bicuspid aortic valve	Heterozygote	Pathogenic	2.09 MB	44
1233	22:18929329–21444618	q11.21	22	Deletion	Bicuspid aortic valve	Heterozygote	Pathogenic	2.52 MB	101
1234	16:32022076–33982343	p11.2	16	Duplication	Bicuspid aortic valve	Heterozygote	Pathogenic	1.96 MB	39
1235	11:107592917–107974640	q22.3	11	Duplication	Bicuspid aortic valve	Heterozygote	VUS	381.72 KB	4
1236	8:6316347–28782853	p23.1‐p21.1	8	Duplication	Bicuspid aortic valve	Heterozygote	Pathogenic	22.47 MB	351
1237	8:8273108–11984392	p23.1	8	Deletion	Bicuspid aortic valve	Heterozygote	Pathogenic	3.71 MB	57
1238	3:36988677–37242675	p22.2	3	Deletion	Bicuspid aortic valve	Heterozygote	Pathogenic	254.00 KB	8
1239	4:169902854–177580762	q33‐q34.3	4	Deletion	Bicuspid aortic valve	Heterozygote	Pathogenic	7.68 MB	53
1240	8:11475507–11993143	p23.1	8	Duplication	Bicuspid aortic valve	Heterozygote	Pathogenic	517.64 KB	12
1241	5:37188310–37518831	p13.2	5	Duplication	Bicuspid aortic valve	Heterozygote	VUS	330.52 KB	8
1242	15:60231318–60798116	q22.2	15	Duplication	Bicuspid aortic valve	Heterozygote	VUS	566.80 KB	8
1243	16:11497848–12259642	p13.13	16	Duplication	Bicuspid aortic valve	Heterozygote	VUS	761.79 KB	14
1244	11:7478504–7529406	p15.4	11	Duplication	Bicuspid aortic valve	Heterozygote	VUS	50.90 KB	3
1245	6:148446766–152062907	q24.3‐q25.1	6	Deletion	Bicuspid aortic valve	Heterozygote	Likely pathogenic	3.62 MB	57
1246	17:16858759–20316142	p11.2	17	Duplication	Bicuspid aortic valve	Heterozygote	Pathogenic	3.46 MB	109
1247	5:166501255–169064039	q34‐q35.1	5	Deletion	Bicuspid aortic valve	Heterozygote	Likely pathogenic	2.56 MB	15
1248	X:11091–4781375	p22.33‐p22.32	X	Deletion	Bicuspid aortic valve	Heterozygote	Pathogenic	4.77 MB	48
1249	2:38822999–39012145	p22.1	2	Deletion	Bicuspid aortic valve	Heterozygote	VUS	189.15 KB	7
1250	22:18907322–21447372	q11.21	22	Duplication	Bicuspid aortic valve	Heterozygote	Likely pathogenic	2.54 MB	102
1251	6:201798–3243600	p25.3‐p25.3	6	Deletion	Bicuspid aortic valve	Heterozygote	VUS	3.04 MB	39
1252	6:1366426–1860820	p25.3	6	Deletion	Bicuspid aortic valve	Heterozygote	VUS	494.39 KB	7
1253	2:206212–29746378	p25.3‐p23.2	2	Trisomy	Bicuspid aortic valve	Unknown	Pathogenic	29.54 MB	319
1254	2:233485513–242098125	q37.1‐q37.3	2	Duplication	Bicuspid aortic valve	Heterozygote	Likely pathogenic	8.61 MB	134
1255	22:18902649–21103321	q11.21	22	Deletion	Bicuspid aortic valve	Heterozygote	Pathogenic	2.20 MB	86
1256	17:16919369–20289856	p11.2	17	Duplication	Bicuspid aortic valve	Heterozygote	Pathogenic	3.37 MB	108
1257	6:139751940–151251065	q24.1‐q25.1	6	Deletion	Dysplastic tricuspid valve	Heterozygote	VUS	11.50 MB	101
1258	6:148511066–150947913	q25.1	6	Deletion	Dysplastic tricuspid valve	Heterozygote	VUS	2.44 MB	43
1259	5:119145427–119305208	q23.1	5	Duplication	Dysplastic tricuspid valve	Heterozygote	VUS	159.78 KB	3
1260	3:174473687–174592791	q26.31	3	Duplication	Dysplastic tricuspid valve	Heterozygote	Pathogenic	119.11 KB	1
1261	8:11179395–11867556	p23.1	8	Deletion	Tricuspid atresia	Heterozygote	Likely pathogenic	688.16 KB	18
1262	22:16367189–18742490	q11.1‐q11.21	22	Duplication	Tricuspid atresia	Heterozygote	VUS	2.38 MB	73
1263	15:22655582–23107440	q11.2	15	Deletion	Tricuspid atresia	Heterozygote	VUS	451.86 KB	7
1264	11:68880916–70504258	q13.3‐q13.4	11	Duplication	Tricuspid atresia	Heterozygote	VUS	1.62 MB	33
1265	6:124089456–124174014	q22.31	6	Deletion	Tricuspid atresia	Heterozygote	VUS	84.56 KB	1
1266	7:158799005–159325876	q36.3	7	Duplication	Tricuspid atresia	Heterozygote	VUS	526.87 KB	5
1267	2:226778845–228125189	q36.3	2	Deletion	Tricuspid atresia	Heterozygote	VUS	1.35 MB	27
1268	18:148963–8434247	p11.32‐p11.23	18	Deletion	Tricuspid atresia	Heterozygote	Pathogenic	8.29 MB	84
1269	18:78964307–80252149	q23	18	Deletion	Tricuspid atresia	Heterozygote	VUS	1.29 MB	16
1270	16:29662633–30187279	p11.2	16	Duplication	Hypoplastic tricuspid valve	Heterozygote	VUS	524.65 KB	33
1271	3:32034–4308818	p26.3‐p26.1	3	Deletion	Hypoplastic tricuspid valve	Heterozygote	VUS	4.28 MB	26
1272	3:4341585–6138452	p26.1	3	Duplication	Hypoplastic tricuspid valve	Heterozygote	VUS	1.80 MB	13
1273	5:69426715–69489254	q13.2	5	Deletion	Ebstein anomaly of the tricuspid valve	Heterozygote	VUS	62.54 KB	3
1274	5:171249601–173430169	q35.1‐q35.2	5	Deletion	Ebstein anomaly of the tricuspid valve	Heterozygote	VUS	2.18 MB	35
1275	2:48991894–49115665	p16.3	2	Deletion	Ebstein anomaly of the tricuspid valve	Heterozygote	VUS	123.77 KB	2
1276	Y:667378–1949345	p11.2	Y	Duplication	Ebstein anomaly of the tricuspid valve	Heterozygote	VUS	1.28 MB	14
1277	1:629055–7300965	p36.33‐p36.23	1	Deletion	Ebstein anomaly of the tricuspid valve	Heterozygote	VUS	6.67 MB	160
1278	1:824379–8017899	p36.33‐p36.23	1	Deletion	Ebstein anomaly of the tricuspid valve	Heterozygote	Pathogenic	7.19 MB	156
1279	9:137728310–137958459	q34.3	9	Duplication	Ebstein anomaly of the tricuspid valve	Heterozygote	VUS	230.15 KB	3
1280	3:1984526–2537549	p26.3	3	Duplication	Cleft anterior mitral valve leaflet	Heterozygote	VUS	553.02 KB	4
1281	20:4230917–10089701	p13‐p12.2	20	Deletion	Cleft anterior mitral valve leaflet	Heterozygote	Likely pathogenic	5.86 MB	56
1282	5:14859578–15364361	p15.2‐p15.1	5	Duplication	Cleft anterior mitral valve leaflet	Heterozygote	VUS	504.78 KB	6
1283	2:186309002–186561035	q32.1	2	Deletion	Cleft anterior mitral valve leaflet	Heterozygote	VUS	252.03 KB	4
1284	11:112300161–114231443	q23.1‐q23.2	11	Deletion	Cleft anterior mitral valve leaflet	Heterozygote	VUS	1.93 MB	25
1285	22:31596389–36746187	q12.2‐q12.3	22	Deletion	Cleft anterior mitral valve leaflet	Heterozygote	Pathogenic	5.15 MB	75
1286	1:1456616–1524663	p36.33	1	Duplication	Cardiomegaly	Heterozygote	Pathogenic	68.05 KB	3
1287	17:16096687–22700673	p12‐p11.1	17	Deletion	Cardiomegaly	Heterozygote	Pathogenic	6.60 MB	198
1288	9:267853–14260626	p24.3‐p22.3	9	Deletion	Cardiomegaly	Heterozygote	Pathogenic	13.99 MB	115
1289	18:66375383–78295297	q22.1‐q23	18	Duplication	Cardiomegaly	Heterozygote	Pathogenic	11.92 MB	72
1290	17:68954569–69541911	q24.2‐q24.3	17	Deletion	Cardiomegaly	Heterozygote	Pathogenic	587.34 KB	11
1291	X:6634671–8147112	p22.31	X	Duplication	Cardiomegaly	Heterozygote	Likely pathogenic	1.51 MB	7
1292	15:33813732–34181114	q14	15	Duplication	Cardiomegaly	Heterozygote	VUS	367.38 KB	7
1293	15:36808587–37223324	q14	15	Duplication	Cardiomegaly	Heterozygote	VUS	414.74 KB	4
1294	4:61733288–61829138	q13.1	4	Deletion	Cardiomegaly	Heterozygote	VUS	95.85 KB	1
1295	19:27240875–38217520	q11‐q13.2	19	Deletion	Cardiomegaly	Heterozygote	Likely pathogenic	10.98 MB	214
1296	22:17446408–25579479	q11.21‐q12.1	22	Deletion	Cardiomegaly	Heterozygote	Likely Pathogenic	8.13 MB	334
1297	15:88455765–101898930	q25.3‐q26.3	15	Deletion	Cardiomegaly	Heterozygote	Likely pathogenic	13.44 MB	183
1298	4:10001–4380201	p16.3	4	Deletion	Cardiomegaly	Heterozygote	Likely pathogenic	4.37 MB	94
1299	18:56681771–80256282	q21.31‐q23	18	Deletion	Cardiomegaly	Heterozygote	Likely pathogenic	23.57 MB	179
1300	4:169504119–190107927	q33‐q35.2	4	Deletion	Cardiomegaly	Heterozygote	Likely pathogenic	20.60 MB	176
1301	22:16367189–25579479	q11.1‐q12.1	22	Deletion	Cardiomegaly	Heterozygote	Likely pathogenic	9.21 MB	374
1302	1:914087–4071190	p36.33‐p36.32	1	Deletion	Cardiomegaly	Heterozygote	Likely pathogenic	3.16 MB	108
1303	18:59502100–80256949	q21.32‐q23	18	Deletion	Cardiomegaly	Heterozygote	Likely pathogenic	20.75 MB	143
1304	17:45596851–46076816	q21.31	17	Deletion	Cardiomegaly	Heterozygote	Likely pathogenic	479.97 KB	11
1305	9:10001–9010000	p24.3‐p23	9	Deletion	Cardiomegaly	Heterozygote	Likely pathogenic	9.00 MB	105
1306	2:180303122–187379822	q31.3‐q32.1	2	Deletion	Cardiomegaly	Heterozygote	Likely pathogenic	7.08 MB	50
1307	13:100056765–100380453	q32.3	13	Deletion	Cardiomegaly	Heterozygote	VUS	323.69 KB	4
1308	9:204193–43420196	p24.3‐q11	9	Trisomy	Cardiomegaly	Heterozygote	Pathogenic	43.22 MB	516
1309	X:10814–49724669	p22.33‐p11.23	X	Deletion	Endocardial fibroelastosis	Heterozygote	Pathogenic	49.71 MB	542
1310	22:19036311–21207225	q11.21	22	Duplication	Endocardial fibroelastosis	Heterozygote	Likely pathogenic	2.17 MB	85
1311	1:240306763–241796025	q43	1	Deletion	Patent foramen ovale	Heterozygote	VUS	1.49 MB	14
1312	22:44606392–50739807	q13.31‐q13.33	22	Deletion	Patent foramen ovale	Heterozygote	VUS	6.13 MB	100
1313	22:44596347–50780581	q13.31‐q13.33	22	Deletion	Patent foramen ovale	Heterozygote	Pathogenic	6.18 MB	101
1314	X:7996636–9651770	p22.31‐p22.2	X	Deletion	Patent foramen ovale	Hemizygous	Pathogenic	1.66 MB	12
1315	15:31729530–32218662	q13.3	15	Duplication	Patent foramen ovale	Heterozygote	VUS	489.13 KB	3
1316	6:160782513–170602152	q26‐q27	6	Deletion	Patent foramen ovale	Heterozygote	VUS	9.82 MB	73
1317	4:179411165–184039226	q34.3‐q35.1	4	Deletion	Patent foramen ovale	Heterozygote	VUS	4.63 MB	28
1318	6:108064200–108808231	q21	6	Duplication	Patent foramen ovale	Heterozygote	VUS	744.03 KB	13
1319	X:72428926–72912769	q13.1	X	Deletion	Patent foramen ovale	Heterozygote	Likely pathogenic	483.84 KB	12
1320	X:73010102–73193912	q13.2	X	Duplication	Patent foramen ovale	Heterozygote	Likely pathogenic	183.81 KB	2
1321	X:16652919–17076699	p22.2	X	Duplication	Patent foramen ovale	Heterozygote	VUS	423.78 KB	10
1322	15:29959656–32218662	q13.1‐q13.3	15	Deletion	Patent foramen ovale	Heterozygote	VUS	2.26 MB	40
1323	8:133426878–145039460	q24.22‐q24.3	8	Duplication	Patent foramen ovale	Heterozygote	VUS	11.61 MB	168
1324	1:240507087–247032805	q43‐q44	1	Deletion	Patent foramen ovale	Heterozygote	VUS	6.53 MB	71
1325	7:73307413–74728004	q11.23	7	Deletion	Patent foramen ovale	Heterozygote	Likely pathogenic	1.42 MB	35
1326	19:12978943–14596136	p13.13‐p13.12	19	Duplication	Patent foramen ovale	Heterozygote	VUS	1.62 MB	54
1327	16:29641678–30187279	p11.2	16	Duplication	Patent foramen ovale	Heterozygote	Pathogenic	545.60 KB	33
1328	21:35797490–36067050	q22.12	21	Duplication	Patent foramen ovale	Heterozygote	VUS	269.56 KB	8
1329	4:71660–15968877	p16.3‐p15.32	4	Deletion	Patent foramen ovale	Heterozygote	Pathogenic	15.90 MB	241
1330	22:28076186–29013583	q12.1	22	Deletion	Patent foramen ovale	Heterozygote	VUS	937.40 KB	9
1331	9:204193–11219608	p24.3‐p23	9	Deletion	Patent foramen ovale	Heterozygote	Pathogenic	11.02 MB	101
1332	1:233008441–248918469	q42.2‐q44	1	Duplication	Patent foramen ovale	Heterozygote	Pathogenic	15.91 MB	235
1333	15:47078482–50577047	q21.1‐q21.2	15	Deletion	Patent foramen ovale	Heterozygote	Likely pathogenic	3.50 MB	42
1334	7:114718063–116364773	q31.1‐q31.2	7	Duplication	Patent foramen ovale	Heterozygote	VUS	1.65 MB	9
1335	1:147034756–148352079	q21.1‐q21.2	1	Deletion	Patent foramen ovale	Heterozygote	Pathogenic	1.32 MB	36
1336	X:85834323–86460905	q21.2	X	Duplication	Patent foramen ovale	Heterozygote	VUS	626.58 KB	10
1337	2:239045268–242099213	q37.3	2	Deletion	Patent foramen ovale	Heterozygote	Pathogenic	3.05 MB	60
1338	7:97963126–98710291	q21.3‐q22.1	7	Deletion	Patent foramen ovale	Heterozygote	VUS	747.17 KB	14
1339	1:199165325–202872996	q32.1	1	Deletion	Patent foramen ovale	Heterozygote	VUS	3.71 MB	66
1340	12:27153387–27607219	p11.23‐p11.22	12	Deletion	Patent foramen ovale	Heterozygote	VUS	453.83 KB	6
1341	3:2091542–2193998	p26.3	3	Deletion	Patent foramen ovale	Heterozygote	VUS	102.46 KB	2
1342	6:148369109–150048238	q24.3‐q25.1	6	Deletion	Patent foramen ovale	Heterozygote	VUS	1.68 MB	34
1343	16:2023575–2096889	p13.3	16	Deletion	Patent foramen ovale	Heterozygote	Pathogenic	73.31 KB	6
1344	18:62564616–80252149	q21.33‐q23	18	Deletion	Patent foramen ovale	Heterozygote	Pathogenic	17.69 MB	115
1345	18:139089–283571	p11.32	18	Duplication/Triplication	Patent foramen ovale	Heterozygote	VUS	144.48 KB	3
1346	18:59300050–78353807	q21.32‐q23	18	Deletion	Patent foramen ovale	Heterozygote	Pathogenic	19.05 MB	129
1347	21:45227163–46680243	q22.3	21	Duplication	Patent foramen ovale	Heterozygote	Likely pathogenic	1.45 MB	34
1348	1:218359863–219693583	q41	1	Duplication	Patent foramen ovale	Heterozygote	VUS	1.33 MB	11
1349	6:161846895–170605878	q26‐q27	6	Deletion	Patent foramen ovale	Heterozygote	VUS	8.76 MB	70
1350	14:100873055–100990082	q32.2‐q32.31	14	Deletion	Patent foramen ovale	Heterozygote	Pathogenic	117.03 KB	48
1351	1:244031205–245453782	q44	1	Deletion	Patent foramen ovale	Heterozygote	Pathogenic	1.42 MB	20
1352	11:27055600–27203827	p14.2‐p14.1	11	Duplication	Secundum atrial septal defect	Heterozygote	VUS	148.23 KB	2
1353	7:34211999–34424526	p14.3	7	Deletion	Secundum atrial septal defect	Heterozygote	VUS	212.53 KB	2
1354	17:30573691–31222388	q11.2	17	Duplication	Secundum atrial septal defect	Heterozygote	VUS	648.70 KB	17
1355	17:31235553–32021851	q11.2	17	Duplication	Secundum atrial septal defect	Heterozygote	VUS	786.30 KB	21
1356	X:363568–531543	p22.33	X	Duplication	Secundum atrial septal defect	Heterozygote	VUS	167.98 KB	2
1357	8:143432008–144119817	q24.3	8	Deletion	Secundum atrial septal defect	Heterozygote	VUS	687.81 KB	38
1358	3:66617625–73199155	p14.1‐p13	3	Deletion	Secundum atrial septal defect	Heterozygote	VUS	6.58 MB	56
1359	17:44875738–44883960	q21.31	17	Deletion	Secundum atrial septal defect	Heterozygote	Pathogenic	8.22 KB	1
1360	4:170223490–190036318	q33‐q35.2	4	Deletion	Secundum atrial septal defect	Heterozygote	VUS	19.81 MB	156
1361	17:16096687–22700673	p12‐p11.1	17	Deletion	Secundum atrial septal defect	Heterozygote	Pathogenic	6.60 MB	198
1362	1:233098426–245221161	q42.2‐q44	1	Duplication	Secundum atrial septal defect	Heterozygote	VUS	12.12 MB	134
1363	1:245312845–248918440	q44	1	Deletion	Secundum atrial septal defect	Heterozygote	VUS	3.61 MB	101
1364	12:27153357–27607134	p11.23‐p11.22	12	Deletion	Secundum atrial septal defect	Heterozygote	VUS	453.78 KB	6
1365	10:101241107–101630125	q24.32	10	Deletion	Secundum atrial septal defect	Heterozygote	Likely pathogenic	389.02 KB	11
1366	X:154762806–154769457	q28	X	Duplication	Secundum atrial septal defect	Heterozygote	Likely pathogenic	6.65 KB	3
1367	17:60095339–62174207	q23.1‐q23.2	17	Deletion	Secundum atrial septal defect	Heterozygote	Likely pathogenic	2.08 MB	31
1368	17:79681473–83151271	q25.3	17	Duplication	Secundum atrial septal defect	Heterozygote	Likely pathogenic	3.47 MB	112
1369	5:181100067–181285262	q35.3	5	Deletion	Secundum atrial septal defect	Heterozygote	Likely pathogenic	185.20 KB	14
1370	X:253129–576281	p22.33	X	Duplication	Secundum atrial septal defect	Heterozygote	VUS	323.15 KB	6
1371	1:59124589–70791262	p32.1‐p31.1	1	Deletion	Secundum atrial septal defect	Heterozygote	Pathogenic	11.67 MB	128
1372	15:36502640–40724206	q14‐q15.1	15	Deletion	Secundum atrial septal defect	Heterozygote	Pathogenic	4.22 MB	52
1373	X:140427884–152932877	q27.1‐q28	X	Duplication	Secundum atrial septal defect	Hemizygous	VUS	12.50 MB	150
1374	7:100640465–101419778	q22.1	7	Deletion	Secundum atrial septal defect	Heterozygote	VUS	779.31 KB	42
1375	7:101531694–101889441	q22.1	7	Deletion	Secundum atrial septal defect	Heterozygote	VUS	357.75 KB	4
1376	6:160289271–170610394	q25.3‐q27	6	Deletion	Primum atrial septal defect	Heterozygote	Pathogenic	10.32 MB	77
1377	19:31803110–34965684	q12‐q13.11	19	Deletion	Sinus venosus atrial septal defect	Heterozygote	Pathogenic	3.16 MB	65
1378	6:84123051–87895734	q14.2‐q15	6	Deletion	Sinus venosus atrial septal defect	Heterozygote	VUS	3.77 MB	41
1379	X:65252922–65505431	q11.2‐q12	X	Deletion	Hypoplastic left atrium	Heterozygote	VUS	252.51 KB	7
1380	2:180303122–187379822	q31.3‐q32.1	2	Deletion	Right atrial enlargement	Heterozygote	Likely pathogenic	7.08 MB	50
1381	21:45227163–46680243	q22.3	21	Duplication	Right atrial enlargement	Heterozygote	Likely pathogenic	1.45 MB	34
1382	8:6285586–6421430	p23.2‐p23.1	8	Duplication	Left atrial isomerism	Heterozygote	VUS	135.84 KB	2
1383	8:6285586–6421430	p23.2‐p23.1	8	Duplication	Common atrium	Heterozygote	VUS	135.84 KB	2
1384	16:2007946–2178034	p13.3	16	Deletion	Cardiac rhabdomyoma	Heterozygote	Pathogenic	170.09 KB	17
1385	16:2023575–2096889	p13.3	16	Deletion	Cardiac rhabdomyoma	Heterozygote	Pathogenic	73.31 KB	6
1386	16:2047967–2072363	p13.3	16	Deletion	Cardiac rhabdomyoma	Heterozygote	Pathogenic	24.40 KB	1
1387	6:203288–11735845	p25.3‐p24.1	6	Duplication	Moyamoya phenomenon	Heterozygote	Pathogenic	11.53 MB	120
1388	15:31719559–32225000	q13.3	15	Duplication	Moyamoya phenomenon	Heterozygote	VUS	505.44 KB	3
1389	X:92315173–92782657	q21.31‐q21.32	X	Duplication	Moyamoya phenomenon	Hemizygous	VUS	467.49 KB	5
1390	2:158284592–159412409	q24.1‐q24.2	2	Duplication	Moyamoya phenomenon	Hemizygous	VUS	1.13 MB	18
1391	19:5850238–6958801	p13.3‐p13.2	19	Deletion	Moyamoya phenomenon	Heterozygote	VUS	1.11 MB	37
1392	1:61234758–61618373	p31.3	1	Duplication	Moyamoya phenomenon	Heterozygote	VUS	383.62 KB	2
1393	X:140427884–152932877	q27.1‐q28	X	Duplication	Moyamoya phenomenon	Hemizygous	VUS	12.50 MB	50
1394	X:38627546–38775361	p11.4	X	Duplication	Moyamoya phenomenon	Hemizygous	VUS	147.82 KB	1
1395	X:154780027–154892325	q28	X	Duplication	Moyamoya phenomenon	Hemizygous	VUS	112.30 KB	7
1396	X:154892345–155101708	q28	X	Deletion	Moyamoya phenomenon	Hemizygous	Pathogenic	209.36 KB	7
1397	1:10484930–11320061	p36.22	1	Deletion	Moyamoya phenomenon	Heterozygote	VUS	835.13 KB	19
1398	20:61446729–61504543	q13.33	20	Deletion	Moyamoya phenomenon	Heterozygote	VUS	57.81 KB	1
1399	19:43342117–43421138	q13.31	19	Deletion	Moyamoya phenomenon	Heterozygote	VUS	79.02 KB	3
1400	4:84931314–84951502	q21.23	4	Deletion	Moyamoya phenomenon	Heterozygote	VUS	20.19 KB	1
1401	2:61970742–62001045	p15	2	Deletion	Moyamoya phenomenon	Heterozygote	VUS	30.30 KB	1
1402	16:29568699–30167085	p11.2	16	Deletion	Moyamoya phenomenon	Heterozygote	Pathogenic	598.39 KB	35
1403	1:145430996–148459811	q21.1‐q21.2	1	Triplication	Moyamoya phenomenon	Heterozygote	VUS	3.03 MB	79
1404	1:240644005–240984736	q43	1	Duplication	Moyamoya phenomenon	Heterozygote	VUS	340.73 KB	5
1405	20:2618474–2656211	p13	20	Duplication	Moyamoya phenomenon	Heterozygote	VUS	37.74 KB	6
1406	17:31527773–31557657	q11.2	17	Deletion	Moyamoya phenomenon	Heterozygote	VUS	29.89 KB	2
1407	4:126938401–128222207	q28.1‐q28.2	4	Duplication	Moyamoya phenomenon	Heterozygote	VUS	1.28 MB	9
1408	4:8200483–8365627	p16.1	4	Deletion	Moyamoya phenomenon	Heterozygote	VUS	165.15 KB	3
1409	19:50818134–50842312	q13.33	19	Deletion	Moyamoya phenomenon	Heterozygote	VUS	24.18 KB	3
1410	5:103542442–103804125	q21.2	5	Duplication	Moyamoya phenomenon	Heterozygote	VUS	261.68 KB	1
1411	5:55905262–55925941	q11.2	5	Deletion	Moyamoya phenomenon	Heterozygote	VUS	20.68 KB	1
1412	17:50752214–50898234	q21.33	17	Duplication	Moyamoya phenomenon	Heterozygote	VUS	146.02 KB	7
1413	20:24603541–24796541	p11.21	20	Duplication	Moyamoya phenomenon	Heterozygote	VUS	193.00 KB	1
1414	11:85692211–85836285	q14.1	11	Deletion	Moyamoya phenomenon	Heterozygote	VUS	144.07 KB	1
1415	21:14536929–14560690	q11.2	21	Duplication	Moyamoya phenomenon	Heterozygote	VUS	23.76 KB	1
1416	13:19279154–19491233	q12.11	13	Duplication	Moyamoya phenomenon	Heterozygote	VUS	212.08 KB	8
1417	4:70302145–70415837	q13.3	4	Deletion	Moyamoya phenomenon	Heterozygote	VUS	113.69 KB	4
1418	12:82946519–83184053	q21.31	12	Deletion	Moyamoya phenomenon	Heterozygote	VUS	237.53 KB	3
1419	2:196496809–196676831	q32.3‐q33.1	2	Duplication	Moyamoya phenomenon	Heterozygote	VUS	180.02 KB	2
1420	3:131356672–131379543	q22.1	3	Deletion	Moyamoya phenomenon	Heterozygote	VUS	22.87 KB	2
1421	7:73308984–74169373	q11.23	7	Deletion	Moyamoya phenomenon	Heterozygote	Pathogenic	860.39 KB	25
1422	4:3223886–3320674	p16.3	4	Duplication	Moyamoya phenomenon	Heterozygote	VUS	96.79 KB	3
1423	8:143898499–144017667	q24.3	8	Duplication	Moyamoya phenomenon	Heterozygote	VUS	119.17 KB	5
1424	5:109639184–109731149	q21.3	5	Deletion	Moyamoya phenomenon	Heterozygote	VUS	91.97 KB	3
1425	2:130047737–130116902	q21.1	2	Duplication	Moyamoya phenomenon	Heterozygote	VUS	69.17 KB	4
1426	21:8522361–46699983	p11.2‐q22.3	21	Trisomy	Moyamoya phenomenon	Heterozygote	Pathogenic	38.18 MB	539
1427	11:62176345–62314297	q12.3	11	Duplication	Moyamoya phenomenon	Heterozygote	VUS	137.95 KB	5
1428	13:112763158–112858992	q34	13	Duplication	Moyamoya phenomenon	Heterozygote	VUS	95.83 KB	1
1429	6:160611701–160647922	q26	6	Deletion	Moyamoya phenomenon	Heterozygote	VUS	36.22 KB	1
1430	1:65207867–65246299	p31.3	1	Deletion	Moyamoya phenomenon	Heterozygote	VUS	38.43 KB	1
1431	20:49098847–49161369	q13.13	20	Duplication	Moyamoya phenomenon	Heterozygote	VUS	62.52 KB	2
1432	1:145601946–146052881	q21.1	1	Deletion	Moyamoya phenomenon	Heterozygote	VUS	450.94 KB	21
1433	1:147927436–148001589	q21.2	1	Deletion	Moyamoya phenomenon	Heterozygote	VUS	74.15 KB	2
1434	X:138578744–138615283	q26.3	X	Duplication	Moyamoya phenomenon	Heterozygote	VUS	36.54 KB	1
1435	21:8522361–46699983	p11.2‐q22.3	21	Trisomy	Moyamoya phenomenon	Heterozygote	Pathogenic	38.18 MB	539
1436	21:8522361–46699983	p11.2‐q22.3	21	Trisomy	Moyamoya phenomenon	Unknown	Pathogenic	38.18 MB	539
1437	2:63862264–63956717	p15‐p14	2	Deletion	Moyamoya phenomenon	Heterozygote	VUS	94.45 KB	2
1438	19:53042501–53219888	q13.41‐q13.42	19	Deletion	Transient ischemic attack	Heterozygote	VUS	177.39 KB	6
1439	13:40782715–64043738	q14.11‐q21.31	13	Deletion	Transient ischemic attack	Heterozygote	Pathogenic	23.26 MB	280
1440	21:32885837–33915909	q22.11	21	Deletion	Stroke‐like episode	Heterozygote	Pathogenic	1.03 MB	24
1441	15:22693148–23107440	q11.2	15	Deletion	Stroke‐like episode	Heterozygote	VUS	414.29 KB	6
1442	10:133134279–133327617	q26.3	10	Duplication	Stroke‐like episode	Heterozygote	VUS	193.34 KB	11
1443	22:20721287–21109830	q11.21	22	Duplication	Dilation of Virchow–Robin spaces	Heterozygote	VUS	388.54 KB	16
1444	16:29610177–30252740	p11.2	16	Deletion	Dilation of Virchow–Robin spaces	Heterozygote	VUS	642.56 KB	40
1445	5:176010844–177995759	q35.2‐q35.3	5	Deletion	Dilation of Virchow–Robin spaces	Heterozygote	Pathogenic	1.98 MB	52
1446	17:66053767–67715288	q24.1‐q24.2	17	Deletion	Dilation of Virchow–Robin spaces	Heterozygote	Pathogenic	1.66 MB	23
1447	6:156974–6337823	p25.3‐p25.1	6	Deletion	Dilation of Virchow–Robin spaces	Heterozygote	VUS	6.18 MB	67
1448	12:102866106–102873707	q23.2	12	Deletion	Dilation of Virchow–Robin spaces	Heterozygote	VUS	7.60 KB	1
1449	6:1366426–1860820	p25.3	6	Deletion	Dilation of Virchow–Robin spaces	Heterozygote	VUS	494.39 KB	7
1450	6:60001–34106686	p25.3‐p21.31	6	Uniparental Isodisomy	Cerebral arteriovenous malformation	Heterozygote	VUS	34.05 MB	738
1451	22:18949869–21111371	q11.21	22	Duplication	Cerebral arteriovenous malformation	Heterozygote	Likely pathogenic	2.16 MB	84
1452	13:94800976–99909036	q32.1‐q32.3	13	Deletion	Cerebral arteriovenous malformation	Heterozygote	Likely pathogenic	5.11 MB	70
1453	5:9846888–15046891	p15.31‐p15.1	5	Deletion	Cerebral arteriovenous malformation	Heterozygote	Likely pathogenic	5.20 MB	38
1454	7:10239–7087129	p22.3‐p22.1	7	Duplication	Cerebral arteriovenous malformation	Heterozygote	Likely pathogenic	7.08 MB	120
1455	7:148165260–159253352	q35‐q36.3	7	Deletion	Cerebral arteriovenous malformation	Heterozygote	Likely pathogenic	11.09 MB	147
1456	5:86635588–88065215	q14.3	5	Deletion	Vein of Galen aneurysmal malformation	Heterozygote	Pathogenic	1.43 MB	3
1457	2:220677938–225464751	q35‐q36.3	2	Deletion	Frontal venous angioma	Heterozygote	Pathogenic	4.79 MB	37
1458	16:15037866–16155750	p13.11	16	Duplication	Grade I preterm intraventricular haemorrhage	Heterozygote	Likely pathogenic	1.12 MB	21
1459	9:33414186–39156957	p13.3‐p12	9	Deletion	Grade I preterm intraventricular haemorrhage	Heterozygote	Pathogenic	5.74 MB	166
1460	4:8225924–8336806	p16.1	4	Deletion	Subdural haemorrhage	Heterozygote	VUS	110.88 KB	3
1461	22:28248728–28547489	q12.1	22	Duplication	Subdural haemorrhage	Heterozygote	VUS	298.76 KB	2
1462	X:18490854–18605032	p22.13	X	Duplication	Subdural haemorrhage	Heterozygote	Likely pathogenic	114.18 KB	2
1463	X:111196251–123377432	q23‐q25	X	Duplication	Subdural haemorrhage	Heterozygote	VUS	12.18 MB	153
1464	8:2266819–12798120	p23.3‐p23.1	8	Deletion	Renal artery stenosis	Heterozygote	Likely pathogenic	10.53 MB	193
1465	5:109639184–109731149	q21.3	5	Deletion	Renal artery stenosis	Heterozygote	VUS	91.97 KB	3
1466	4:157582591–159508262	q32.1	4	Duplication	Coronary artery atherosclerosis	Heterozygote	VUS	1.93 MB	19
1467	7:148558445–153663369	q36.1‐q36.2	7	Deletion	Peripheral arterial stenosis	Heterozygote	VUS	5.10 MB	101
1468	16:85078192–86652805	q24.1	16	Deletion	Peripheral arterial stenosis	Heterozygote	VUS	1.57 MB	28
1469	6:144123224–152644976	q24.2‐q25.2	6	Deletion	Peripheral arterial stenosis	Heterozygote	VUS	8.52 MB	84
1470	20:17871356–25871364	p12.1‐p11.1	20	Duplication	Peripheral arterial stenosis	Heterozygote	Likely pathogenic	8.00 MB	135
1471	22:17446408–25579479	q11.21‐q12.1	22	Deletion	Peripheral arterial stenosis	Heterozygote	Likely pathogenic	8.13 MB	334
1472	12:105448092–106361092	q23.3	12	Deletion	Peripheral arterial stenosis	Heterozygote	VUS	913.00 KB	7
1473	5:42493685–42700118	p13.1‐p12	5	Duplication	Aortic root aneurysm	Heterozygote	VUS	206.43 KB	1
1474	3:44932333–45290346	p21.31	3	Duplication	Aortic root aneurysm	Heterozygote	VUS	358.01 KB	8
1475	7:153837582–153975582	q36.2	7	Deletion	Aortic root aneurysm	Heterozygote	VUS	138.00 KB	1
1476	1:155215838–155231152	q22	1	Deletion	Aortic root aneurysm	Heterozygote	VUS	15.31 KB	2
1477	5:117080324–122405884	q23.1‐q23.2	5	Deletion	Aortic root aneurysm	Heterozygote	Likely pathogenic	5.33 MB	37
1478	4:85100449–85482699	q21.23	4	Deletion	Abdominal aortic aneurysm	Heterozygote	VUS	382.25 KB	2
1479	19:504595–786609	p13.3	19	Deletion	Aortic dissection	Heterozygote	VUS	282.01 KB	17
1480	16:14881435–16223192	p13.11	16	Duplication	Aortic dissection	Heterozygote	Pathogenic	1.34 MB	30
1481	11:134337919–134716294	q25	11	Deletion	Coarctation of abdominal aorta	Heterozygote	VUS	378.38 KB	4
1482	5:113461–8139878	p15.33‐p15.31	5	Deletion	Juxtaductal coarctation of the aorta	Heterozygote	Pathogenic	8.03 MB	85
1483	18:77032666–80252149	q23	18	Duplication	Preductal coarctation of the aorta	Heterozygote	VUS	3.22 MB	22
1484	4:188003957–189995523	q35.2	4	Deletion	Preductal coarctation of the aorta	Heterozygote	VUS	1.99 MB	18
1485	4:91502611–92513250	q22.1	4	Duplication	Hypoplastic aortic arch	Heterozygote	VUS	1.01 MB	5
1486	9:135337654–136026399	q34.3	9	Deletion	Hypoplastic aortic arch	Heterozygote	Likely pathogenic	688.75 KB	19
1487	1:147264477–147605870	q21.1‐q21.2	1	Duplication	Hypoplastic aortic arch	Heterozygote	VUS	341.39 KB	6
1488	2:148456951–149333909	q23.1‐q23.2	2	Deletion	Hypoplastic aortic arch	Heterozygote	Pathogenic	876.96 KB	13
1489	20:80928–19416693	p13‐p11.23	20	Deletion	Hypoplastic aortic arch	Heterozygote	Likely pathogenic	19.34 MB	261
1490	22:18162023–21444618	q11.21	22	Deletion	Vascular ring	Heterozygote	Pathogenic	3.28 MB	125
1491	22:17446408–25579479	q11.21‐q12.1	22	Deletion	Vascular ring	Heterozygote	Likely Pathogenic	8.13 MB	334
1492	6:198357–401084	p25.3	6	Deletion	Right aortic arch with mirror image branching	Heterozygote	VUS	202.73 KB	2
1493	1:1688214–1798824	p36.33	1	Deletion	Right aortic arch with mirror image branching	Heterozygote	VUS	110.61 KB	6
1494	19:8786994–8861171	p13.2	19	Duplication	Right aortic arch with mirror image branching	Heterozygote	VUS	74.18 KB	3
1495	22:19108265–19940567	q11.21	22	Duplication	Right aortic arch with mirror image branching	Heterozygote	VUS	832.30 KB	25
1496	10:46157934–46318429	q11.22	10	Deletion	Right aortic arch with mirror image branching	Heterozygote	VUS	160.50 KB	2
1497	19:54705633–54880532	q13.42	19	Duplication	Right aortic arch with mirror image branching	Heterozygote	VUS	174.90 KB	12
1498	X:154094244–154323657	q28	X	Copy‐Number Gain	Right aortic arch with mirror image branching	Heterozygote	VUS	229.41 KB	10
1499	6:366332–771504	p25.3	6	Deletion	Right aortic arch with mirror image branching	Heterozygote	VUS	405.17 KB	3
1500	22:16413886–20208946	q11.1‐q11.21	22	Deletion	Right aortic arch with mirror image branching	Heterozygote	VUS	3.80 MB	125
1501	22:23354115–24601056	q11.23	22	Duplication	Left aortic arch with retroesophageal right subclavian artery	Heterozygote	Likely pathogenic	1.25 MB	44
1502	12:20858143–21238994	p12.2‐p12.1	12	Deletion	Aorto‐right ventricular tunnel	Heterozygote	VUS	380.85 KB	4
1503	7:88615564–90236479	q21.13	7	Duplication	Aortic tortuosity	Heterozygote	VUS	1.62 MB	8
1504	22:28076186–29013583	q12.1	22	Deletion	Aortopulmonary collateral arteries	Heterozygote	VUS	937.40 KB	9
1505	22:19036311–21207225	q11.21	22	Duplication	Ascending aorta hypoplasia	Heterozygote	Likely pathogenic	2.17 MB	85
1506	22:18907322–21109830	q11.21	22	Duplication	Ascending aorta hypoplasia	Heterozygote	Likely pathogenic	2.20 MB	86
1507	4:157582591–159508262	q32.1	4	Duplication	Coronary artery atherosclerosis	Heterozygote	VUS	1.93 MB	19
1508	13:40782715–64043738	q14.11‐q21.31	13	Deletion	Coronary artery atherosclerosis	Heterozygote	Pathogenic	23.26 MB	280
1509	5:55905262–55925941	q11.2	5	Deletion	Arterial fibromuscular dysplasia	Heterozygote	VUS	20.68 KB	1
1510	21:32885837–33915909	q22.11	21	Deletion	Internal carotid artery hypoplasia	Heterozygote	Pathogenic	1.03 MB	24
1511	4:157582591–159508262	q32.1	4	Duplication	Coronary artery atherosclerosis	Heterozygote	VUS	1.93 MB	19
1512	8:2266819–12798120	p23.3‐p23.1	8	Deletion	Renal artery stenosis	Heterozygote	Likely pathogenic	10.53 MB	193
1513	5:109639184–109731149	q21.3	5	Deletion	Renal artery stenosis	Heterozygote	VUS	91.97 KB	3
1514	6:203288–11735845	p25.3‐p24.1	6	Duplication	Moyamoya phenomenon	Heterozygote	Pathogenic	11.53 MB	120
1515	15:31719559–32225000	q13.3	15	Duplication	Moyamoya phenomenon	Heterozygote	VUS	505.44 KB	3
1516	X:92315173–92782657	q21.31‐q21.32	X	Duplication	Moyamoya phenomenon	Heterozygote	VUS	467.49 KB	5
1517	2:158284592–159412409	q24.1‐q24.2	2	Duplication	Moyamoya phenomenon	Heterozygote	VUS	1.13 MB	18
1518	X:38627546–38775361	p11.4	X	Duplication	Moyamoya phenomenon	Heterozygote	VUS	147.82 KB	1
1519	X:154780027–154892325	q28	X	Duplication	Moyamoya phenomenon	Heterozygote	VUS	112.30 KB	7
1520	X:154892345–155101708	q28	X	Deletion	Moyamoya phenomenon	Heterozygote	Pathogenic	209.36 KB	7
1521	1:10484930–11320061	p36.22	1	Deletion	Moyamoya phenomenon	Heterozygote	VUS	835.13 KB	19
1522	20:61446729–61504543	q13.33	20	Deletion	Moyamoya phenomenon	Heterozygote	VUS	57.81 KB	1
1523	19:43342117–43421138	q13.31	19	Deletion	Moyamoya phenomenon	Heterozygote	VUS	79.02 KB	3
1524	4:84931314–84951502	q21.23	4	Deletion	Moyamoya phenomenon	Heterozygote	VUS	20.19 KB	1
1525	2:61970742–62001045	p15	2	Deletion	Moyamoya phenomenon	Heterozygote	VUS	30.30 KB	1
1526	16:29568699–30167085	p11.2	16	Deletion	Moyamoya phenomenon	Heterozygote	Pathogenic	598.39 KB	35
1527	1:240644005–240984736	q43	1	Duplication	Moyamoya phenomenon	Heterozygote	VUS	340.73 KB	5
1528	20:2618474–2656211	p13	20	Duplication	Moyamoya phenomenon	Heterozygote	VUS	37.74 KB	6
1529	17:31527773–31557657	q11.2	17	Deletion	Moyamoya phenomenon	Heterozygote	VUS	29.89 KB	2
1530	4:8200483–8365627	p16.1	4	Deletion	Moyamoya phenomenon	Heterozygote	VUS	165.15 KB	3
1531	19:50818134–50842312	q13.33	19	Deletion	Moyamoya phenomenon	Heterozygote	VUS	24.18 KB	3
1532	5:103542442–103804125	q21.2	5	Duplication	Moyamoya phenomenon	Heterozygote	VUS	261.68 KB	1
1533	5:55905262–55925941	q11.2	5	Deletion	Moyamoya phenomenon	Heterozygote	VUS	20.68 KB	1
1534	17:50752214–50898234	q21.33	17	Duplication	Moyamoya phenomenon	Heterozygote	VUS	146.02 KB	7
1535	20:24603541–24796541	p11.21	20	Duplication	Moyamoya phenomenon	Heterozygote	VUS	193.00 KB	1
1536	11:85692211–85836285	q14.1	11	Deletion	Moyamoya phenomenon	Heterozygote	VUS	144.07 KB	1
1537	21:14536929–14560690	q11.2	21	Duplication	Moyamoya phenomenon	Heterozygote	VUS	23.76 KB	1
1538	13:19279154–19491233	q12.11	13	Duplication	Moyamoya phenomenon	Heterozygote	VUS	212.08 KB	8
1539	4:70302145–70415837	q13.3	4	Deletion	Moyamoya phenomenon	Heterozygote	VUS	113.69 KB	4
1540	12:82946519–83184053	q21.31	12	Deletion	Moyamoya phenomenon	Heterozygote	VUS	237.53 KB	3
1541	2:196496809–196676831	q32.3‐q33.1	2	Duplication	Moyamoya phenomenon	Heterozygote	VUS	180.02 KB	2
1542	3:131356672–131379543	q22.1	3	Deletion	Moyamoya phenomenon	Heterozygote	VUS	22.87 KB	2
1543	4:3223886–3320674	p16.3	4	Duplication	Moyamoya phenomenon	Heterozygote	VUS	96.79 KB	3
1544	8:143898499–144017667	q24.3	8	Duplication	Moyamoya phenomenon	Heterozygote	VUS	119.17 KB	5
1545	5:109639184–109731149	q21.3	5	Deletion	Moyamoya phenomenon	Heterozygote	VUS	91.97 KB	3
1546	2:130047737–130116902	q21.1	2	Duplication	Moyamoya phenomenon	Heterozygote	VUS	69.17 KB	4
1547	21:8522361–46699983	p11.2‐q22.3	21	Trisomy	Moyamoya phenomenon	Heterozygote	Pathogenic	38.18 MB	539
1548	11:62176345–62314297	q12.3	11	Duplication	Moyamoya phenomenon	Heterozygote	VUS	137.95 KB	5
1549	13:112763158–112858992	q34	13	Duplication	Moyamoya phenomenon	Heterozygote	VUS	95.83 KB	1
1550	6:160611701–160647922	q26	6	Deletion	Moyamoya phenomenon	Heterozygote	VUS	36.22 KB	1
1551	1:65207867–65246299	p31.3	1	Deletion	Moyamoya phenomenon	Heterozygote	VUS	38.43 KB	1
1552	20:49098847–49161369	q13.13	20	Duplication	Moyamoya phenomenon	Heterozygote	VUS	62.52 KB	2
1553	1:145601946–146052881	q21.1	1	Deletion	Moyamoya phenomenon	Heterozygote	VUS	450.94 KB	21
1554	1:147927436–148001589	q21.2	1	Deletion	Moyamoya phenomenon	Heterozygote	VUS	74.15 KB	2
1555	X:138578744–138615283	q26.3	X	Duplication	Moyamoya phenomenon	Heterozygote	VUS	36.54 KB	1
1556	21:8522361–46699983	p11.2‐q22.3	21	Trisomy	Moyamoya phenomenon	Unknown	Pathogenic	38.18 MB	539
1557	2:63862264–63956717	p15‐p14	2	Deletion	Moyamoya phenomenon	Heterozygote	VUS	94.45 KB	2
1558	18:52902539–53577824	q21.2	18	Duplication	Cherry red spot of the macula	Heterozygote	VUS	675.29 KB	4
1559	12:105448092–106361092	q23.3	12	Deletion	Aortic root aneurysm	Heterozygote	VUS	913.00 KB	7
1560	5:42493685–42700118	p13.1‐p12	5	Duplication	Aortic root aneurysm	Heterozygote	VUS	206.43 KB	1
1561	3:44932333–45290346	p21.31	3	Duplication	Aortic root aneurysm	Heterozygote	VUS	358.01 KB	8
1562	7:153837582–153975582	q36.2	7	Deletion	Aortic root aneurysm	Heterozygote	VUS	138.00 KB	1
1563	1:155215838–155231152	q22	1	Deletion	Aortic root aneurysm	Heterozygote	VUS	15.31 KB	2
1564	5:117080324–122405884	q23.1‐q23.2	5	Deletion	Aortic root aneurysm	Heterozygote	Likely pathogenic	5.33 MB	37
1565	4:85100449–85482699	q21.23	4	Deletion	Abdominal aortic aneurysm	Heterozygote	VUS	382.25 KB	2
1566	16:29320029–30321210	p11.2	16	Duplication	Aortic root aneurysm	Heterozygote	VUS	1.00 MB	52
1567	3:72839134–72888875	p13	3	Deletion	Aortic root aneurysm	Heterozygote	VUS	49.74 KB	4
1568	15:94225382–97781641	q26.2	15	Deletion	Left superior vena cava draining to coronary sinus	Heterozygote	VUS	3.56 MB	21
1569	1:147264477–147605870	q21.1‐q21.2	1	Duplication	Left superior vena cava draining to coronary sinus	Heterozygote	VUS	341.39 KB	6
1570	3:134222617–134401633	q22.2	3	Deletion	Left superior vena cava draining to coronary sinus	Heterozygote	VUS	179.02 KB	5
1571	19:31803110–34965684	q12‐q13.11	19	Deletion	Bilateral superior vena cava with bridging vein	Heterozygote	Pathogenic	3.16 MB	65
1572	2:189707648–202745173	q32.2‐q33.2	2	Deletion	Bilateral superior vena cava with bridging vein	Heterozygote	VUS	13.04 MB	172
1573	18:29517548–31011384	q12.1	18	Duplication	Varicose veins	Heterozygote	VUS	1.49 MB	3
1574	7:110578659–113007933	q31.1	7	Deletion	Varicose veins	Heterozygote	VUS	2.43 MB	18
1575	7:111494535–111693874	q31.1	7	Deletion	Varicose veins	Heterozygote	Likely pathogenic	199.34 KB	1
1576	21:41106203–46699983	q22.2‐q22.3	21	Deletion	Varicose veins	Heterozygote	Likely pathogenic	5.59 MB	155
1577	16:46530645–46875224	q11.2	16	Duplication	Varicose veins	Heterozygote	VUS	344.58 KB	9
1578	2:137727442–144513563	q22.1‐q22.3	2	Deletion	Arteriovenous fistulas of celiac and mesenteric vessels	Heterozygote	Pathogenic	6.79 MB	43
1579	4:85100449–85482699	q21.23	4	Deletion	Abdominal aortic aneurysm	Heterozygote	VUS	382.25 KB	2
1580	19:504595–786609	p13.3	19	Deletion	Aortic dissection	Heterozygote	VUS	282.01 KB	17
1581	16:14881435–16223192	p13.11	16	Duplication	Aortic dissection	Heterozygote	Pathogenic	1.34 MB	30
1582	11:134337919–134716294	q25	11	Deletion	Coarctation of abdominal aorta	Heterozygote	VUS	378.38 KB	4
1583	5:113461–8139878	p15.33‐p15.31	5	Deletion	Juxtaductal coarctation of the aorta	Heterozygote	Pathogenic	8.03 MB	85
1584	18:77032666–80252149	q23	18	Duplication	Preductal coarctation of the aorta	Heterozygote	VUS	3.22 MB	22
1585	4:188003957–189995523	q35.2	4	Deletion	Preductal coarctation of the aorta	Heterozygote	VUS	1.99 MB	18
1586	4:91502611–92513250	q22.1	4	Duplication	Hypoplastic aortic arch	Heterozygote	VUS	1.01 MB	5
1587	9:135337654–136026399	q34.3	9	Deletion	Hypoplastic aortic arch	Heterozygote	Likely pathogenic	688.75 KB	19
1588	1:147264477–147605870	q21.1‐q21.2	1	Duplication	Hypoplastic aortic arch	Heterozygote	VUS	341.39 KB	6
1589	2:148456951–149333909	q23.1‐q23.2	2	Deletion	Hypoplastic aortic arch	Heterozygote	Pathogenic	876.96 KB	13
1590	20:80928–19416693	p13‐p11.23	20	Deletion	Hypoplastic aortic arch	Heterozygote	Likely pathogenic	19.34 MB	261
1591	22:18162023–21444618	q11.21	22	Deletion	Vascular ring	Heterozygote	Pathogenic	3.28 MB	125
1592	6:198357–401084	p25.3	6	Deletion	Right aortic arch with mirror image branching	Heterozygote	VUS	202.73 KB	2
1593	1:1688214–1798824	p36.33	1	Deletion	Right aortic arch with mirror image branching	Heterozygote	VUS	110.61 KB	6
1594	19:8786994–8861171	p13.2	19	Duplication	Right aortic arch with mirror image branching	Heterozygote	VUS		3
1595	22:19108265–19940567	q11.21	22	Duplication	Right aortic arch with mirror image branching	Heterozygote	VUS		25
1596	10:46157934–46318429	q11.22	10	Deletion	Right aortic arch with mirror image branching	Heterozygote	VUS	160.50 KB	2
1597	19:54705633–54880532	q13.42	19	Duplication	Right aortic arch with mirror image branching	Heterozygote	VUS		12
1598	X:154094244–154323657	q28	X	Copy‐Number Gain	Right aortic arch with mirror image branching	Heterozygote	VUS	229.41 KB	10
1599	6:366332–771504	p25.3	6	Deletion	Right aortic arch with mirror image branching	Heterozygote	VUS	405.17 KB	3
1600	22:23354115–24601056	q11.23	22	Duplication	Left aortic arch with retroesophageal right subclavian artery	Heterozygote	Likely pathogenic	1.25 MB	44
1601	12:20858143–21238994	p12.2‐p12.1	12	Deletion	Aorto‐right ventricular tunnel	Heterozygote	VUS	380.85 KB	4
1602	7:88615564–90236479	q21.13	7	Duplication	Aortic tortuosity	Heterozygote	VUS	1.62 MB	
1603	22:28076186–29013583	q12.1	22	Deletion	Aortopulmonary collateral arteries	Heterozygote	VUS	937.40 KB	9
1604	15:94225382–97781641	q26.2	15	Deletion	Left superior vena cava draining to coronary sinus	Heterozygote	VUS	3.56 MB	21
1605	1:147264477–147605870	q21.1‐q21.2	1	Duplication	Left superior vena cava draining to coronary sinus	Heterozygote	VUS	341.39 KB	6
1606	3:134222617–134401633	q22.2	3	Deletion	Left superior vena cava draining to coronary sinus	Heterozygote	VUS	179.02 KB	5
1607	19:31803110–34965684	q12‐q13.11	19	Deletion	Bilateral superior vena cava with bridging vein	Heterozygote	Pathogenic	3.16 MB	65
1608	17:36346367–37829080	q12	17	Duplication	Aortopulmonary window	Heterozygote	VUS	1.48 MB	24
1609	16:14874798–16198524	p13.11	16	Duplication	Type I truncus arteriosus	Heterozygote	VUS	1.32 MB	30
1610	9:76197416–76529153	q21.13	9	Duplication	Type I truncus arteriosus	Heterozygote	VUS	331.74 KB	6
1611	13:107863172–108267138	q33.3	13	Duplication	Type I truncus arteriosus	Heterozygote	VUS	403.97 KB	4
1612	14:52230529–57883649	q22.1‐q23.1	14	Deletion	Transposition of the great arteries	Heterozygote	VUS	5.65 MB	61
1613	3:36982520–38602038	p22.2	3	Deletion	Transposition of the great arteries	Heterozygote	VUS	1.62 MB	37
1614	1:147093177–148262736	q21.1‐q21.2	1	Deletion	Transposition of the great arteries	Heterozygote	VUS	1.17 MB	27
1615	6:118371140–119216358	q22.31	6	Duplication	Transposition of the great arteries	Heterozygote	VUS	845.22 KB	10
1616	16:7693139–7724540	p13.3	16	Deletion	Transposition of the great arteries	Heterozygote	VUS	31.40 KB	1
1617	4:10415765–10578160	p16.1	4	Duplication	Transposition of the great arteries	Heterozygote	VUS	162.40 KB	2
1618	3:46811233–50787139	p21.31‐p21.2	3	Deletion	Transposition of the great arteries	Heterozygote	Pathogenic	3.98 MB	152
1619	2:3033979–3451125	p25.3	2	Duplication	Transposition of the great arteries	Heterozygote	VUS	417.15 KB	4
1620	18:3455704–3693638	p11.31	18	Duplication	Transposition of the great arteries	Heterozygote	VUS	237.94 KB	6
1621	17:14194981–15538864	p12	17	Duplication	Transposition of the great arteries	Heterozygote	VUS	1.34 MB	18
1622	22:17215100–17429717	q11.1‐q11.21	22	Duplication	Transposition of the great arteries	Heterozygote	VUS	214.62 KB	6
1623	16:14874998–16198378	p13.11	16	Duplication	Transposition of the great arteries	Heterozygote	VUS	1.32 MB	30
1624	X:122825305–123037517	q25	X	Duplication	Transposition of the great arteries	Heterozygote	VUS	212.21 KB	0
1625	3:3446847–12070901	p26.2‐p25.2	3	Deletion	Transposition of the great arteries	Heterozygote	Pathogenic	8.62 MB	95
1626	3:243725–1595242	p26.3	3	Duplication	Transposition of the great arteries	Heterozygote	Likely pathogenic	1.35 MB	9
1627	9:2602116–2818582	p24.2	9	Deletion	Transposition of the great arteries	Heterozygote	Likely pathogenic	216.47 KB	4
1628	20:3208292–3613133	p13	20	Duplication	Transposition of the great arteries	Heterozygote	Likely pathogenic	404.84 KB	7
1629	2:130720420–131158155	q21.1	2	Duplication	Transposition of the great arteries	Heterozygote	Likely pathogenic	437.74 KB	8
1630	18:78964307–80252149	q23	18	Deletion	Transposition of the great arteries	Heterozygote	VUS	1.29 MB	16
1631	2:106543025–112341109	q12.2‐q14.1	2	Duplication	Transposition of the great arteries	Heterozygote	Pathogenic	5.80 MB	90
1632	7:75467141–76611451	q11.23	7	Deletion	Transposition of the great arteries	Heterozygote	Likely pathogenic	1.14 MB	29
1633	13:65041294–70828179	q21.31‐q21.33	13	Deletion	Transposition of the great arteries	Heterozygote	Likely pathogenic	5.79 MB	35
1634	6:118416465–118733633	q22.31	6	Duplication	Transposition of the great arteries	Heterozygote	Likely pathogenic	317.17 KB	4
1635	8:1179532–2053428	p23.3	8	Duplication	Transposition of the great arteries	Heterozygote	Likely pathogenic	873.90 KB	11
1636	X:72329407–72333259	q13.1	X	Deletion	Patent ductus arteriosus after birth at term	Heterozygote	Pathogenic	3.85 KB	1
1637	7:54215–7650501	p22.3‐p21.3	7	Duplication	Patent ductus arteriosus after birth at term	Heterozygote	VUS	7.60 MB	126
1638	3:11351615–11576994	p25.3	3	Deletion	Patent ductus arteriosus after birth at term	Heterozygote	VUS	225.38 KB	2
1639	4:70295481–70415837	q13.3	4	Deletion	Patent ductus arteriosus after birth at term	Heterozygote	VUS	120.36 KB	4
1640	X:38629478–38760055	p11.4	X	Duplication	Patent ductus arteriosus after birth at term	Heterozygote	VUS	130.58 KB	1
1641	3:182535725–185194287	q26.33‐q27.2	3	Deletion	Patent ductus arteriosus after birth at term	Heterozygote	VUS	2.66 MB	64
1642	6:1599902–2887417	p25.3‐p25.2	6	Deletion	Patent ductus arteriosus after birth at term	Heterozygote	Pathogenic	1.29 MB	13
1643	22:16548165–37563699	q11.1‐q13.1	22	Duplication	Patent ductus arteriosus after birth at term	Heterozygote	Pathogenic		588
1644	16:29453527–30354824	p11.2	16	Deletion	Patent ductus arteriosus after birth at term	Heterozygote	Pathogenic	901.30 KB	51
1645	1:1164293–6646766	p36.33‐p36.31	1	Deletion	Patent ductus arteriosus after birth at term	Heterozygote	Pathogenic		127
1646	16:21940094–22419849	p12.2	16	Deletion	Patent ductus arteriosus after birth at term	Heterozygote	Pathogenic	479.76 KB	11
1647	15:99835169–101857633	q26.3	15	Deletion	Patent ductus arteriosus after birth at term	Heterozygote	Likely pathogenic	2.02 MB	32
1648	19:52145323–52473217	q13.41	19	Duplication	Patent ductus arteriosus after birth at term	Heterozygote	VUS	327.89 KB	13
1649	19:52955056–53536589	q13.41‐q13.42	19	Duplication	Patent ductus arteriosus after birth at term	Heterozygote	VUS	581.53 KB	27
1650	13:54374864–57599308	q14.3‐q21.1	13	Deletion	Patent ductus arteriosus after premature birth	Heterozygote	VUS	3.22 MB	15
1651	22:18907322–21449132	q11.21	22	Duplication	Patent ductus arteriosus after premature birth	Heterozygote	VUS		102
1652	12:129362035–131808811	q24.33	12	Deletion	Patent ductus arteriosus after premature birth	Heterozygote	Likely pathogenic		22
1653	18:58018889–64098832	q21.31‐q22.1	18	Deletion	Patent ductus arteriosus after premature birth	Heterozygote	Likely pathogenic	6.08 MB	72
1654	17:1227392–1371721	p13.3	17	Duplication	Patent ductus arteriosus after premature birth	Heterozygote	VUS		4
1655	16:14868497–16195889	p13.11	16	Duplication	Patent ductus arteriosus after premature birth	Heterozygote	VUS	1.33 MB	3
1656	1:147002202–148352220	q21.1‐q21.2	1	Duplication	Patent ductus arteriosus after premature birth	Heterozygote	Likely pathogenic	1.35 MB	36
1657	1:824379–8017899	p36.33‐p36.23	1	Deletion	Bilateral ductus arteriosus	Heterozygote	Pathogenic	7.19 MB	156
1658	X:139602445–139849294	q27.1	X	Duplication	Double outlet right ventricle with doubly committed ventricular septal defect without pulmonary stenosis	Heterozygote	VUS	246.85 KB	2
1659	X:144935166–145374689	q27.3	X	Duplication	Double outlet right ventricle with doubly committed ventricular septal defect without pulmonary stenosis	Heterozygote	VUS	439.52 KB	3
1660	22:31596389–36746187	q12.2‐q12.3	22	Deletion	Double outlet right ventricle with subaortic ventricular septal defect and pulmonary stenosis	Heterozygote	Pathogenic	5.15 MB	75
1661	7:98371236–102449202	q21.3‐q22.1	7	Deletion	Double outlet right ventricle with subaortic ventricular septal defect and pulmonary stenosis	Heterozygote	Likely pathogenic	4.08 MB	137
1662	17:11693675–11705290	p12	17	Deletion	Tetralogy of Fallot with pulmonary stenosis	Heterozygote	Pathogenic	11.62 KB	1
1663	5:129398190–134608482	q23.3‐q31.1	5	Deletion	Tetralogy of Fallot with pulmonary stenosis	Heterozygote	Likely pathogenic	5.21 MB	71
1664	2:130594260–131340224	q21.1	2	Duplication	Tetralogy of Fallot with pulmonary stenosis	Heterozygote	VUS	745.97 KB	22
1665	2:136911052–146191493	q22.1‐q22.3	2	Deletion	Tetralogy of Fallot with pulmonary stenosis	Heterozygote	VUS	9.28 MB	54
1666	17:20492297–20698718	p11.2	17	Deletion	Tetralogy of Fallot with absent pulmonary valve	Heterozygote	VUS	206.42 KB	10
1667	8:16240338–30579491	p22‐p12	8	Deletion	Tetralogy of Fallot with absent pulmonary valve	Heterozygote	VUS	14.34 MB	179
1668	X:6570680–8129470	p22.31	X	Duplication	Congenitally corrected transposition of the great arteries with ventricular septal defect	Heterozygote	VUS	1.56 MB	7
1669	8:8246125–11995479	p23.1	8	Deletion	Peripheral pulmonary artery stenosis	Heterozygote	Pathogenic	3.75 MB	58
1670	7:73976261–74031476	q11.23	7	Duplication	Peripheral pulmonary artery stenosis	Heterozygote	Likely pathogenic	55.22 KB	1
1671	7:73197914–74345185	q11.23	7	Deletion	Peripheral pulmonary artery stenosis	Heterozygote	VUS	1.15 MB	35
1672	2:213413709–215325273	q34‐q35	2	Deletion	Peripheral pulmonary artery stenosis	Heterozygote	VUS	1.91 MB	
1673	7:72382983–73976980	q11.22‐q11.23	7	Deletion	Peripheral pulmonary artery stenosis	Heterozygote	Likely pathogenic	1.59 MB	47
1674	8:29610911–30490983	p12	8	Duplication	Peripheral pulmonary artery stenosis	Heterozygote	VUS	880.07 KB	18
1675	1:26651863–26930104	p36.11	1	Duplication	Pulmonary artery dilatation	Heterozygote	VUS	278.24 KB	9
1676	1:26651863–26930104	p36.11	1	Duplication	Pulmonary artery atresia	Heterozygote	VUS	278.24 KB	9
1677	3:37050639–37780036	p22.2	3	Duplication	Pulmonary artery hypoplasia	Heterozygote	VUS	729.40 KB	14
1678	20:17871356–25871364	p12.1‐p11.1	20	Duplication	Pulmonary artery hypoplasia	Heterozygote	Likely pathogenic	8.00 MB	135
1679	22:17446408–25579479	q11.21‐q12.1	22	Deletion	Pulmonary artery hypoplasia	Heterozygote	Likely pathogenic	8.13 MB	334
1680	10:116650499–133334830	q25.3‐q26.3	10	Deletion	Pulmonary artery hypoplasia	Heterozygote	Likely pathogenic	16.68 MB	180
1681	20:5071354–21371362	p13‐p11.22	20	Deletion	Pulmonary artery hypoplasia	Heterozygote	Likely pathogenic	16.30 MB	167
1682	7:10239–7087129	p22.3‐p22.1	7	Duplication	Pulmonary artery hypoplasia	Heterozygote	Likely pathogenic	7.08 MB	120
1683	7:148165260–159253352	q35‐q36.3	7	Deletion	Pulmonary artery hypoplasia	Heterozygote	Likely pathogenic	11.09 MB	147
1684	16:29584162–29827124	p11.2	16	Duplication	Hypoplastic pulmonary veins	Heterozygote	VUS	242.96 KB	15
1685	16:30085268–30181240	p11.2	16	Duplication	Hypoplastic pulmonary veins	Heterozygote	VUS	95.97 KB	6
1686	4:35672405–35716298	p15.1	4	Deletion	Hypoplastic pulmonary veins	Heterozygote	VUS	43.89 KB	0
1687	14:51787673–51790976	q22.1	14	Deletion	Hypoplastic pulmonary veins	Heterozygote	VUS	3.30 KB	0
1688	5:56016213–56038049	q11.2	5	Deletion	Hypoplastic pulmonary veins	Heterozygote	VUS	21.84 KB	0
1689	20:59436575–59446328	q13.32	20	Deletion	Hypoplastic pulmonary veins	Heterozygote	VUS	9.75 KB	0
1690	15:28917417–30076384	q13.1‐q13.2	15	Deletion	Scimitar anomaly	Heterozygote	Likely pathogenic	1.16 MB	8
1691	15:22655582–23107440	q11.2	15	Deletion	Supracardiac total anomalous pulmonary venous connection	Heterozygote	Likely pathogenic	451.86 KB	7
1692	6:60001–34106686	p25.3‐p21.31	6	Uniparental Isodisomy	Cerebral arteriovenous malformation	Heterozygote	VUS	34.05 MB	738
1693	22:18949869–21111371	q11.21	22	Duplication	Cerebral arteriovenous malformation	Heterozygote	Likely pathogenic	2.16 MB	84
1694	13:94800976–99909036	q32.1‐q32.3	13	Deletion	Cerebral arteriovenous malformation	Heterozygote	Likely pathogenic	5.11 MB	70
1695	5:9846888–15046891	p15.31‐p15.1	5	Deletion	Cerebral arteriovenous malformation	Heterozygote	Likely pathogenic	5.20 MB	38
1696	7:10239–7087129	p22.3‐p22.1	7	Duplication	Cerebral arteriovenous malformation	Heterozygote	Likely pathogenic	7.08 MB	120
1697	2:137727442–144513563	q22.1‐q22.3	2	Deletion	Arteriovenous fistulas of celiac and mesenteric vessels	Heterozygote	Pathogenic	6.79 MB	43
1698	16:84789514–85838411	q24.1	16	Duplication	Intestinal lymphangiectasia	Heterozygote	VUS	1.05 MB	17
1699	18:4405472–4979613	p11.31	18	Duplication	Intestinal lymphangiectasia	Heterozygote	VUS	574.14 KB	2
1700	20:7125642–8595024	p12.3	20	Deletion	Intestinal lymphangiectasia	Heterozygote	VUS	1.47 MB	11
1701	1:170083396–170370370	q24.2	1	Deletion	Intestinal lymphangiectasia	Heterozygote	VUS	286.98 KB	9
1702	18:52902539–53577824	q21.2	18	Duplication	Cherry red spot of the macula	Heterozygote	VUS	675.29 KB	4
1703	21:32885837–33915909	q22.11	21	Deletion	Internal carotid artery hypoplasia	Heterozygote	Pathogenic	1.03 MB	24

Abbreviation: VUS, variant of uncertain significance.

### 4.1 Familial hypercholesterolaemia

FH is an inherited dyslipidaemia with autosomal‐dominant inheritance, characterized by disease‐causing variants in low‐density lipoprotein cholesterol (*LDLR*) (60%–80%), apolipoprotein B (*APOB*) (5%–10%) or proprotein convertase subtilisin/kexin type 9 (*PCSK9*) (<1%).^46^, ^47^ Predominantly, small‐scale missense, splicing and frameshift variants constitute the changes in FH, yet CNVs also account for about 10% of cases.^48^ A study by Goldstein and Brown used southern blotting to identify DNA variants, and they found that CNVs and structural rearrangements of the LDLR are common types of mutations in patients with positive FH.^49^ In another study, various CNVs present in *LDLR*, including a 15‐kb deletion spanning the promoter and exon 1 in 63% of French Canadians with FH,^50^ as well as a 9.5‐kb deletion spanning exons 16–18 in 30%–40%, were identified as founder effect mutations, significantly contributing to the high proportion of FH in specific populations.^51^, ^52^ The *LDLR* locus harbours a high frequency of the most common repetitive element in the genome, the Arthrobacter luteus (Alu) sequences, facilitating CNV mutagenesis through mechanisms associated with defective DNA repair, replication and recombination, such as NHEJ, FoSTeS and/or non‐allelic homologous recombination.^53^, ^54^, ^55^ Consequently, the *LDLR* locus is predisposed to acquiring CNVs over time to acquire. In recent years, molecular diagnosis of FH utilizing next‐generation sequencing (NGS) panels has emerged as a method to identify CNVs, offering an alternative approach to traditional approaches.^56^ A study by Zhou Y. et al. revealed novel CNVs in the *APOB* gene in Europeans (0.09%).^57^ In another study, targeted NGS data uncovered a whole‐gene duplication of *PCSK9* in Canadian FH patients, making the first report of a CNV affecting the *PCSK9* gene in FH.^58^ A study by Iacocca M. et al. detected 38 whole‐exon CNVs in the *LDLR* gene and 2 large‐scale duplications in the *PCSK9* gene using an NGS approach.^56^

### 4.2 Hereditary cardiomyopathies

Cardiomyopathies represent a heterogeneous group of diseases associated with cardiac muscle or electrical dysfunction.^59^ The main types of cardiomyopathy include dilated cardiomyopathy (DCM), hypertrophic cardiomyopathy (HCM), restrictive cardiomyopathy (RCM) and arrhythmogenic cardiomyopathy (ACM).^60^ CNVs have been observed predominantly in patients with DCM, HCM, ACM and left ventricular non‐compaction cardiomyopathy (LVNC), while only a few studies have shown CNVs in RCM.^61^ In a study on CNVs, a significant role of rare variants in a large cohort causing DCM was demonstrated,^62^ these CNVs were deletion in exon 4 of *HSP* co‐chaperone *BCL2*‐associated athanogene 3 (*BAG3*), detected through whole‐exome sequencing (WES).^62^ In another study, 20 additional or partial candidate genes were analysed, and a dedicated bioinformatics algorithm was employed to detect CNVs. Consequently, 1.3% of patients exhibited pathogenic CNVs, including two deletions in *MYBPC3* and two deletions involving the entire *PLN* coding region.^63^ As a genetic method for clinical diagnosis on a predefined panel of HCM‐related genes, NGS technology facilitates the detection of large genomic region CNVs.^64^, ^65^ Other CNVs associated with heart diseases include two deletions in *MYBPC3* (HCM) and *TTN* (DCM) genes, classified as pathogenic, a repeat of *MYH7* (HCM) as a VUS and a deletion of *CTNNA3* (DCM), which were classified as intragenic deletion.^66^ In a study by Fedida et al., WES‐based CNV analysis in cardiomyopathy patients revealed clinically significant CNVs in arrhythmogenic right ventricular cardiomyopathy/dysplasia (ARVC/D) patients without disease‐causing mutations in known desmosomal genes.^67^ WES detected large deletions of *PKP2* that were not detected by the standard Sanger sequencing.^67^ Another study reported the frequency of CNVs among cardiomyopathy cases as 2.3% (20 out of 874),^68^ with detection rates of 1.2% for HCM, 4.4% for DCM, 5.1% for ACM and 3.4% for LVNC.^68^ Also, the Ceyhan‐Birsoy et al.'s study reported CNV frequencies of 0.56% for HCM, 0.6% for DCM, 1% for ACM and 1.9% for LVNC.^65^ Another study highlighted a CNV frequency of 0.8% in HCM patients during a comprehensive CNV screening.^64^

### 4.3 Sudden cardiac death and channelopathies

Several studies have reported the role of CNVs in heart diseases related to SCD and channelopathies.^62^, ^65^, ^69^, ^70^, ^71^, ^72^, ^73^, ^74^ SCD is characterized as a natural, unexpected, non‐traumatic death, arising from a known or unknown cardiovascular cause.^75^ Channelopathies constitute a group of disorders characterized by abnormalities in ion channel and transporter function, caused by either genetic or acquired factors.^76^ The most common cause of channelopathies is mutations in the genes encoding ion channels, leading to disrupted channel function.^76^ In one study, a deletion in the *KCNH2* gene (SUD) was identified as pathogenic.^66^ Another study revealed that the frequency of CNVs among sudden unexplained death (SUD) cases was 1.4% (8 out of 587), and 2.6% (8 out of 304) in patients with channelopathies.^68^ The detection rate for long QT syndrome (LQTS) was notably high at 4.7%.^68^ CNVs, including duplications in the *TRDN* and *CASQ2* genes, were linked to Brugada syndrome (BrS).^68^ Additionally, five duplications and one deletion CNV in the *KCNQ1*, *KCNE1*, *KCNH2* and *NEXN* genes were associated with LQTS.^68^ CNVs related to SUD included two deletions and six duplications and were identified in the *PDLIM3*, *PKP2*, *CASQ2*, *EMD*, *TNNI3*, *TAZ*, *KCNE1* and *RANGRF* genes.^68^ The prevalence of each disease in connection with channelopathies was as follows: 1.3% for BrS (2 out of 151 patients), 0% for catecholaminergic polymorphic ventricular tachycardia (CPVT) (0 out of 19 patients), 4.7% for LQTS (6 out of 127 patients) and 0% for SQTS (0 out of 7 patients).^68^ In a distinct study, CNV screening in SUD/sudden infant death (SID) cases was documented.^69^ A de novo CNV associated with SID was detected in 11% of patients,^69^ including a 3‐Mb duplication on chromosome 8 and a 4.4‐Mb deletion on chromosome 22 in one patient. Additionally, a 240‐kb duplication and a 1.9‐Mb deletion on chromosome 6 were discovered in two separate cases.^69^

### 4.4 Congenital heart disease

CHD is the most common birth defect in humans.^77^ Recently, CNVs have been recognized as an important contributor to CHD.^78^ Identification of CNVs in CHD has elucidated the mechanisms influencing heart morphogenesis.^78^ CNVs impact dose‐sensitive transcriptional regulators required vital for cardiac development, exhibiting frequent enrichment at multiple genomic hotspots (1q21.1, 2q13, 3p25.1, 7q11.13, 8p23.1, 11q24, 15q11.2, 16p11.2 and 22q11.2) in CHD cohorts.^79^, ^80^ Candidate genes within these CNVs include *ELN* (associated with Williams syndrome), *RAI1* (Smith–Magenis syndrome), *GATA4* (8p23.1 deletion), *GJA5* (1q21.1 duplication), *TBX1* and *CRKL* (22q11 deletion), and *TBX6*, *ETS1* (11q24.2‐q25 deletions in Jacobsen syndrome), along with *FBLN7*, *TMEM87B*, *MAPK1* and *CYFIP1*, which play roles in the early embryonic heart and aortic arch development.^79^, ^80^, ^81^ The most recurrent CNV linked with syndromic CHD is a 3‐Mb canonical deletion of 22q11.2.^80^ In a study by Glessner JT et al., recurrent de novo CNVs at 15q11.2, encompassing *CYFIP1*, *NIPA1* and *NIPA2* genes and single de novo CNVs containing *JUP*, *JUN*, *MED9*, *MED15*, *DUSP1*, *ZEB2*, *TOP2A*, *SREBF1* and *PTPRE* genes were identified. These genes interact with *NKX2*‐5 and *GATA4*, which are key proteins in CHD.^81^ Saacks NA et al. demonstrated CNVs encompassing five genes—*DGCR8*, *KDM2A*, *JARID2*, *FSTL1* and *CYFIP1*—likely contributing to CHD development.^82^ CNVs are estimated to be involved in approximately 3%–25% of CHD cases with extracardiac abnormalities (ECAs) and 3%–10% of cases with isolated CHD.^27^ Genetic testing for CHD, focusing on CNVs, is crucial for gene detection and diagnosis. Furthermore, in a cohort study of 46 non‐syndromic individuals, rare CNVs were identified, encompassing a variety of CHD types, as well as in syndromic and non‐syndromic congenital heart defects.^83^, ^84^

### 4.5 Thoracic aortic aneurysms and dissections

Thoracic aortic aneurysm and dissection (TAAD) often remain undiagnosed until they result in aortic rupture or dissection. Alarmingly, approximately one‐fifth of patients experiencing acute aortic dissection perish before reaching a medical facility.^85^ Researchers have proposed three primary pathological mechanisms in genes associated with TAAD: abnormal extracellular matrix components, altered transforming growth factor‐ß signalling and disruption in smooth muscle cell function.^85^ A large 10 mb CNV, deleting 11 genes, including *ACTA2* (actin alpha 2), was identified in a TAAD patient.^86^ In a study by Yang et al., targeted NGS identified at least likely pathogenic variants in 84.5% of syndromic and 18.7% of non‐syndromic TAAD patients.^85^ A large cohort including a broad range of participants, reported CNVs in approximately 8% of TAAD patients.^87^ Notably, the *FBN1* variant has been identified as the most causative variant in TAAD patients.^85^, ^87^, ^88^ CNVs are more prevalent in patients with early‐onset TAAD (32%) compared to those with familial (23%), and sporadic (17%) TAAD.^89^ The presence of (likely) pathogenic variants is significantly associated with early onset, a positive family history of TAAD and syndromic features.^85^, ^87^, ^88^

### 4.6 Hypertension

Hypertension, characterized by elevated blood pressure, is a major risk factor for cardiovascular and renal diseases. Hypertension is predominantly idiopathic and classified as essential hypertension.^90^ While it is recognized to have a hereditary component, the genetic predisposition remains elusive. The contribution of CNVs to this genetic predisposition is yet to be determined.^91^, ^92^ In an animal study involving Wistar‐Kyoto rats, a notable association was observed between elevated blood pressure and an increased copy number in the *Egln1* gene.^90^ Marques et al. demonstrated that patients with extremely high blood pressure more frequently exhibited CNVs on chromosome 1. While CNVs appeared more frequently in hypertensive patients, the association did not reach statistical significance.^91^ A study focusing on women identified four CNVs (*PAX8*, *PPH2*, *HYT3* and *BMPR2*) on chromosome 2 linked to hypertension, underscoring potential gender‐specific differences in CNV maps. Notably, disparities in chromosomes 18 and X at particular loci could signify CNVs related to hypertension.^92^ CNVs are also posited to influence the development of left ventricular hypertrophy in hypertensive patients by affecting the regulation of the fetal cardiac gene programme.^93^ Furthermore, in the study conducted by Shia WC et al., seven CNV regions were significantly associated with hyperlipidaemia and myocardial infarction in patients, as indicated by multivariate analysis (*p* < 0.001).^94^

## 5 TECHNIQUES TO DETECT CNVS

Fluorescence in situ hybridization (FISH) is a microscopic method employed for detecting CNVs.^95^ The FISH technique determines the presence or absence of specific target DNA sequences through the hybridization of sequence‐specific fluorescently labelled probes, followed by the microscopic detection of a fluorescent signal.^96^ Structural variants are analysed using both microscopic (visible with a microscope) and submicroscopic (not detectable solely by microscope) methods.^15^ To detect CNVs, various submicroscopic laboratory‐based approaches have been developed, including PCR‐based arrays (such as real‐time PCR),^95^ multiplex ligation‐dependent probe amplification (MLPA),^97^ and microarray‐based methods (such as comparative genomic hybridization (aCGH) and SNP microarrays),^98^ alongside NGS techniques (including clone‐based sequencing and exome sequencing) and RNA sequencing (RNA‐Seq).^99^

FISH and aCGH are recognized as molecular cytogenetic methods.^96^ aCGH, in particular, serves as an effective tool to measure structural changes in the genome, offering significantly higher resolution than conventional karyotyping.^100^ However, the resolution of CGH alone does not suffice for accurate mapping of variant breakpoints or identifying copy neutral or balanced rearrangements. Therefore, NGS techniques have been advanced to detect CNVs, enhancing the investigation of these variants.^101^

The MLPA assay is adept at detecting CNVs, encompassing large and small deletions and single‐nucleotide aberrations across multiple human genes simultaneously.^102^ Its high accuracy, efficiency and cost‐effectiveness make MLPA a variable alternative to array‐based techniques.^103^ In MLPA, separation is fundamentally based on the size of the amplification products, facilitating the differentiation of sequences up to approximately 50 base pairs.^104^ Recently, the RNA‐Seq method for CNV detection has been developed,^105^, ^106^ introducing innovative approaches for analysis. Notable methods include visual control of expression profiles from scRNA‐Seq data (inferCNV2), detection of CNVs from scRNA‐Seq data (HoneyBADGER21) and statistical integration of RNA‐Seq and DNA‐Seq independent of single cells from human cancers (clonealign).^99^

In recent years, NGS techniques have offered high resolution, accurate and rapid identification and analysis of CNVs through whole‐genome sequencing (WGS) and WES.^107^ These methods enable the precise identification of breakpoint positions within CNVs.^108^ WES, in particular, is recognized for its exceptional ability to identify rare and private single‐nucleotide variations, as well as insertions and deletions, which underlie the onset of disease.^109^

NGS presents advantages over microarray methods, including superior high quality, speed and cost‐efficiency.^110^ Two main strategies for CNV detection using NGS data are paired‐end mapping (PEM) and depth of coverage (DOC).^111^ PEM‐based methods detect insertions and deletions by comparing the distance between alignment sites of read pairs against the average insert size of a genomic library.^112^ While PEM‐based methods are highly sensitive for detecting deletions smaller than 1 kb, they do not capture all types of SVs in complex genomic regions rich in segmental duplications.^113^ Therefore, DOC‐based methods, which count the number of mapped reads in fixed length, non‐overlapping windows and then correct each window by GC content, are also employed.^112^ WES data, in contrast to WGS, allows for more accurate detection of CNVs using a DOC‐based calling approach by providing greater coverage of the coding regions of the genome.^114^

Data quality issues remain despite developments in CNV detection, and integrating family information in detecting and interpreting CNVs has been suggested to enhance CNV calling. It has been speculated that employing family segregation could reduce Mendelian inconsistencies and false‐positive genotype calls.^115^

## 6 DISCUSSION

We reviewed the potential role of CNVs in CVDs by collecting and analysing all reported CNVs associated with CVDs. Considering that CNVs in the coding region directly affect gene copy numbers at the transcriptional level, they can alter the phenotype, as well as the development and progression of CVDs.^15^ On the other hand, the size and ubiquity of CNVs are considered key factors in susceptibility to complex and polygenic CVDs.^14^ The frequency of the majority of CNVs associated with monogenic disorders overlapping with heart disease ranges from 1% to 5%.^14^ Although numerous CVDs are associated with SNPs, CNVs also play an important role in the development of various heart diseases.^15^ Until now, identified CNVs have been predominantly reported in a few cardiovascular conditions (e.g. cardiac defects, valvulopathies, cardiomegaly, TGA, hypertension and hypoplastic left heart), with nearly 10% of CNVs reported in patients with valvopathy. Therefore, CNV evaluation could be considered in patients with cardiac valvulopathies. Details on the CNV type, zygosity, pathogenicity and chromosomal location are provided in Table 1.

Among the different types of CNVs, deletions are the most common, accounting for 53.4% of cases, followed by duplications at 32.6%. Other types include trisomy (0.83%), triplication (0.99%), monosomy (0.2%), duplication/triplication (0.2%), uniparental isodisomy (0.2%) and copy number gain (0.26%). The heterozygote form is predominant at 97.5%, compared to the homozygote (1.53%) and hemizygote (0.05%) forms. Copy number gains can arise from duplications, triplications or several instances of copy number gains. The majority of deletions occur due to the loss of copy numbers at a single locus, known as heterozygous deletions. However, homozygous deletions affecting both loci do occur.^116^

Deletion‐type CNVs lead to haploinsufficiency with known consequences for some genes, while interpreting the pathogenicity of duplications is far more challenging. A higher threshold is considered for duplications compared to deletion CNVs.^117^ Nonetheless, the effect of the size of the CNV is more prominent and well‐known. Larger CNVs encompass more genes and are thus more likely to affect a dose‐sensitive one.^117^, ^118^

Our study indicates that VUS in CVDs has a higher rate of pathogenicity (43.6%) compared to likely pathogenic (28.6%) and pathogenic (16.5%) classifications. In other studies, the pathogenic role of VUS in CVDs such as HCM has been corroborated.^119^, ^120^, ^121^ Today, different methods, including clustered regularly interspaced short palindromic repeats (CRISPR) and human‐induced pluripotent stem cells (iPSCs), are employed to determine the pathogenicity of VUS in CVDs.^122^, ^123^ Therefore, our findings suggest that VUS probably has a major pathogenic role in CVDs. While a recent cohort study demonstrated that some VUS SNP variants could be reclassified upon reevaluation,^124^ a similar cohort study focusing on CNVs in CVD is yet to be conducted to determine the reclassification rate and prognostic value of VUS CNV variants in CVD. We suggest clinicians periodically reassess the CNVs, especially VUS, given they might reclassify.

According to our data, pulmonic stenosis (PS) has the highest prevalence (13.98%) among heart diseases, occurring in 7%–12% of all CHDs.^125^, ^126^ CNVs are associated with earlier onset, familial history and syndromic features, indicating their potential diagnostic value in early diagnosis of TAAD in patients with a positive family history or syndromic features to prevent acute aortic dissection. CNVs may not only be associated with hypertension but also with left ventricular hypertrophy in hypertensive patients. We recommend considering aCGH to identify CNVs in selected CVD patients, such as those with a positive familial history and syndromic features. aCGH is especially suited when the likelihood of identifying a CNV is high. Conversely, WGS should be considered when a comprehensive investigation of CNVs, alongside other types of mutation, is mandatory.

In conclusion, CNVs are now an important topic in the realm of genomic variation, biological consequences and disease development. Therefore, considering the importance of CNVs, further research is essential to fully understand the concepts and potential applications of human genomic CNVs in disease detection.

## AUTHOR CONTRIBUTIONS

**Niloofar Naderi:** Investigation (equal); methodology (equal); resources (equal); software (equal); writing – original draft (equal). **MohammadHossein MozafaryBazargany:** Investigation (equal); methodology (equal); resources (equal); software (equal); writing – original draft (equal). **Majid Maleki:** Conceptualization (equal); data curation (equal); formal analysis (equal); validation (equal); visualization (equal); writing – review and editing (equal). **Samira Kalayinia:** Conceptualization (lead); data curation (lead); investigation (lead); project administration (lead); supervision (lead); validation (lead); visualization (lead); writing – review and editing (lead).

## FUNDING INFORMATION

The authors received no specific funding for this research.

## CONFLICT OF INTEREST STATEMENT

The authors have no conflict of interest to declare.

## DECLARATION OF GENERATIVE AI AND AI‐ASSISTED TECHNOLOGIES IN THE WRITING PROCESS

During the preparation of this work, the authors used ChatGPT 4 in order to ensure English language fluency and native‐quality writing. ChatGPT 4 was consulted regarding grammar, word choice, sentence structure and overall clarity of expression. After using this service, the authors reviewed and edited the content as needed and take full responsibility for the content of the publication.

## Data Availability

All data generated or analysed during this study are included in this published article [and its supplementary information files].

## References

[1] Thiriet M . Cardiovascular disease: an introduction. Vasculopathies. 2018;8:1‐90.

[2] Amini M , Zayeri F , Salehi M . Trend analysis of cardiovascular disease mortality, incidence, and mortality‐to‐incidence ratio: results from global burden of disease study 2017. BMC Public Health. 2021;21(1):1‐12. 33632204 10.1186/s12889-021-10429-0 PMC7905904

[3] Kim HC . Epidemiology of cardiovascular disease and its risk factors in Korea. Global Health Med. 2021;3:134‐141. 10.35772/ghm.2021.01008 PMC8239378 34250288

[4] Ruan Y , Guo Y , Zheng Y , et al. Cardiovascular disease (CVD) and associated risk factors among older adults in six low‐and middle‐income countries: results from SAGE wave 1. BMC Public Health. 2018;18(1):1‐13. 10.1186/s12889-018-5653-9 PMC6011508 29925336

[5] Benjamin EJ , Muntner P , Alonso A , et al. Heart disease and stroke statistics—2019 update: a report from the American Heart Association. Circulation. 2019;139(10):e56‐e528. 30700139 10.1161/CIR.0000000000000659

[6] Regassa LD , Tola A , Ayele Y . Prevalence of cardiovascular disease and associated factors among type 2 diabetes patients in selected hospitals of Harari region, eastern Ethiopia. Front Public Health. 2021;8:532719. 33614562 10.3389/fpubh.2020.532719 PMC7892600

[7] Sonawane AR , Aikawa E , Aikawa M . Connections for matters of the heart: network medicine in cardiovascular diseases. Front Cardiovascu Med. 2022;9:1174. 10.3389/fcvm.2022.873582 PMC9160390 35665246

[8] Masironi R . Geochemistry and cardiovascular diseases. Philos Trans Royal Soc London B, Biol Sci. 1979;288(1026):193‐203. 10.1098/rstb.1979.0101 43533

[9] Bemanalizadeh M , Farajzadegan Z , Golshiri P . Estimation of cardiovascular disease risk factors in the undefined participants of campaign in Isfahan in 2017. Int J Prev Med. 2021;12:47. 34211678 10.4103/ijpvm.IJPVM_361_19 PMC8223910

[10] Hajar R . Risk factors for coronary artery disease: historical perspectives. Heart Views. 2017;18(3):109‐114. 29184622 10.4103/HEARTVIEWS.HEARTVIEWS_106_17 PMC5686931

[11] Vrablik M , Dlouha D , Todorovova V , Stefler D , Hubacek JA . Genetics of cardiovascular disease: how far are we from personalized CVD risk prediction and management? Int J Mol Sci. 2021;22(8):4182. 33920733 10.3390/ijms22084182 PMC8074003

[12] Kathiresan S , Srivastava D . Genetics of human cardiovascular disease. Cell. 2012;148(6):1242‐1257. 22424232 10.1016/j.cell.2012.03.001 PMC3319439

[13] Shukla H , Mason JL , Sabyah A . Identifying genetic markers associated with susceptibility to cardiovascular diseases. Advances in Medical Biochemistry, Genomics, Physiology, and Pathology. Jenny Stanford Publishing; 2021:177‐200.

[14] Pollex RL , Hegele RA . Copy number variation in the human genome and its implications for cardiovascular disease. Circulation. 2007;115(24):3130‐3138. 17576883 10.1161/CIRCULATIONAHA.106.677591

[15] Vijay A , Garg I , Ashraf MZ . Perspective: DNA copy number variations in cardiovascular diseases. Epigenet Insights. 2018;11:2516865718818839. 30560231 10.1177/2516865718818839 PMC6291864

[16] De Oliveira R , Rimbert H , Balfourier F , et al. Structural variations affecting genes and transposable elements of chromosome 3B in wheats. Front Genet. 2020;11:891. 33014014 10.3389/fgene.2020.00891 PMC7461782

[17] Dvirnas A , Stewart C , Müller V , et al. Detection of structural variations in densely‐labelled optical DNA barcodes: a hidden Markov model approach. PLoS One. 2021;16(11):e0259670. 34739528 10.1371/journal.pone.0259670 PMC8570516

[18] Mahmoud M , Gobet N , Cruz‐Dávalos DI , Mounier N , Dessimoz C , Sedlazeck FJ . Structural variant calling: the long and the short of it. Genome Biol. 2019;20(1):1‐14. 31747936 10.1186/s13059-019-1828-7 PMC6868818

[19] Li X , Wang L , Tan X , Wang W . Genome plasticity and endocrine diseases. Translational and Applied Genomics. 2020:211‐235.

[20] Canales CP , Walz K . The mouse, a model organism for biomedical research. Cellular and animal models in human genomics research. Elsevier; 2019:119‐140.

[21] Ellenbroek B , Youn JU . Gene‐environment interactions in psychiatry: nature, nurture, neuroscience. Academic Press; 2016.

[22] Zhang K , Lin G , Han D , Han Y , Peng R , Li J . Adaptation of ACMG‐ClinGen technical standards for copy number variant interpretation concordance. Front Genet. 2022;13:201. 10.3389/fgene.2022.829728 PMC8960312 35360839

[23] Minoche AE , Lundie B , Peters GB , et al. ClinSV: clinical grade structural and copy number variant detection from whole genome sequencing data. Genome Med. 2021;13(1):1‐19. 33632298 10.1186/s13073-021-00841-x PMC7908648

[24] Godoy VCSM , Bellucco FT , Colovati M , Oliveira‐Junior HR , Moysés‐Oliveira M , Melaragno MI . Copy number variation (CNV) identification, interpretation, and database from Brazilian patients. Genet Mol Biol. 2020;43:e20190218. 33306777 10.1590/1678-4685-GMB-2019-0218 PMC7783508

[25] Richards S , Aziz N , Bale S , et al. Standards and guidelines for the interpretation of sequence variants: a joint consensus recommendation of the American College of Medical Genetics and Genomics and the Association for Molecular Pathology. Genet Med. 2015;17(5):405‐423. 25741868 10.1038/gim.2015.30 PMC4544753

[26] Riggs ER , Andersen EF , Cherry AM , et al. Technical standards for the interpretation and reporting of constitutional copy‐number variants: a Joint Consensus Recommendation of the American College of Medical Genetics and Genomics (ACMG) and the Clinical Genome Resource (ClinGen). Elsevier; 2020. 10.1038/s41436-019-0686-8 PMC7313390 31690835

[27] Lander J , Ware SM . Copy number variation in congenital heart defects. Curr Genet Med Rep. 2014;2(3):168‐178.

[28] De Smith A , Walters R , Froguel P , Blakemore A . Human genes involved in copy number variation: mechanisms of origin, functional effects and implications for disease. Cytogenet Genome Res. 2008;123(1–4):17‐26. 19287135 10.1159/000184688 PMC2920180

[29] Muñoz‐Amatriaín M , Eichten SR , Wicker T , et al. Distribution, functional impact, and origin mechanisms of copy number variation in the barley genome. Genome Biol. 2013;14(6):1‐17. 10.1186/gb-2013-14-6-r58 PMC3706897 23758725

[30] Bickhart DM , Liu GE . The challenges and importance of structural variation detection in livestock. Front Genet. 2014;5:37. 24600474 10.3389/fgene.2014.00037 PMC3927395

[31] Pös O , Radvanszky J , Buglyó G , et al. DNA copy number variation: Main characteristics, evolutionary significance, and pathological aspects. Biom J. 2021;44(5):548‐559. 10.1016/j.bj.2021.02.003 PMC8640565 34649833

[32] Arlt MF , Wilson TE , Glover TW . Replication stress and mechanisms of CNV formation. Curr Opin Genet Dev. 2012;22(3):204‐210. 22365495 10.1016/j.gde.2012.01.009 PMC3371136

[33] Truong LN , Li Y , Shi LZ , et al. Microhomology‐mediated end joining and homologous recombination share the initial end resection step to repair DNA double‐strand breaks in mammalian cells. Proc Natl Acad Sci. 2013;110(19):7720‐7725. 23610439 10.1073/pnas.1213431110 PMC3651503

[34] Hastings PJ , Lupski JR , Rosenberg SM , Ira G . Mechanisms of change in gene copy number. Nat Rev Genet. 2009;10(8):551‐564. 19597530 10.1038/nrg2593 PMC2864001

[35] Sun Z , Liu P , Jia X , et al. Replicative mechanisms of CNV formation preferentially occur as intrachromosomal events: evidence from Potocki–Lupski duplication syndrome. Hum Mol Genet. 2013;22(4):749‐756. 23161748 10.1093/hmg/dds482 PMC3554201

[36] Segar MW , Sakofsky CJ , Malkova A , Liu Y . MMBIRFinder: a tool to detect microhomology‐mediated break‐induced replication. IEEE/ACM Trans Comput Biol Bioinform. 2014;12(4):799‐806. 10.1109/TCBB.2014.2359450 PMC4857593 26357319

[37] Bourque G , Burns KH , Gehring M , et al. Ten things you should know about transposable elements. Genome Biol. 2018;19(1):1‐12. 30454069 10.1186/s13059-018-1577-z PMC6240941

[38] Ray DA , Batzer MA . Reading TE leaves: new approaches to the identification of transposable element insertions. Genome Res. 2011;21(6):813‐820. 21632748 10.1101/gr.110528.110 PMC3106314

[39] Wells JN , Feschotte C . A field guide to eukaryotic transposable elements. Annu Rev Genet. 2020;54:539‐561. 32955944 10.1146/annurev-genet-040620-022145 PMC8293684

[40] Goodier JL , Kazazian HH . Retrotransposons revisited: the restraint and rehabilitation of parasites. Cell. 2008;135(1):23‐35. 18854152 10.1016/j.cell.2008.09.022

[41] Cordaux R , Batzer MA . The impact of retrotransposons on human genome evolution. Nat Rev Genet. 2009;10(10):691‐703. 19763152 10.1038/nrg2640 PMC2884099

[42] Babushok DV , Kazazian HH Jr . Progress in understanding the biology of the human mutagen LINE‐1. Hum Mutat. 2007;28(6):527‐539. 17309057 10.1002/humu.20486

[43] Mates J , Mademont‐Soler I , Fernandez‐Falgueras A , et al. Sudden cardiac death and copy number variants: what do we know after 10 years of genetic analysis? Forensic Sci Int: Genet. 2020;47:102281. 32248082 10.1016/j.fsigen.2020.102281

[44] Glessner JT , Li J , Desai A , et al. CNV Association of Diverse Clinical Phenotypes from eMERGE reveals novel disease biology underlying cardiovascular disease. Int J Cardiol. 2020;298:107‐113. 31447229 10.1016/j.ijcard.2019.07.058

[45] Masson E , Zou W‐B , Génin E , et al. Expanding ACMG variant classification guidelines into a general framework. Hum Genomics. 2022;16(1):31. 35974416 10.1186/s40246-022-00407-x PMC9380380

[46] Iacocca MA , Wang J , Dron JS , et al. Use of next‐generation sequencing to detect LDLR gene copy number variation in familial hypercholesterolemia. J Lipid Res. 2017;58(11):2202‐2209. 28874442 10.1194/jlr.D079301 PMC5665663

[47] Santos RD . Advancing prediction of pathogenicity of familial hypercholesterolemia LDL receptor commonest variants with machine learning models. American College of Cardiology Foundation; 2021:828‐830. 10.1016/j.jacbts.2021.10.008 PMC8617596 34869945

[48] Rutkowska L , Pinkier I , Sałacińska K , et al. Identification of new copy number variation and the evaluation of a CNV detection tool for NGS panel data in polish familial hypercholesterolemia patients. Genes. 2022;13(8):1424. 36011335 10.3390/genes13081424 PMC9407502

[49] Hobbs HH , Brown MS , Goldstein JL . Molecular genetics of the LDL receptor gene in familial hypercholesterolemia. Hum Mutat. 1992;1(6):445‐466. 1301956 10.1002/humu.1380010602

[50] Hobbs HH , Brown MS , Russell DW , Davignon J , Goldstein JL . Deletion in the gene for the low‐density‐lipoprotein receptor in a majority of French Canadians with familial hypercholesterolemia. N Engl J Med. 1987;317(12):734‐737. 3627182 10.1056/NEJM198709173171204

[51] Aalto‐Setälä K , Helve E , Kovanen P , Kontula K . Finnish type of low density lipoprotein receptor gene mutation (FH‐Helsinki) deletes exons encoding the carboxy‐terminal part of the receptor and creates an internalization‐defective phenotype. J Clin Invest. 1989;84(2):499‐505. 2760198 10.1172/JCI114192 PMC548909

[52] Aalto‐Setälä K , Koivisto UM , Miettinen T , et al. Prevalence and geographical distribution of major LDL receptor gene rearrangements in Finland. J Intern Med. 1992;231(3):227‐234. 1372927 10.1111/j.1365-2796.1992.tb00528.x

[53] Lehrman MA , Schneider WJ , Südhof TC , Brown MS , Goldstein JL , Russell DW . Mutation in LDL receptor: Alu–Alu recombination deletes exons encoding transmembrane and cytoplasmic domains. Science. 1985;227(4683):140‐146. 3155573 10.1126/science.3155573 PMC4449727

[54] Horsthemke B , Beisiegel U , Dunning A , Havinga JR , Williamson R , Humphries S . Unequal crossing‐over between two alu‐repetitive DNA sequences in the low‐density‐lipoprotein‐receptor gene: a possible mechanism for the defect in a patient with familial hypercholesterolaemia. Eur J Biochem. 1987;164(1):77‐81. 3549308 10.1111/j.1432-1033.1987.tb10995.x

[55] Goldmann R , Tichý L , Freiberger T , et al. Genomic characterization of large rearrangements of the LDLR gene in Czech patients with familial hypercholesterolemia. BMC Med Genet. 2010;11(1):1‐8. 20663204 10.1186/1471-2350-11-115 PMC2923121

[56] Iacocca M , Wang J , Dron J , et al. DNA copy number variation screening in familial hypercholesterolemia‐related genes. Atherosclerosis. 2018;275:e79.

[57] Zhou Y , Mägi R , Milani L , Lauschke VM . Global genetic diversity of human apolipoproteins and effects on cardiovascular disease risk [S]. J Lipid Res. 2018;59(10):1987‐2000. 30076208 10.1194/jlr.P086710 PMC6168301

[58] Iacocca MA , Wang J , Sarkar S , et al. Whole‐gene duplication of PCSK9 as a novel genetic mechanism for severe familial hypercholesterolemia. Can J Cardiol. 2018;34(10):1316‐1324. 30269829 10.1016/j.cjca.2018.07.479

[59] Wexler R , Elton T , Pleister A , Feldman D . Cardiomyopathy: an overview. Am Fam Physician. 2009;79(9):778. PMC2999879 20141097

[60] Ciarambino T , Menna G , Sansone G , Giordano M . Cardiomyopathies: an overview. Int J Mol Sci. 2021;22(14):7722. 34299342 10.3390/ijms22147722 PMC8303989

[61] Cimiotti D , Budde H , Hassoun R , Jaquet K . Genetic restrictive cardiomyopathy: causes and consequences—an integrative approach. Int J Mol Sci. 2021;22(2):558. 33429969 10.3390/ijms22020558 PMC7827163

[62] Norton N , Li D , Rieder MJ , et al. Genome‐wide studies of copy number variation and exome sequencing identify rare variants in BAG3 as a cause of dilated cardiomyopathy. Am J Hum Genet. 2011;88(3):273‐282. 21353195 10.1016/j.ajhg.2011.01.016 PMC3059419

[63] Mademont‐Soler I , Mates J , Yotti R , et al. Additional value of screening for minor genes and copy number variants in hypertrophic cardiomyopathy. PLoS One. 2017;12(8):e0181465. 28771489 10.1371/journal.pone.0181465 PMC5542623

[64] Lopes L , Murphy C , Syrris P , et al. Use of high‐throughput targeted exome‐sequencing to screen for copy number variation in hypertrophic cardiomyopathy. Eur J Med Genet. 2015;58(11):611‐616. 26455666 10.1016/j.ejmg.2015.10.001

[65] Ceyhan‐Birsoy O , Pugh TJ , Bowser MJ , et al. Next generation sequencing‐based copy number analysis reveals low prevalence of deletions and duplications in 46 genes associated with genetic cardiomyopathies. Mol Genet Genomic Med. 2016;4(2):143‐151. 27066507 10.1002/mgg3.187 PMC4799872

[66] Singer ES , Ross SB , Skinner JR , et al. Characterization of clinically relevant copy‐number variants from exomes of patients with inherited heart disease and unexplained sudden cardiac death. Genet Med. 2021;23(1):86‐93. 32973354 10.1038/s41436-020-00970-5

[67] Fedida J , Fressart V , Charron P , et al. Contribution of exome sequencing for genetic diagnostic in arrhythmogenic right ventricular cardiomyopathy/dysplasia. PLoS One. 2017;12(8):e0181840. 28767663 10.1371/journal.pone.0181840 PMC5540585

[68] Mates J , Mademont‐Soler I , Del Olmo B , et al. Role of copy number variants in sudden cardiac death and related diseases: genetic analysis and translation into clinical practice. Eur J Hum Genet. 2018;26(7):1014‐1025. 29511324 10.1038/s41431-018-0119-1 PMC6018743

[69] Toruner GA , Kurvathi R , Sugalski R , et al. Copy number variations in three children with sudden infant death. Clin Genet. 2009;76(1):63‐68. 19659761 10.1111/j.1399-0004.2009.01161.x

[70] Eastaugh LJ , James PA , Phelan DG , Davis AM . Brugada syndrome caused by a large deletion in SCN5A only detected by multiplex ligation‐dependent probe amplification. J Cardiovasc Electrophysiol. 2011;22(9):1073‐1076. 21288276 10.1111/j.1540-8167.2010.02003.x

[71] Campuzano Larrea O , Sarquella Brugada G , Mademont Soler I , et al. Identification of genetic alterations, as causative genetic defects in long QT syndrome, using next generation sequencing technology. PLoS One. 2014;9:e114894. 25494010 10.1371/journal.pone.0114894 PMC4262446

[72] Truszkowska GT , Bilińska ZT , Kosińska J , et al. A study in polish patients with cardiomyopathy emphasizes pathogenicity of phospholamban (PLN) mutations at amino acid position 9 and low penetrance of heterozygous null PLN mutations. BMC Med Genet. 2015;16(1):1‐9. 25928149 10.1186/s12881-015-0167-0 PMC4421997

[73] Campbell MJ , Czosek RJ , Hinton RB , Miller EM . Exon 3 deletion of ryanodine receptor causes left ventricular noncompaction, worsening catecholaminergic polymorphic ventricular tachycardia, and sudden cardiac arrest. Am J Med Genet A. 2015;167(9):2197‐2200. 10.1002/ajmg.a.37140 26018045

[74] Sonoda K , Ohno S , Otuki S , et al. Quantitative analysis of PKP2 and neighbouring genes in a patient with arrhythmogenic right ventricular cardiomyopathy caused by heterozygous PKP2 deletion. EP Europace. 2017;19(4):644‐650. 10.1093/europace/euw038 28431057

[75] Zipes DP , Wellens HJ . Sudden cardiac death. Circulation. 1998;98(21):2334‐2351. 9826323 10.1161/01.cir.98.21.2334

[76] Kim J‐B . Channelopathies. Korean J Pediatr. 2014;57(1):1‐18. 24578711 10.3345/kjp.2014.57.1.1 PMC3935107

[77] Wang T , Chen L , Yang T , et al. Congenital heart disease and risk of cardiovascular disease: a meta‐analysis of cohort studies. J Am Heart Assoc. 2019;8(10):e012030. 31070503 10.1161/JAHA.119.012030 PMC6585327

[78] Costain G , Silversides CK , Bassett AS . The importance of copy number variation in congenital heart disease. NPJ Genom Med. 2016;1(1):1‐11. 10.1038/npjgenmed.2016.31 PMC5505728 28706735

[79] Ehrlich L , Prakash SK . Copy‐number variation in congenital heart disease. Curr Opin Genet Dev. 2022;77:101986. 36202051 10.1016/j.gde.2022.101986

[80] Liu Y , Chang X , Glessner J , et al. Association of rare recurrent copy number variants with congenital heart defects based on next‐generation sequencing data from family trios. Front Genet. 2019;10:819. 31552105 10.3389/fgene.2019.00819 PMC6746959

[81] Glessner JT , Bick AG , Ito K , et al. Increased frequency of de novo copy number variants in congenital heart disease by integrative analysis of single nucleotide polymorphism array and exome sequence data. Circ Res. 2014;115(10):884‐896. 25205790 10.1161/CIRCRESAHA.115.304458 PMC4209190

[82] Saacks NA , Eales J , Spracklen TF , et al. Investigation of copy number variation in south African patients with congenital heart defects. Circ: Genom Precis Med. 2022;15(6):e003510. 36205932 10.1161/CIRCGEN.121.003510 PMC9770125

[83] Fakhro KA , Choi M , Ware SM , et al. Rare copy number variations in congenital heart disease patients identify unique genes in left‐right patterning. Proc Natl Acad Sci. 2011;108(7):2915‐2920. 21282601 10.1073/pnas.1019645108 PMC3041108

[84] Breckpot J , Thienpont B , Arens Y , et al. Challenges of interpreting copy number variation in syndromic and non‐syndromic congenital heart defects. Cytogenet Genome Res. 2011;135(3–4):251‐259. 21921585 10.1159/000331272

[85] Yang H , Shen H , Zhu G , et al. Molecular characterization and clinical investigation of patients with heritable thoracic aortic aneurysm and dissection. J Thorac Cardiovasc Surg. 2023;166(6):1594‐1603. 36517271 10.1016/j.jtcvs.2022.11.004

[86] Erhart P , Gieldon L , Ante M , et al. Acute Stanford type B aortic dissection—who benefits from genetic testing? J Thorac Dis. 2020;12(11):6806‐6812. 33282382 10.21037/jtd-20-2421 PMC7711383

[87] Overwater E , Marsili L , Baars MJ , et al. Results of next‐generation sequencing gene panel diagnostics including copy‐number variation analysis in 810 patients suspected of heritable thoracic aortic disorders. Hum Mutat. 2018;39(9):1173‐1192. 29907982 10.1002/humu.23565 PMC6175145

[88] Campens L , Callewaert B , Muiño Mosquera L , et al. Gene panel sequencing in heritable thoracic aortic disorders and related entities–results of comprehensive testing in a cohort of 264 patients. Orphanet J Rare Dis. 2015;10(1):1‐9. 25644172 10.1186/s13023-014-0221-6 PMC4326194

[89] Prakash S , Kuang S‐Q , Investigators GR , Regalado E , Guo D , Milewicz D . Recurrent rare genomic copy number variants and bicuspid aortic valve are enriched in early onset thoracic aortic aneurysms and dissections. PLoS One. 2016;11(4):e0153543. 27092555 10.1371/journal.pone.0153543 PMC4836726

[90] Charchar FJ , Kaiser M , Bingham AJ , et al. Whole genome survey of copy number variation in the spontaneously hypertensive rat: relationship to quantitative trait loci, gene expression, and blood pressure. Hypertension. 2010;55(5):1231‐1238. 20231529 10.1161/HYPERTENSIONAHA.109.141663 PMC5266550

[91] Marques FZ , Prestes PR , Pinheiro LB , et al. Measurement of absolute copy number variation reveals association with essential hypertension. BMC Med Genet. 2014;7:44. 10.1186/1755-8794-7-44 PMC4107748 25027169

[92] Petrovich M , Ivanga M , Seda O , et al. Copy number variation analysis in hypertension and its associated metabolic components. University of Montreal; 2007.

[93] Mat Jusoh JA . The prevalence and functional impact of rare genomic copy number variation in hypertension‐related left ventricular hypertrophy. Universiti Teknologi MARA; 2020.

[94] Shia W‐C , Ku T‐H , Tsao Y‐M , et al. Genetic copy number variants in myocardial infarction patients with hyperlipidemia. BMC Genomics. 2011;12:1‐8. 10.1186/1471-2164-12-S3-S23 PMC3333183 22369086

[95] Buysse K , Delle Chiaie B , Van Coster R , et al. Challenges for CNV interpretation in clinical molecular karyotyping: lessons learned from a 1001 sample experience. Eur J Med Genet. 2009;52(6):398‐403. 19765681 10.1016/j.ejmg.2009.09.002

[96] Pös O , Radvanszky J , Styk J , et al. Copy number variation: methods and clinical applications. Appl Sci. 2021;11(2):819.

[97] Shen Y , Wu B‐L . Designing a simple multiplex ligation‐dependent probe amplification (MLPA) assay for rapid detection of copy number variants in the genome. J Genet Genomics. 2009;36(4):257‐265. 19376486 10.1016/S1673-8527(08)60113-7

[98] Carter NP . Methods and strategies for analyzing copy number variation using DNA microarrays. Nat Genet. 2007;39(Suppl 7):S16‐S21. 17597776 10.1038/ng2028 PMC2697494

[99] Serin Harmanci A , Harmanci AO , Zhou X . CaSpER identifies and visualizes CNV events by integrative analysis of single‐cell or bulk RNA‐sequencing data. Nat Commun. 2020;11(1):89. 31900397 10.1038/s41467-019-13779-x PMC6941987

[100] Jansen FA , Blumenfeld YJ , Fisher A , et al. Array comparative genomic hybridization and fetal congenital heart defects: a systematic review and meta‐analysis. Ultrasound Obstet Gynecol. 2015;45(1):27‐35. 25319878 10.1002/uog.14695

[101] Conlin LK , Thiel BD , Bonnemann CG , et al. Mechanisms of mosaicism, chimerism and uniparental disomy identified by single nucleotide polymorphism array analysis. Hum Mol Genet. 2010;19(7):1263‐1275. 20053666 10.1093/hmg/ddq003 PMC3146011

[102] Ma J , Ai X , Wang J , et al. Multiplex ligation‐dependent probe amplification identifies copy number changes in normal and undetectable karyotype MDS patients. Ann Hematol. 2021;100(9):2207‐2214. 33990890 10.1007/s00277-021-04550-8 PMC8357724

[103] Yang Y , Xia C , Zhou Z , et al. A multiplex ligation‐dependent probe amplification‐based next‐generation sequencing approach for the detection of copy number variations in the human genome. Mol Med Rep. 2018;18(6):5823‐5833. 30365071 10.3892/mmr.2018.9581

[104] Eijk‐Van Os PG , Schouten JP . Multiplex ligation‐dependent probe amplification (MLPA®) for the detection of copy number variation in genomic sequences. Methods Mol Biol. 2011;688:97‐126. 20938835 10.1007/978-1-60761-947-5_8

[105] Fan J , Lee H‐O , Lee S , et al. Linking transcriptional and genetic tumor heterogeneity through allele analysis of single‐cell RNA‐seq data. Genome Res. 2018;28(8):1217‐1227. 29898899 10.1101/gr.228080.117 PMC6071640

[106] Tirosh I , Izar B , Prakadan SM , et al. Dissecting the multicellular ecosystem of metastatic melanoma by single‐cell RNA‐seq. Science. 2016;352(6282):189‐196. 27124452 10.1126/science.aad0501 PMC4944528

[107] Zhao H , Huang T , Li J , Liu G , Yuan X . MFCNV: a new method to detect copy number variations from next‐generation sequencing data. Front Genet. 2020;11:434. 32499814 10.3389/fgene.2020.00434 PMC7243272

[108] Singh AK , Olsen MF , Lavik LAS , Vold T , Drabløs F , Sjursen W . Detecting copy number variation in next generation sequencing data from diagnostic gene panels. BMC Med Genet. 2021;14(1):214. 10.1186/s12920-021-01059-x PMC8406611 34465341

[109] Ellingford JM , Campbell C , Barton S , et al. Validation of copy number variation analysis for next‐generation sequencing diagnostics. Eur J Hum Genet. 2017;25(6):719‐724. 28378820 10.1038/ejhg.2017.42 PMC5427176

[110] Wang H , Nettleton D , Ying K . Copy number variation detection using next generation sequencing read counts. BMC Bioinformatics. 2014;15(1):1‐14. 24731174 10.1186/1471-2105-15-109 PMC4021345

[111] Chen Y , Zhao L , Wang Y , et al. SeqCNV: a novel method for identification of copy number variations in targeted next‐generation sequencing data. BMC Bioinform. 2017;18(1):1‐9. 10.1186/s12859-017-1566-3 PMC5335817 28253855

[112] Magi A , Benelli M , Gozzini A , Girolami F , Torricelli F , Brandi ML . Bioinformatics for next generation sequencing data. Genes. 2010;1(2):294‐307. 24710047 10.3390/genes1020294 PMC3954090

[113] Dalca AV , Brudno M . Genome variation discovery with high‐throughput sequencing data. Brief Bioinform. 2010;11(1):3‐14. 20053733 10.1093/bib/bbp058

[114] Roca I , González‐Castro L , Fernández H , Couce ML , Fernández‐Marmiesse A . Free‐access copy‐number variant detection tools for targeted next‐generation sequencing data. Mutat Res/Rev Mutat Res. 2019;779:114‐125. 31097148 10.1016/j.mrrev.2019.02.005

[115] Chu J‐h , Rogers A , Ionita‐Laza I , et al. Copy number variation genotyping using family information. BMC Bioinform. 2013;14(1):157. 10.1186/1471-2105-14-157 PMC3668900 23656838

[116] Tuzun E , Sharp AJ , Bailey JA , et al. Fine‐scale structural variation of the human genome. Nat Genet. 2005;37(7):727‐732. 15895083 10.1038/ng1562

[117] Capalbo A , Rienzi L , Ubaldi FM . Diagnosis and clinical management of duplications and deletions. Fertil Steril. 2017;107(1):12‐18. 28040093 10.1016/j.fertnstert.2016.11.002

[118] De Vries BB , Pfundt R , Leisink M , et al. Diagnostic genome profiling in mental retardation. Am J Hum Genet. 2005;77(4):606‐616. 16175506 10.1086/491719 PMC1275609

[119] Arbustini E , Behr ER , Carrier L , et al. Interpretation and actionability of genetic variants in cardiomyopathies: a position statement from the European Society of Cardiology Council on cardiovascular genomics. Eur Heart J. 2022;43(20):1901‐1916. 35089333 10.1093/eurheartj/ehab895

[120] Muller RD , McDonald T , Pope K , Cragun D . Evaluation of clinical practices related to variants of uncertain significance results in inherited cardiac arrhythmia and inherited cardiomyopathy genes. Circ: Genom Precis Med. 2020;13(4):e002789. 32522017 10.1161/CIRCGEN.119.002789

[121] Musunuru K , Hershberger R , Day S , et al. Genetic testing for inherited cardiovascular diseases: a scientific statement from the American Heart Association. Circ Genom Precis Med. 2020;13(4):e000067. 32698598 10.1161/HCG.0000000000000067

[122] Parrotta EI , Lucchino V , Scaramuzzino L , Scalise S , Cuda G . Modeling cardiac disease mechanisms using induced pluripotent stem cell‐derived cardiomyocytes: progress, promises and challenges. Int J Mol Sci. 2020;21(12):4354. 32575374 10.3390/ijms21124354 PMC7352327

[123] Garg P , Oikonomopoulos A , Chen H , et al. Genome editing of induced pluripotent stem cells to decipher cardiac channelopathy variant. J Am Coll Cardiol. 2018;72(1):62‐75. 29957233 10.1016/j.jacc.2018.04.041 PMC6050025

[124] Chen E , Facio FM , Aradhya KW , et al. Rates and classification of variants of uncertain significance in hereditary disease genetic testing. JAMA Netw Open. 2023;6(10):e2339571. 37878314 10.1001/jamanetworkopen.2023.39571 PMC10600581

[125] Stephensen SS , Sigfusson G , Eiriksson H , et al. Congenital cardiac malformations in Iceland from 1990 through 1999. Cardiol Young. 2004;14(4):396‐401. 15680046 10.1017/S1047951104004081

[126] Weaver KN , Chen J , Shikany A , et al. Prevalence of genetic diagnoses in a cohort with valvar pulmonary stenosis. Circ: Genom Precis Med. 2022;15(4):e003635. 35666834 10.1161/CIRCGEN.121.003635 PMC9388589

[127] Zhang F , Gu W , Hurles ME , Lupski JR . Copy number variation in human health, disease, and evolution. Annu Rev Genomics Hum Genet. 2009;10:451‐481. 19715442 10.1146/annurev.genom.9.081307.164217 PMC4472309

